# Metal-free oxidative isocyanides insertion with aromatic aldehydes to aroylated N-heterocycles†

**DOI:** 10.1039/c7ra12755c

**Published:** 2018-01-15

**Authors:** Yimiao He, Xuelian Wang, Jun-An Xiao, Jinying Pang, Chunfang Gan, Yanmin Huang, Chusheng Huang

**Affiliations:** College of Chemistry and Materials Science, Guangxi Teachers Education University Nanning 530001 P. R. China heyimiao@gxtc.edu.cn huangcs@gxtc.edu.cn

## Abstract

A metal-free, mild and efficient protocol for the construction of aroylated N-heterocycles *via* radical cascade aroylation of phenyl or vinyl isocyanides with aromatic aldehydes has been developed. Both phenanthridine and isoquinoline derivatives are delivered quickly in moderate to good yields with high regioselectivity.

## Abstract 2

A metal-free, mild and efficient method for construction of aroylated phenanthridines and isoquinolines quickly with high regioselectivity was developed.

## Graphical abstract

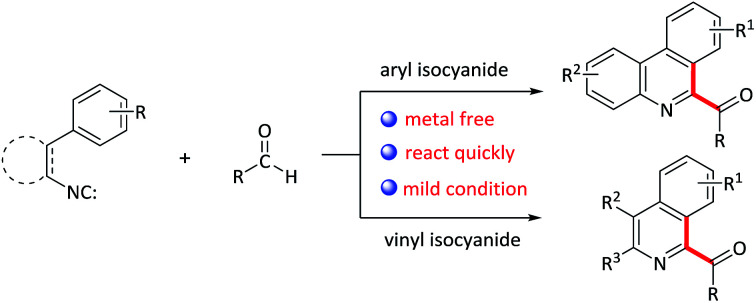

Acylation reactions are a powerful tool for the synthesis of carbonyl compounds, which are abundant in natural products, pharmaceuticals, and dye and agrichemical industries.^1^ Traditional acylation is Friedel–Crafts reaction (scheme 1a),^2^ which proceeds by reacting arenes with acyl halides in the presence of Lewis or Brønsted acids. However, this methodology also suffers from poor regioselectivity, the use of stoichiometric amounts of reactive acyl halide reagents as well as electron-rich arenes, which restrict its extensive application in organic synthesis. Recently, great progresses have been made for C–C bond formations directly from two different C–H bonds under oxidative conditions that is termed the cross dehydrogenative coupling (CDC reaction),^3,4^ representing the state-of-art in C–C bond-formation reactions. Such a coupling allows the use of simple nonfunctionalized substrates, thus making the synthesis shorter and more efficient. However, in contrast to the acylation of electron-rich aromatic compounds, very few methods are available for the acylation of electron-deficient heterocycles. Among these methods, the addition of nucleophilic acyl radicals to electron-deficient heterocyclic aromatic bases, that is, the Minisci reaction, is a commonly used approach (scheme 1b).^5,6^ Very recently, Antonchick *et al.* described a PhI(OCOCF_3_)_2_-mediated cross-dehydrogenative coupling of heterocycles with aldehydes to construct acylated N-heterocycles (scheme 1b).^7^ Compared with these previous works, the development of novel tandem reaction would be a better choice, as it allows to construct a series of acylated nitrogen-containing heterocycles in one pot.

**Scheme 1:**
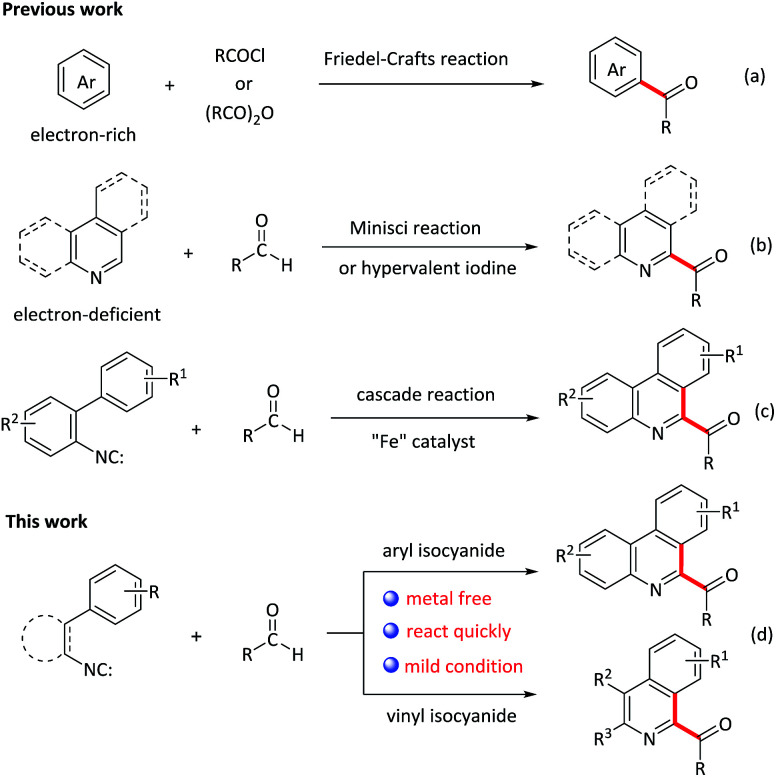
Strategies for the acylation reactions.

Isocyanides are the versatile building blocks in organic synthesis due to their special structural and reactive properties, and have been widely applied in the formation of N-heterocycles.^8^ Along with transition-metal catalyzed isocyanides insertion, isocyanides are used as radical receptors with subsequent homolytic aromatic substitution to construct substituted N-heterocycles recently have also been realized.^9^ For example, Studer and co-workers reported an efficient synthesis of 6-aroylated phenanthridines through base promoted homolytic aromatic substitution of 2-isocyanobiphenyls with aromatic aldehydes using FeCl_3_ as an initiator.^10^ However, this method also suffers from transition metal residues, harsh conditions and long time. Moreover, compared to the phenyl isocyanides, the addition of acyl radicals derived from aldehydes to vinyl isocyanides^11^ and finally receive 1-acylated isoquinolines, to our knowledge, has not been realised. Considering the importance of both N-heterocycles and acyl groups, herein, we elaborate a quick oxidative isocyanide insertion reaction with aromatic aldehydes through PIDA/TMSN_3_-mediated C–C bond formation, delivering an efficient approach to construction of 6-aroylated phenanthridine and 1-aroylated isoquinoline derivatives in one pot (Scheme 1d).

Initially, the reaction was carried out by using 2-isocyanobiphenyl 1a and benzaldehyde 2a as model substrates in benzene with PIFA as the oxidant and TMSN_3_ as the additive at room temperature (Table 1, entry 1). Pleasingly, the reaction of 1a with 4 equiv. of 2a in the presence of 2 equiv. of PIFA with TMSN_3_ to give the desired 3a in 23% yield. After screening various solvents, CH_2_Cl_2_ was found to be the most efficient and provided 3a in 27% yield (entries 2–4). Furthermore, replacing the additive TMSN_3_ with NaN_3_ didn't improve the reaction (entry 5). Subsequently, various oxidants were also evaluated and PIDA gave a better result, providing 3a in 31% yield (entries 6–8). Importantly, a further improvement was achieved upon elevating the temperature to 50 °C (entry 9). However, further increasing the temperature resulted in the yield reduction, maybe due to the aldehyde being oxidized to carboxylate under harsh conditions. We also investigated the relative ratio of reactants (entries 10–13), and found that the addition of the amounts of aldehydes to 6 equivalent amounts along with oxidants and additives to 2.5 equivalent amounts led to the optimum result and gave 3a in 74% yield (entry 12). The blank experiments demonstrated that both oxidants and additives were essential for the isocyanide insertion reactions (entries 14, 15). Consequently, the optimum reaction conditions were determined to be 2-isocyanobiphenyl 1a with aldehyde 2a (6 equiv.) in the presence of PIDA as well as TMSN_3_ (2.5 equiv.) at 50 °C under air condition. It was noteworthy that the reaction proceeded without any metal catalysts in only 15 minutes.

**Table 1:** Optimization of reaction conditions^a^

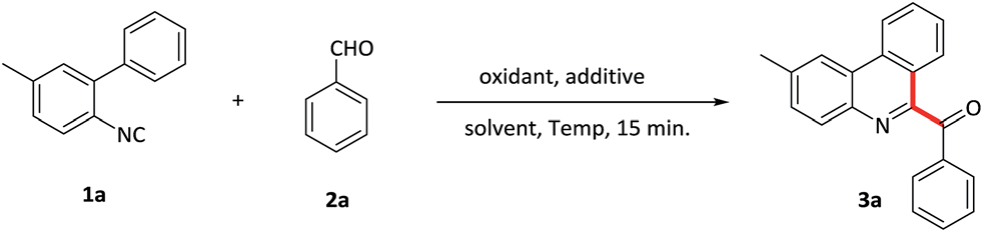					
Entry	Solvent	Oxidant	Additive	*T* [°C]	Yield^b^ [%]
1	Benzene	PIFA	TMSN_3_	25	23
2	CH_2_Cl_2_	PIFA	TMSN_3_	25	27
3	DCE	PIFA	TMSN_3_	25	22
4	CH_3_CN	PIFA	TMSN_3_	25	n.d.
5	CH_2_Cl_2_	PIFA	NaN_3_	25	25
6	CH_2_Cl_2_	PIDA	TMSN_3_	25	31
7	CH_2_Cl_2_	PhI(OPiv)_2_	TMSN_3_	25	n.d.
8	CH_2_Cl_2_	TBHP	TMSN_3_	25	n.d.
9	CH_2_Cl_2_	PIDA	TMSN_3_	50	47
10^c^	CH_2_Cl_2_	PIDA	TMSN_3_	50	52
11^c^^,^^d^	CH_2_Cl_2_	PIDA	TMSN_3_	50	61
**12** c ^,^ e	**CH**_**2**_ **Cl**_**2**_	**PIDA**	**TMSN**_**3**_	**50**	**74**
13^c^^,^^f^	CH_2_Cl_2_	PIDA	TMSN_3_	50	71
14^e^	CH_2_Cl_2_	None	TMSN_3_	50	n.d.
15^e^	CH_2_Cl_2_	PIDA	None	50	n.d.

a Conditions: 1a (0.2 mmol), 2a (0.8 mmol), oxidant (0.4 mmol), additive (0.4 mmol) in solvent (2 mL) for 15 min. PIDA = phenyliodine(iii) diacetate, PIFA = phenyliodine(iii) bis(tri-fluoroacetate).

b Isolated yields.

c Oxidant (0.5 mmol), additive (0.5 mmol).

d 2a (1.0 mmol).

e 2a (1.2 mmol).

f 2a (1.4 mmol).

With optimized conditions in hand, various benzaldehydes 2a–l were first tested to react with 2-isocyanobiaryl 1a to form the corresponding 6-aroyl phenanthridine products 3a–l. As showed in Table 2, benzaldehydes bearing electron-rich groups (2b–d) such as methyl, ethyl and methoxy at the *para*-position underwent this oxidative isocyanide insertion process smoothly to afford the desired products in moderate to good yields (63–76%). Weakly electron-withdrawing groups, for example, fluoro (2e) and chloro (2f) substituents, gave lower yield. When the benzaldehyde moiety was substituted by strongly electron-withdrawing group such as CF_3_ (2g) at the *para*-position, the yield was sharply decreased and only 13% yield of 3g was obtained (2g was recovered in 82% yield). These results indicated that electronic effects exerted by the substituents on the arene ring of the benzaldehyde component had an important influence on this reaction. Moreover, lower yields were achieved for the *ortho*-substituents maybe due to the steric effect (3h–j). In addition, *meta*-substituted groups also gave the corresponding products 3k and 3l in moderate yield.

**Table 2:** Variation of the aldehyde component^a^

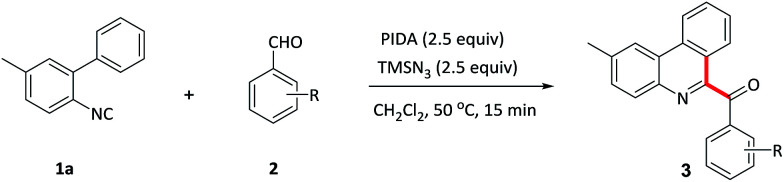
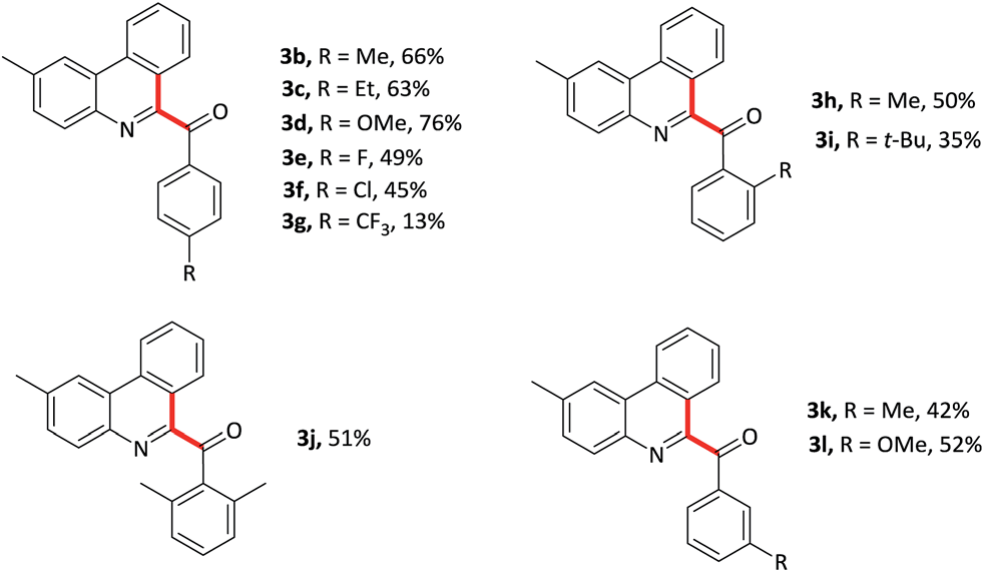

a Reaction conditions: 1a (0.2 mmol), 2 (1.2 mmol), PIDA (0.5 mmol), TMSN_3_ (0.5 mmol) in CH_2_Cl_2_ (2 mL) at 50 °C for 15 min.

The isocyanobiaryl moiety was subsequently evaluated (Table 3). Various substituents on the arene ring without the isocyanide group at the 4-position were tolerant well, affording the desired products in moderate to good yield. For example, electron-donating groups such as *t*-Bu, OMe, OBn gave the product 3m–o in 47–76% yield. Electron-withdrawing F, CF_3_ substituents also formed the product 3p and 3q in 87% and 62% yield, respectively. To explore the regioselectivity, 3-methylisocyanidebiphenyl 1r was employed as substrate to proceed this oxidative isocyanide insertion process, and gave a mixture of two isomers 3r and 3r′, the ratio of which was 1.8 : 1. *Ortho*-methyl derivative 1s gave the targeted product 3s in a slight lower yield maybe owing to the steric effect. In addition, the substituent effects on the aromatic ring attached to isocyanide moiety were also been explored. Not only the electron-rich group such as methyl substituent but also the electron-withdrawing groups such as chloro and trifluoromethyl substituents were compatible with this oxidative cyclization process, and formed the products 3u–w in 62–81% yield. Importantly, halogen substituent could be used as a coupling partner for further transformation.

**Table 3:** Variation of the 2-isocyanobiaryl component^a^

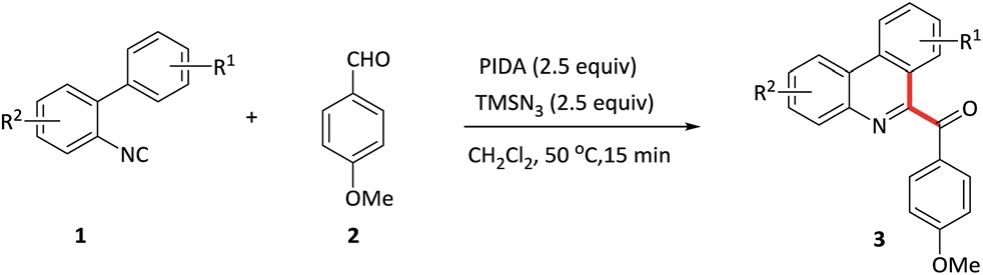
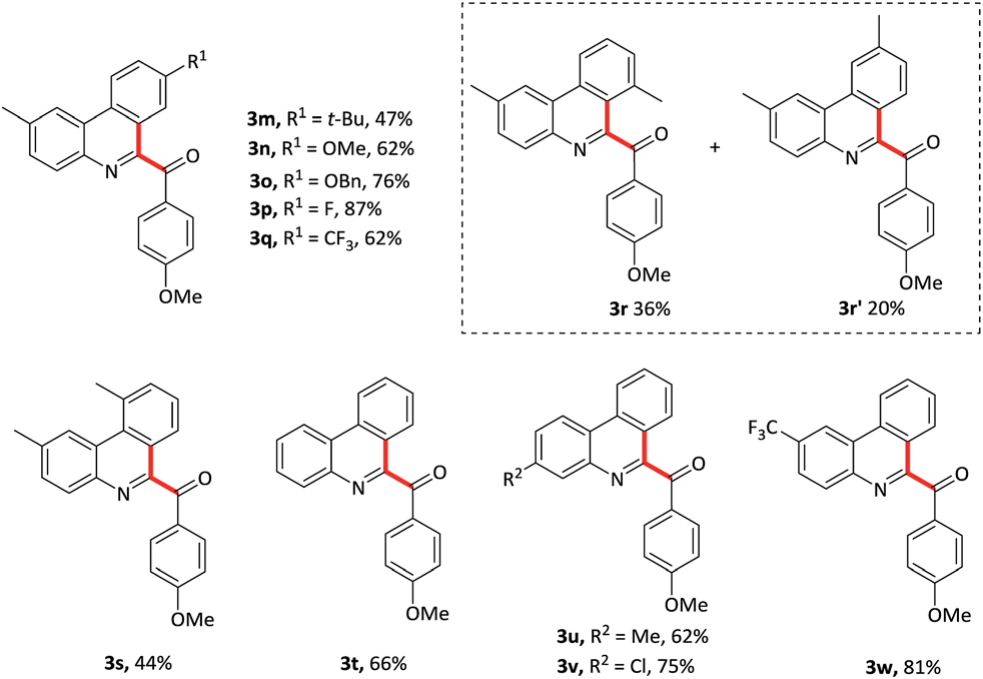

a Reaction conditions: 1 (0.2 mmol), 2 (1.2 mmol), PIDA (0.5 mmol), TMSN_3_ (0.5 mmol) in CH_2_Cl_2_ (2 mL) at 50 °C for 15 min.

Vinyl isocyanides were also explored. Methyl-4-aryl-1-aroyl-isoquiniline-3-carboxylates 5a–d were prepared in moderate yields starting from the corresponding vinyl isocyanides and 4-methoxybenzaldehyde 2. Furthermore, 4-methyl substituent isoquinoline 5e was also formed in a slightly lower yield (41%).

To investigate the mechanism for this oxidative isocyanide insertion reaction, several control experiments were conducted. Benzaldehyde 6 containing *ortho* terminal alkene was first reacted with 1a under the standard conditions (Scheme 2a). Phenanthridine derivatives 7, attached by 2,3-dihydro-1*H*-inden-1-one with a methylene linkage, was formed through acyl radical addition to the intramolecular C–C double bond, followed by the reaction with isocyanide 1a. It provided a novel and efficient approach for the synthesis of multi heterocyclic systems bearing a phenanthridine moiety. Furthermore, when TEMPO, a radical scavenge, was added into the system, the reaction was suppressed and the starting material 1a was recovered in 90% yield (Scheme 2b). These results indicated that the reaction probably involved a radical process (Table 4).

**Scheme 2:**
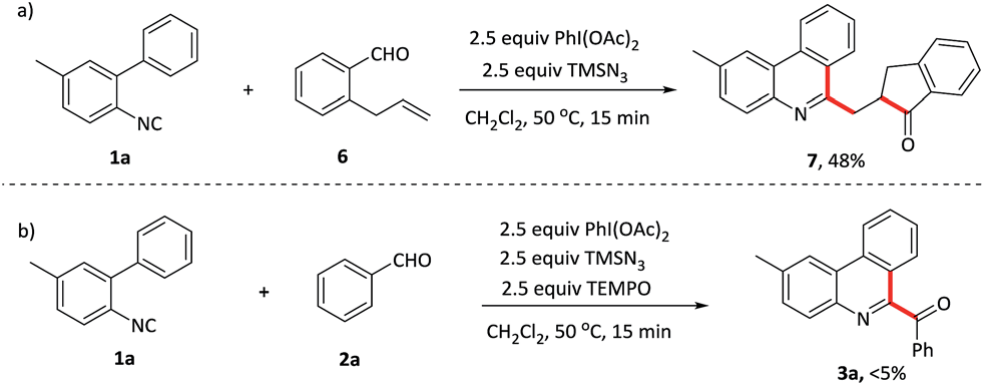
(a) Radical clock experiment. (b) Radical interception experiment.

**Table 4:** Substrate scope of vinyl isocyanides^a^

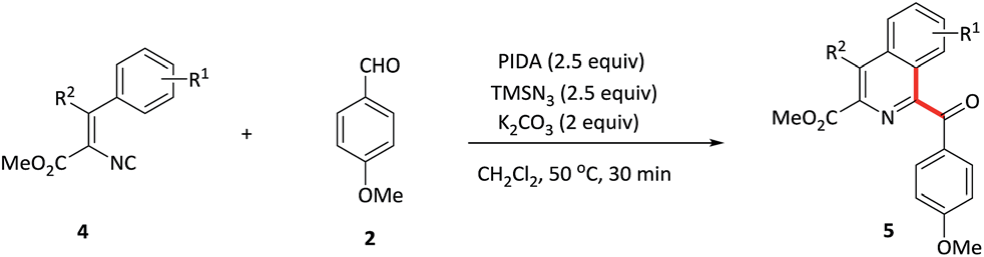
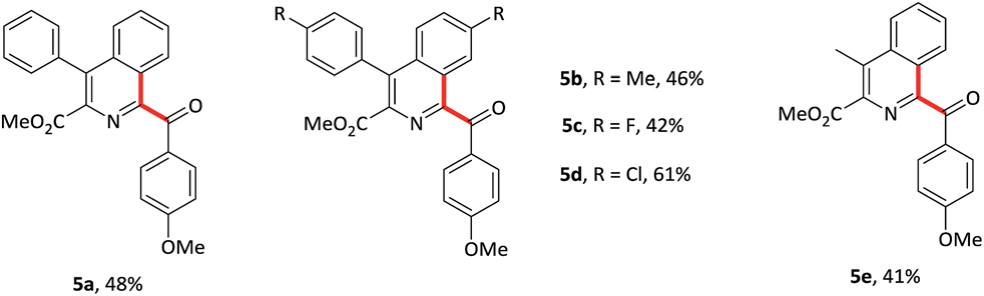

a Reaction conditions: 1 (0.2 mmol), 2 (1.2 mmol), PIDA (0.5 mmol), TMSN_3_ (0.5 mmol) and K_2_CO_3_ (0.4 mmol) in CH_2_Cl_2_ (2 mL) at 50 °C for 30 min.

Based on the above results and previous reports,^7^ a plausible mechanism pathway for PhI(OAc)_2_/N_3_-mediated synthesis of aroylated nitrogen-containing aromatic heterocycles starting from aryl or vinyl isocyanides with aromatic aldehydes is depicted in Scheme 3. Initial ligand exchange between PhI(OAc)_2_ and TMSN_3_ provides hypervalent iodide intermediate A, which next undergoes a thermal homolytic cleavage to generate an azide radical and radical B. Then, the azide radical abstracts an H atom from the aldehyde to release the acyl radical C, which attacks the isocyanide 1 to form the imidoyl radical D. Radical D subsequently experiences a homolytic aromatic substituent followed by an oxidative aromatization process and finally afford the targeted aroylated nitrogen-containing aromatic heterocycles 3.

**Scheme 3:**
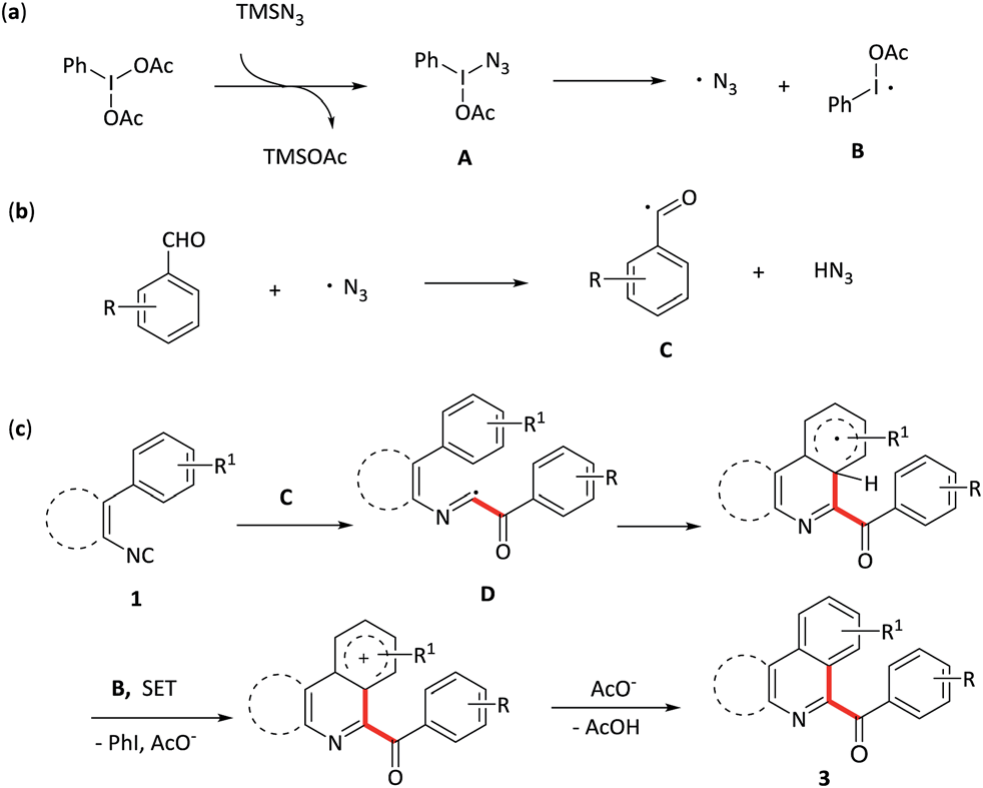
Proposed mechanism.

In summary, we have developed a mild and efficient protocol for the construction of aroylated azaheterocyclic arenes *via* oxidative isocyanide insertion of isocyanides with benzaldehydes under metal-free conditions. Diversified phenanthridine and isoquinoline derivatives are assembled quickly in moderate to good yields with high regioselectivity. Interestingly, benzaldehyde bearing *ortho*-allyl substituent can also be applied in this reaction, allowing the installation of other heterocycle to phenanthridine by a methylene tether. Control experiments reveals that the reaction probably experiences a radical process. Further investigations on the chemistry of isocyanides and biological evaluation of the reductant products are underway in our laboratory.

## Conflicts of interest

There are no conflicts to declare.

## Supplementary Material

RA-008-C7RA12755C-s001
