# Supplementary material for: Metal-free oxidative isocyanides insertion with aromatic aldehydes to aroylated N-heterocycles
Source: RSC Adv. 2018 Jan 15;8(6):3036–40. doi: 10.1039/c7ra12755c (PMC9077706; doi:10.1039/c7ra12755c)
Supplement: RA-008-C7RA12755C-s001 [file RA-008-C7RA12755C-s001.pdf]

Electronic Supplementary Material (ESI) for RSC Advances.
This journal is © The Royal Society of Chemistry 2018

Electronic Supplementary Information (ESI)

Metal-Free Oxidative Isocyanides Insertion with Aromatic Aldehydes to Aroylated *N*-heterocycles

Yimiao He,*[a] Xuelian Wang,[a] Jun-An Xiao,[a] Jinying Pang,[a] Chunfang Gan,[a] Yanmin Huang,[a] Chusheng Huang*[a]

College of Chemistry and Materials Science, Guangxi Teachers Education University, Nanning 530001, P. R. China. E-mail: heyimiao@gxtc.edu.cn; huangcs@gxtc.edu.cn.

Table of contents

1. General information 2
2. Synthesis of isocyanides 2
    2.1 Synthesis of phenyl isocyanides **1a-1w** 2
    2.2 Synthesis of vinyl isocyanides **4a-4e** 3
3. Synthesis of material **6** 4
4. General procedure and product characterization 4
    4.1 General procedure 4
    4.2 Product characterization 6
5. Free-Radical inhibition experiment 18
6. References 19
7. Copies of ^{1}H NMR and ^{13}C NMR spectra 20

1. General information

Reactions were monitored by using thin-layer chromatography (TLC) on commercial silica gel plates (GF254). Visualization of the developed plates was performed under UV lights (254 nm). Flash column chromatography was performed on silica gel (200-300 mesh). ^{1}H and ^{13}C NMR spectra were recorded on Bruker AV300，400 and 500 MHz spectrometers. Chemical shifts (δ) were reported in ppm referenced to the $CDCl_3$ residual peak (δ 7.26) or the DMSO-d_6 residual peak (δ 2.50) for ^{1}H NMR. Chemical shifts of ^{13}C NMR were reported relative to $CDCl_3$ (δ 77.0) or D_6-DMSO (δ 39.5). The following abbreviations were used to describe peak splitting patterns when appropriate: br s = broad singlet, s = singlet, d = doublet, t = triplet, q = quartet, m = multiplet. Coupling constant, *J*, was reported in Hertz unit (Hz). Melting points (mp) were taken on a MEL-TEMP® apparatus and were uncorrected. High resolution mass spectra (HRMS) were obtained on an ESI-LC-MS/MS spectrometer.

2. Synthesis of isocyanides

2.1 Synthesis of phenyl isocyanides 1a-1w

The isocyanide **1a** was prepared according to the reported method.[1]

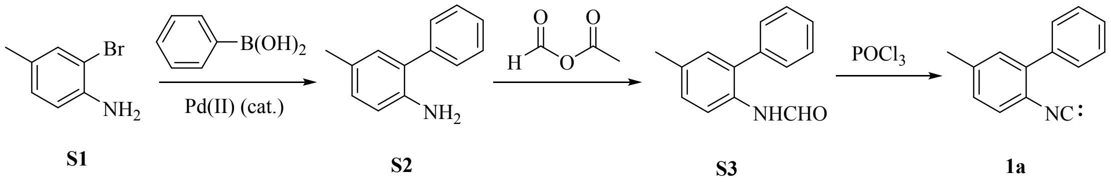

4-Methyl-2-(phenyl)phenyl isocyanide (1a). To an oven-dried three necked flask, 2-bromo-4-methylaniline **S1** (925 mg, 5 mmol), phenylboronic acid (732 mg, 6 mmol), aqueous solution of K_2CO_3 (2M, 11 mL) and DME (10 mL) were added under a gentle stream of Ar, and the mixture was stirred for 30 min at room temperature under Ar atmosphere. $PdCl_2(PPh_3)_2$ (70 mg, 0.10 mmol) was subsequently added at room temperature, and the mixture was stirred overnight at 80 °C under Ar. The reaction mixture was cooled to room temperature and diluted with EtOAc. The organic layer was washed with water and dried over Na_2SO_4. After removing the volatiles in vacuo, the residue was subjected to column chromatography on silica gel (petroleum ether/EtOAc = 4 : 1) to afford 4-methyl-2-phenylaniline **S2** (788 mg, 86% yield).
Acetic formic anhydride (0.89 mL) was added dropwise to a stirred solution of **S2** (788 mg, 4.30 mmol) in THF (8 mL) at 0 °C, and then the mixture was stirred for 2 h

at room temperature. the mixture was quenched by sat. aqueous solution of $NaHCO_3$ and extracted with EtOAc three times. The extract was dried over Na_2SO_4 and concentrated under reduced pressure to give formamide **S3** as a pale yellow oil. This material was used for the subsequent dehydration without further purification.

To an oven-dried three necked flask equipped with a dropping funnel, THF (8 mL), NEt_3 (4.3 mL) and the whole amount of **S3** obtained above were added under Ar atmosphere and cooled to 0 °C. $POCl_3$ (0.7 mL) in 2 mL of THF was added dropwise, and the mixture was stirred for 2 h at 0 °C until the addition was complete. The mixture was slowly quenched by sat. aqueous solution of $NaHCO_3$ at 0 °C and stirred for 1 h at room temperature. The mixture was extracted with EtOAc three times, dried over Na_2SO_4 and evaporated under reduced pressure. The residues were purified by column chromatography (petroleum ether/EtOAc = 30 : 1) to give **1a** as a pale yellow solid (811 mg, 84% yield).

1b-1w were prepared according to the procedure described for **1a**.

2.2 Synthesis of vinyl isocyanides 4a-4e [2]

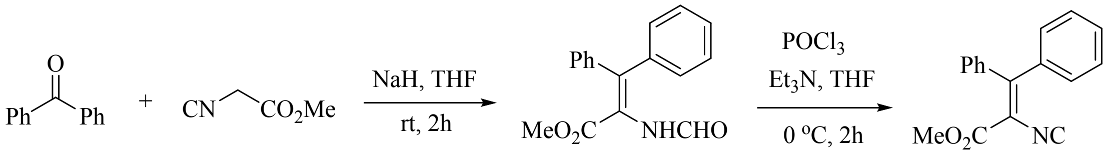

S4 **S5** **S6** **4a**

To a mixture of benzophenone **S4** (0.91 g, 5 mmol) and methyl isocyanoacetate **S5** (0.5 g, 5 mmol) in THF (5 mL), a suspension of NaH (60% in oil) (0.24 g, 6 mmol) in THF (5 ml) was added dropwise at room temperature for 2h. After the reaction was completed (as judged by TLC analysis), the solvent was removed under reduced pressure and the residue was extracted with CH_2Cl_2 three times and the extract was washed with H_2O, dried over Na_2SO_4 and concentrated under reduced pressure. The material **S6** was used for the subsequent dehydration without further purification.

To an oven-dried three necked flask equipped with a dropping funnel, THF (10 mL), NEt_3 (5 mL) and the whole amount of **S6** obtained above were added under Ar atmosphere and cooled to 0 °C. $POCl_3$ (0.8 mL) in 2 mL of THF was added dropwise, and the mixture was stirred for 2 h at 0 °C until the addition was complete. the mixture was slowly quenched by sat. aqueous solution of $NaHCO_3$ at 0 °C and stirred for 1 h at room temperature. The mixture was extracted with EtOAc three times, dried over Na_2SO_4 and evaporated under reduced pressure. The residues were purified by column chromatography (petroleum ether/EtOAc = 30 : 1) to give **4a** as a white solid

(1.2 g, 89% yield).

4b-4e were prepared according to the procedure described for **4a**.

3. Synthesis of material 6 [3]

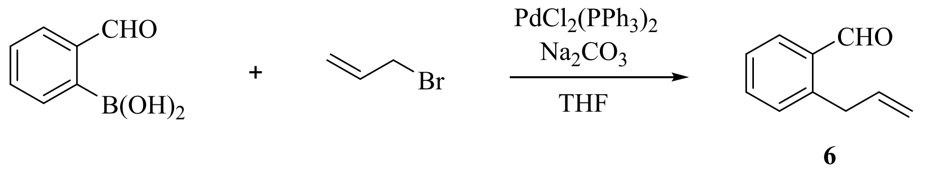

6

a 100 mL round-bottom flask was equipped with a rubber septum and magnetic stir bar and was charged with a solution of 2-formylphenylboronic acid (950 mg, 5 mmol) and allyl bromide (0.5 mL, 6 mmol) in THF (25 mL). Then $PdCl_2(PPh_3)_2$ (88 mg, 0.125 mmol) and aq. Na_2CO_3 (1M, 10 mmol) solution was added. The reaction mixture was heated at reflux for 3-4 h. The reaction mixture was quenched with H_2O and extracted with CH_2Cl_2 three times. The combined organic layers were washed with H_2O, dried over $MgSO_4$, and concentrated in vacuo. The residues were purified by column chromatography on silica gel (petroleum ether/EtOAc = 30:1) to afford the desired product **6** (82% yield) as a pale yellow oil.

4. General procedure and product characterization

4.1 General procedure

Typical procedure for PIDA-mediated oxidative phenyl isocyanides insertion with aromatic aldehydes to 6-aroylated phenanthridines **3a-3w**.

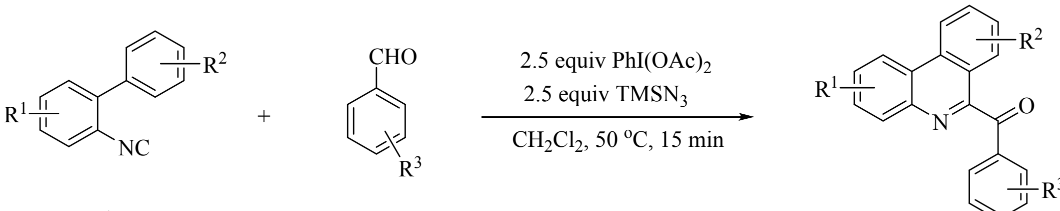

1 2 3

A oven-dried sealed tube was equipped with a magnetic stir bar and was charged with a mixture of 2-isocyanobiphenyl (0.2 mmol), aldehyde (1.2 mmol) and $TMSN_3$ (0.5 mmol) in CH_2Cl_2 (1.0 mL). Then (diacetoxyiodo)benzene (PIDA) (0.5 mmol) was dissolved in CH_2Cl_2 (1.0 mL) and added dropwise to the reaction mixture for 10

minutes at 50 °C. After the reaction was complete (as determined by TLC analysis), the reaction was cooled to room temperature and CH_2Cl_2 (20 mL) was added to the solution and washed with water (20 ml x 3), dried over Na_2SO_4, concentrated under reduced pressure. The crude product was purified by column chromatography on silica gel (petroleum ether/EtOAc = 30:1) to afford the targeted product **3a-3w**.

Typical procedure for PIDA-mediated oxidative vinyl isocyanides insertion with aromatic aldehydes to 1-aroylated isoquinolines **5a-5e**.

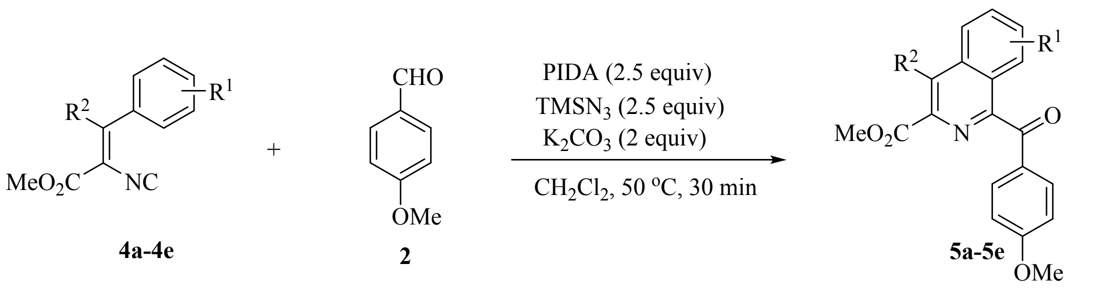

A oven-dried sealed tube was equipped with a magnetic stir bar and was charged with a mixture of vinyl isocyanide (0.2 mmol), aldehyde (1.2 mmol), K_2CO_3 (0.4 mmol) and $TMSN_3$ (0.5 mmol) in CH_2Cl_2 (1.0 mL). Then (diacetoxyiodo)benzene (PIDA) (0.5 mmol) was dissolved in CH_2Cl_2 (1.0 mL) and added dropwise to the reaction mixture for 10 minutes at 50 °C. After the reaction was complete (as determined by TLC analysis), the reaction was cooled to room temperature and CH_2Cl_2 (20 mL) was added to the solution and washed with water (20 ml x 3), dried over Na_2SO_4, concentrated under reduced pressure. The crude product was purified by column chromatography on silica gel (petroleum ether/EtOAc = 30:1) to afford the targeted product **5a-5e**.

Typical procedure for PIDA-mediated oxidative phenyl isocyanide insertion with aromatic aldehyde bearing *ortho* terminal alkene **6** to phenanthridine derivative **7**.

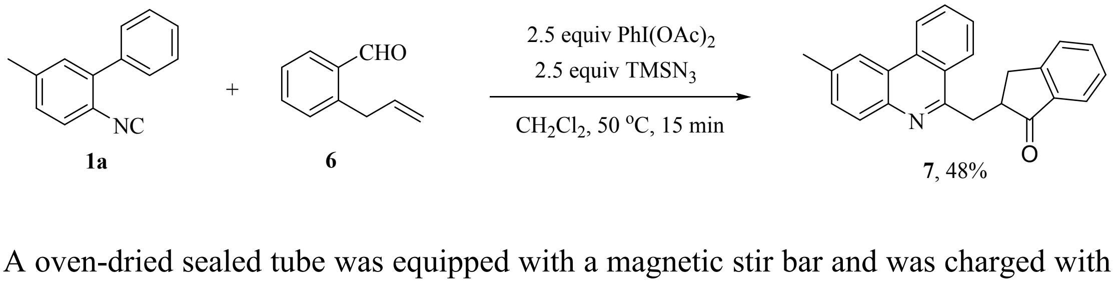

A oven-dried sealed tube was equipped with a magnetic stir bar and was charged with a mixture of 2-isocyanobiphenyl **1a** (0.2 mmol), aldehyde **6** (1.2 mmol) and $TMSN_3$ (0.5 mmol) in CH_2Cl_2 (1.0 mL). Then (diacetoxyiodo)benzene (PIDA) (0.5 mmol)

was dissolved in CH_2Cl_2 (1.0 mL) and added dropwise to the reaction mixture for 10 minutes at 50 °C. After the reaction was complete (as determined by TLC analysis), the reaction was cooled to room temperature and CH_2Cl_2 (20 mL) was added to the solution and washed with water (20 ml x 3), dried over Na_2SO_4, concentrated under reduced pressure. The crude product was purified by column chromatography on silica gel (petroleum ether/EtOAc = 30:1) to afford the targeted product **7**.

4.2 Product characterization

(2-methylphenanthridin-6-yl)(phenyl)methanone (3a) [4]

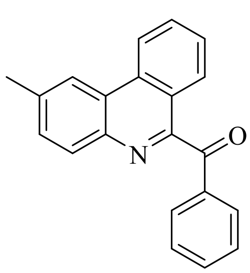

Yield: 74%. Mp 171-173 °C. Pale yellow solid. ^{1}H NMR (400 MHz, $CDCl_3$): δ 8.70 (d, J = 8.4 Hz, 1H), 8.44 (s, 1H), 8.16-8.09 (m, 2H), 8.04 (d, J = 8.0 Hz, 2H), 7.88 (t, J = 7.2 Hz, 1H), 7.67-7.60 (m, 3H), 7.48 (t, J = 7.2 Hz, 2H), 2.68 (s, 3H); ^{13}C NMR (125 MHz, $CDCl_3$): δ 194.9, 156.4, 140.9, 138.3, 136.3, 133.8, 133.0, 131.0, 130.8, 130.4, 128.5, 127.6, 127.2, 124.3, 124.0, 122.2, 121.7, 22.1; HRMS (ESI): Exact mass calcd for $C_{21}H_{15}NO$ $[M+H]^+$, 298.1193; Found: 298.1190.

(2-methylphenanthridin-6-yl)(p-tolyl)methanone (3b) [4a]

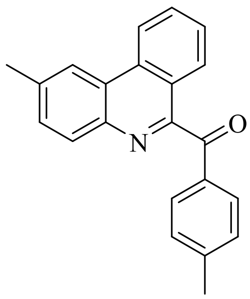

Yield: 66%. Mp 170-172 °C. Yellow solid. ^{1}H NMR (400 MHz, $CDCl_3$): δ 8.70 (d, J = 8.4 Hz, 1H), 8.43 (s, 1H), 8.11 (t, J = 7.2 Hz, 2H), 7.93 (d, J = 8.0 Hz, 2H), 7.87 (t, J = 7.6 Hz, 1H), 7.66-7.60 (m, 2H), 7.27 (d, J = 7.6 Hz, 2H), 2.67 (s, 3H), 2.43 (s, 3H); ^{13}C NMR (125 MHz, $CDCl_3$): δ 194.6, 156.8, 144.9, 141.0, 138.2, 133.9, 133.0, 130.9, 129.3, 127.6, 127.3, 124.3, 123.9, 122.2, 121.7, 22.1, 21.8; HRMS (ESI): Exact mass calcd for $C_{22}H_{17}NO$ $[M+H]^+$, 312.1383; Found: 312.1385.

(4-ethylphenyl)(2-methylphenanthridin-6-yl)methanone (3c)

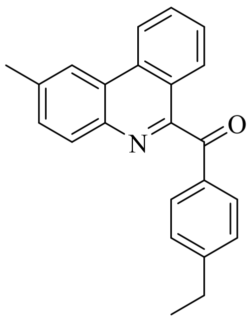

Yield: 63%. Mp 129-131 °C. Yellow solid. ^1H NMR (400 MHz, $CDCl_3$): δ 8.70 (d, *J* = 8.0 Hz, 1H), 8.43 (s, 1H), 8.12 (t, *J* = 8.0 Hz, 2H), 7.96 (d, *J* = 7.6 Hz, 2H), 7.87 (t, *J* = 7.6 Hz, 1H), 7.66-7.60 (m, 2H), 7.28 (d, *J* = 7.6 Hz, 2H), 2.75-2.69 (m, 2H), 2.67 (s, 3H), 1.26 (t, *J* = 7.2 Hz, 3H); ^{13}C NMR (125 MHz, $CDCl_3$): δ 197.5, 157.2, 141.3, 140.2, 138.3, 137.0, 133.2, 132.4, 132.0, 131.8, 130.7, 130.6, 127.6, 127.4, 125.4, 124.5, 124.1, 122.2, 121.6, 29.1, 22.1, 15.1; HRMS (ESI): Exact mass calcd for $C_{23}H_{19}NO$ $[M+H]^+$, 326.1539; Found: 326.1539.

(4-methoxyphenyl)(2-methylphenanthridin-6-yl)methanone (3d) [4a]

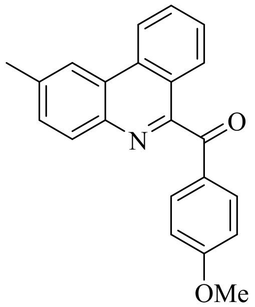

Yield: 76%. Mp 142-144 °C. White solid. ^1H NMR (400 MHz, $CDCl_3$): δ 8.70 (d, *J* = 8.0 Hz, 1H), 8.43 (s, 1H), 8.12 (t, *J* = 5.6 Hz, 2H), 8.01 (t, *J* = 7.6 Hz, 2H), 7.87 (t, *J* = 7.6 Hz, 1H), 7.65-7.60 (m, 2H), 6.94 (d, *J* = 7.6 Hz, 2H), 3.87 (s, 3H), 2.67 (s, 3H); ^{13}C NMR (125 MHz, $CDCl_3$): δ 193.6, 164.4, 157.1, 141.1, 138.2, 133.3, 133.1, 131.0, 130.9, 130.4, 129.5, 127.6, 127.5, 124.4, 124.0, 122.3, 121.8, 113.9, 55.6, 22.2; HRMS (ESI): Exact mass calcd for $C_{22}H_{17}NO_2$ $[M+H]^+$, 328.1332; Found: 328.1331.

(4-fluorophenyl)(2-methylphenanthridin-6-yl)methanone (3e) [4a]

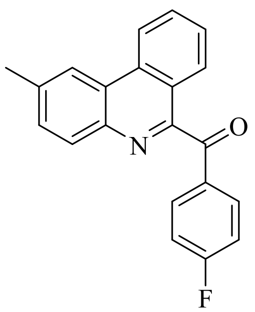

Yield: 49%. Mp 188-190 °C. White solid. ^1H NMR (300 MHz, $CDCl_3$): δ 8.71 (d, *J* =

8.1 Hz, 1H), 8.44 (s, 1H), 8.18 (d, *J* = 8.4 Hz, 1H), 8.14-8.09 (m, 3H), 7.92-7.87 (m, 1H), 7.70-7.62 (m, 2H), 7.17 (t, *J* = 8.7 Hz, 2H), 2.69 (s, 3H); ^{13}C NMR (75 MHz, $CDCl_3$): δ 193.2, 168.0, 164.6, 155.9, 140.9, 138.6, 133.7, 133.6, 133.1, 132.8, 132.7, 131.1, 130.9, 130.4, 127.7, 127.2, 124.4, 123.9, 122.3, 121.8, 115.9, 115.6, 22.2; HRMS (ESI): Exact mass calcd for $C_{21}H_{14}FNO$ $[M+H]^+$, 316.1132; Found: 316.1129.

(4-chlorophenyl)(2-methylphenanthridin-6-yl)methanone (3f) [4a]

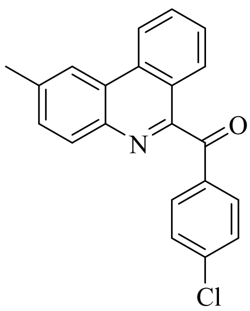

Yield: 45%. Mp 216-218 °C. White solid. ^{1}H NMR (300 MHz, $CDCl_3$): δ 8.72 (d, *J* = 8.4 Hz, 1H), 8.45 (s, 1H), 8.20 (d, *J* = 8.1 Hz, 1H), 8.11 (d, *J* = 8.4 Hz, 1H), 8.04-8.00 (m, 2H), 7.93-7.88 (m, 1H), 7.71-7.62 (m, 2H), 7.49-7.45 (m, 2H), 2.69 (s, 3H); ^{13}C NMR (75 MHz, $CDCl_3$): δ 193.5, 155.6, 140.8, 140.4, 138.7, 134.7, 133.1, 132.3, 131.1, 131.0, 130.4, 128.9, 127.8, 127.2, 124.5, 123.9, 122.3, 121.8, 22.2.

(2-methylphenanthridin-6-yl)(4-(trifluoromethyl)phenyl)methanone (3g)

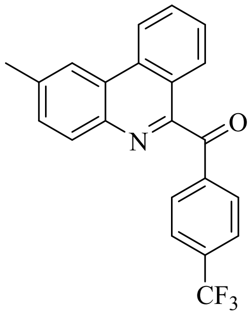

Yield: 13%. Pale yellow oil. ^{1}H NMR (300 MHz, $CDCl_3$): δ 8.75 (d, *J* = 8.4 Hz, 1H), 8.47 (s, 1H), 8.28 (d, *J* = 8.4 Hz, 1H), 8.20 (d, *J* = 8.1 Hz, 2H), 8.10 (d, *J* = 8.4 Hz, 1H), 7.96-7.91 (m, 1H), 7.79-7.63 (m, 4H), 2.71 (s, 3H); ^{13}C NMR (75 MHz, $CDCl_3$): δ 193.5, 154.9, 140.8, 139.3, 139.0, 133.2, 131.2, 131.0, 130.5, 129.2, 127.9, 127.1, 125.5, 125.4, 124.6, 123.9, 122.4, 121.8, 22.2; HRMS (ESI): Exact mass calcd for $C_{22}H_{14}F_3NO$ $[M+H]^+$, 366.1025; Found: 366.1029.

(2-methylphenanthridin-6-yl)(o-tolyl)methanone (3h) [4a]

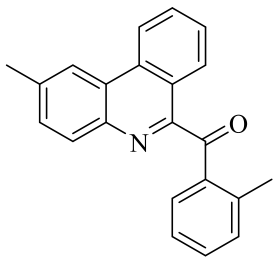

Yield: 50%. Mp 126-128 °C. White solid. ^{1}H NMR (400 MHz, $CDCl_3$): δ 8.70 (d, *J* = 6.8 Hz, 1H), 8.42 (s, 1H), 8.25 (d, *J* = 8.0 Hz, 1H), 8.05 (d, *J* = 8.4 Hz, 1H), 7.88 (t, *J* = 7.2 Hz, 1H), 7.67 (t, *J* = 8.0 Hz, 1H), 7.58 (d, *J* = 8.4 Hz, 1H), 7.52 (d, *J* = 7.6 Hz, 1H), 7.45 (t, *J* = 7.6 Hz, 1H), 7.35 (d, *J* = 7.2 Hz, 1H), 7.18 (t, *J* = 7.6 Hz, 1H), 2.66 (s, 6H); ^{13}C NMR (100 MHz, $CDCl_3$): δ 197.5, 157.2, 141.3, 140.2, 138.3, 137.0, 133.2, 132.4, 132.0, 131.8, 130.7, 130.6, 127.6, 127.4, 125.4, 124.5, 124.1, 122.2, 121.6, 21.9, 21.4; HRMS (ESI): Exact mass calcd for $C_{22}H_{17}NO$ $[M+H]^+$, 312.1385; Found: 312.1384.

(2-(*tert*-butyl)phenyl)(2-methylphenanthridin-6-yl)methanone (3i)

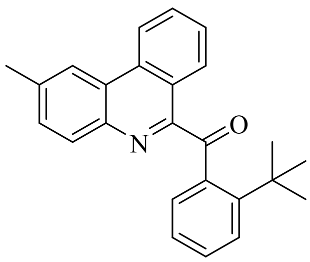

Yield: 35%. Pale yellow oil. ^{1}H NMR (300 MHz, $CDCl_3$): δ 9.04 (d, *J* = 8.4 Hz, 1H), 8.72 (d, *J* = 8.4 Hz, 1H), 8.41 (s, 1H), 7.99-7.77 (m, 3H), 7.66 (d, *J* = 8.1 Hz, 1H), 7.56-7.53 (m, 1H), 7.47-7.42 (m, 1H), 7.20 (d, *J* = 4.5 Hz, 2H), 2.66 (s, 3H), 1.50 (s, 9H); ^{13}C NMR (100 MHz, $CDCl_3$): δ 201.5, 154.3, 149.1, 140.9, 140.8, 139.3, 133.2, 131.2, 130.6, 129.4, 128.9, 128.1, 127.6, 127.5, 124.9, 124.6, 124.5, 122.1, 121.6, 36.4, 32.3, 22.2; HRMS (ESI): Exact mass calcd for $C_{25}H_{23}NO$ $[M+H]^+$, 354.1852; Found: 354.1859.

(2,6-dimethylphenyl)(2-methylphenanthridin-6-yl)methanone (3j)

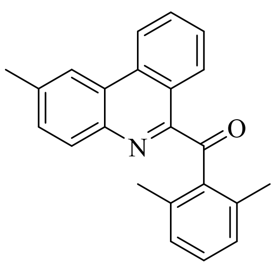

Yield: 51%. Mp 101-103 °C. White solid. ^{1}H NMR (400 MHz, $CDCl_3$): δ 9.05 (d, *J* = 8.4 Hz, 1H), 8.68 (d, *J* = 8.4 Hz, 1H), 8.36 (s, 1H), 7.93 (d, *J* = 8.4 Hz, 1H), 7.87 (t, *J*

= 7.6 Hz, 1H), 7.77 (t, J = 7.6 Hz, 1H), 7.50 (d, J = 8.4 Hz, 1H), 7.23 (d, J = 7.2 Hz, 1H), 7.07 (d, J = 7.6 Hz, 2H), 2.62 (s, 3H), 2.22 (s, 6H); ^{13}C NMR (100 MHz, $CDCl_3$): δ 202.4, 153.8, 142.2, 141.5, 139.6, 135.2, 133.7, 131.6, 130.7, 128.9, 128.3, 127.9, 127.7, 125.4, 124.0, 122.4, 121.8, 22.3, 20.0; HRMS (ESI): Exact mass calcd for $C_{23}H_{19}NO$ $[M+H]^+$, 326.1539; Found: 326.1540.

(2-methylphenanthridin-6-yl)(m-tolyl)methanone (3k) [4a]

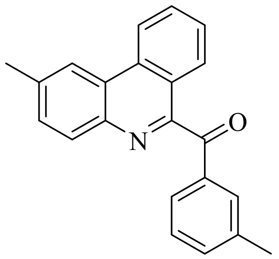

Yield: 42%. Mp 124-126 °C. Yellow solid. ^{1}H NMR (400 MHz, $CDCl_3$): δ 8.71 (d, J = 8.4 Hz, 1H), 8.44 (s, 1H), 8.12 (d, J = 8.4 Hz, 2H), 7.87 (t, J = 7.6 Hz, 2H), 7.80 (t, J = 7.6 Hz, 1H), 7.66-7.64 (m, 2H), 7.43 (d, J = 7.2 Hz, 1H), 7.35 (t, J = 7.6 Hz, 1H), 2.68 (s, 3H), 2.38 (s, 3H); ^{13}C NMR (125 MHz, $CDCl_3$): δ 195.2, 156.8, 141.0, 138.4, 138.3, 136.4, 134.8, 133.0, 131.1, 131.0, 130.8, 130.4, 128.5, 127.6, 127.3, 124.3, 124.0, 122.3, 121.8, 22.1, 21.3; HRMS (ESI): Exact mass calcd for $C_{22}H_{17}NO$ $[M+H]^+$, 312.1383; Found: 312.1384.

(3-methoxyphenyl)(2-methylphenanthridin-6-yl)methanone (3l)

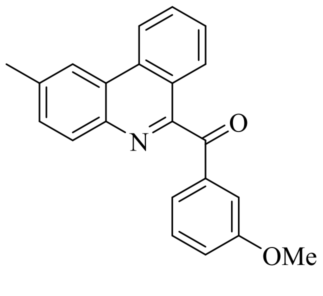

Yield: 52%. Mp 148-150 °C. White solid. ^{1}H NMR (400 MHz, $CDCl_3$): δ 8.70 (d, J = 8.0 Hz, 1H), 8.43 (s, 1H), 8.12 (t, J = 8.0 Hz, 2H), 7.87 (t, J = 7.2 Hz, 1H), 7.67-7.60 (m, 3H), 7.50 (d, J = 7.6 Hz, 1H), 7.34 (t, J = 8.0 Hz, 1H), 7.17(d, J = 8.0 Hz, 1H), 3.85 (s, 3H), 2.67 (s, 3H); ^{13}C NMR (100 MHz, $CDCl_3$): δ 194.7, 160.2, 156.8, 141.5, 138.5, 138.3, 133.4, 131.1, 130.8, 129.7, 127.8, 127.6, 124.7, 124.3, 124.2, 122.5, 120.8, 114.9, 55.7, 22.2; HRMS (ESI): Exact mass calcd for $C_{22}H_{17}NO_2$ $[M+H]^+$, 328.1332; Found: 328.1332.

(8-(tert-butyl)-2-methylphenanthridin-6-yl)(4-methoxyphenyl)methanone (3m)

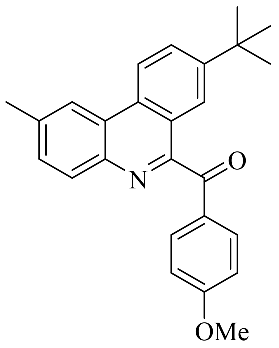

Yield: 47%. Mp 128-130 °C. Yellow solid. ^{1}H NMR (400 MHz, $CDCl_3$): δ 8.62 (d, J = 8.4 Hz, 1H), 8.39 (s, 1H), 8.08 (d, J = 8.8 Hz, 2H), 8.03 (d, J = 7.6 Hz, 2H), 7.94 (d, J = 8.4 Hz, 1H), 7.57 (d, J = 8.4 Hz, 1H), 6.95 (d, J = 8.0 Hz, 2H), 3.87 (s, 3H), 2.66 (s, 3H), 1.38 (s, 9H); ^{13}C NMR (125 MHz, $CDCl_3$): δ 193.8, 164.2, 156.9, 150.7, 140.8, 138.0, 133.2, 130.9, 130.4, 130.2, 129.6, 129.4, 124.3, 124.0, 122.8, 122.0, 121.6, 113.8, 55.5, 35.0, 31.1, 22.1; HRMS (ESI): Exact mass calcd for $C_{26}H_{25}NO_2$ $[M+H]^+$, 384.1958; Found: 384.1960.

(8-methoxy-2-methylphenanthridin-6-yl)(4-methoxyphenyl)methanone (3n)

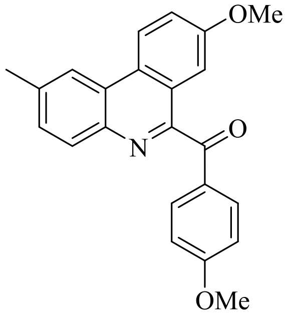

Yield: 62%. Mp 185-187 °C. Yellow solid. ^{1}H NMR (400 MHz, $CDCl_3$): δ 8.59 (d, J = 8.8 Hz, 1H), 8.33 (s, 1H), 8.08-8.01 (m, 3H), 7.55-7.48 (m, 3H), 6.95 (d, J = 8.0 Hz, 2H), 3.88 (s, 6H), 2.65 (s, 3H); ^{13}C NMR (125 MHz, $CDCl_3$): δ 193.6, 164.2, 158.8, 155.6, 140.2, 138.3, 133.3, 132.2, 130.2, 129.8, 129.4, 127.5, 125.3, 124.5, 123.8, 122.2, 121.2, 113.8, 106.7 55.5, 22.1; HRMS (ESI): Exact mass calcd for $C_{23}H_{19}NO_3$ $[M+H]^+$, 358.1438; Found: 358.1436.

(8-(benzyloxy)-2-methylphenanthridin-6-yl)(4-methoxyphenyl)methanone (3o)

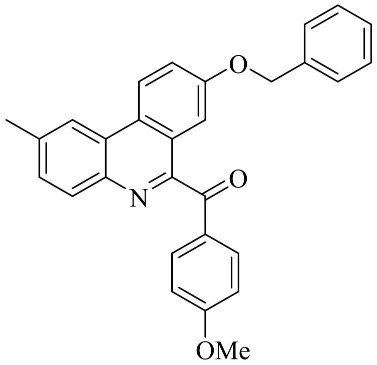

Yield: 76%. Mp 189-191 °C. White solid. ^{1}H NMR (400 MHz, $CDCl_3$): δ 8.61 (d, *J* = 8.8 Hz, 1H), 8.34 (s, 1H), 8.07 (d, *J* = 8.4 Hz, 1H), 8.01 (d, *J* = 7.6 Hz, 2H), 7.61 (s, 1H), 7.55 (t, *J* = 8.4 Hz, 2H), 7.43 (d, *J* = 7.2 Hz, 2H), 7.38-7.31 (m, 3H), 6.95 (d, *J* = 7.6 Hz, 2H), 5.12 (s, 2H), 3.89 (s, 3H), 2.65 (s, 3H); ^{13}C NMR (100 MHz, $CDCl_3$): δ 193.7, 164.5, 158.3, 156.1, 140.8, 138.4, 136.7, 133.4, 130.7, 130.1, 130.0, 128.8, 128.3, 128.0, 127.9, 125.6, 124.8, 124.1, 122.6, 121.4, 114.1, 108.9, 70.8, 55.7, 22.2; HRMS (ESI): Exact mass calcd for $C_{29}H_{23}NO_3$ $[M+H]^+$, 434.1751; Found: 434.1752.

(8-fluoro-2-methylphenanthridin-6-yl)(4-methoxyphenyl)methanone (3p)

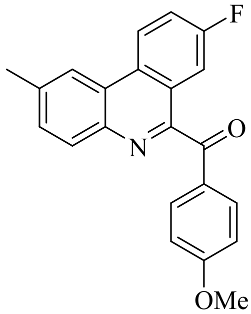

Yield: 87%. Mp 176-178 °C. Yellow solid. ^{1}H NMR (400 MHz, $CDCl_3$): δ 8.70-8.67 (m, 1H), 8.36 (s, 1H), 8.10 (d, *J* = 8.4 Hz, 1H), 8.03 (d, *J* = 8.0 Hz, 2H), 7.83 (d, *J* = 9.6 Hz, 1H), 7.61 (t, *J* = 8.0 Hz, 2H), 6.96 (d, *J* = 7.6 Hz, 2H), 3.89 (s, 3H), 2.67 (s, 3H); ^{13}C NMR (100 MHz, $CDCl_3$): δ 192.6, 164.4, 162.8, 160.3, 155.7, 140.8, 138.7, 133.2, 130.6, 130.5, 129.8, 129.6, 125.2, 124.7, 124.6, 124.1, 121.4, 120.2, 119.9, 113.9, 112.1, 111.9, 55.4, 21.9; HRMS (ESI): Exact mass calcd for $C_{22}H_{16}FNO_2$ $[M+H]^+$, 346.1238; Found: 346.1240.

(4-methoxyphenyl)(2-methyl-8-(trifluoromethyl)phenanthridin-6-yl)methanone (3q)

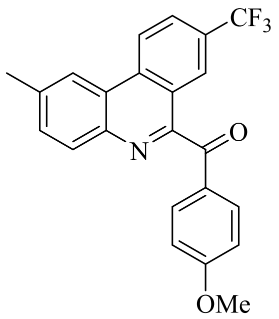

Yield: 62%. Mp 201-203 °C. White solid. ^{1}H NMR (400 MHz, $CDCl_3$): δ 8.81 (d, *J* = 8.8 Hz, 1H), 8.49 (s, 1H), 8.44 (s, 1H), 8.14 (d, *J* = 8.4 Hz, 1H), 8.05 (d, *J* = 8.4 Hz, 3H), 7.69 (t, *J* = 8.4 Hz, 1H), 6.97 (t, *J* = 7.6 Hz, 2H), 3.89 (s, 3H), 2.69 (s, 3H); ^{13}C NMR (125 MHz, $CDCl_3$): δ 192.6, 164.6, 156.3, 141.6, 139.2, 133.5, 132.1, 130.7, 129.2, 126.8, 126.7,125.1, 125.0, 123.5, 123.4, 122.2, 114.0, 55.7, 22.2; HRMS (ESI): Exact mass calcd for $C_{23}H_{16}F_3NO_2$ $[M+H]^+$, 396.1206; Found: 396.1206.

(2,7-dimethylphenanthridin-6-yl)(4-methoxyphenyl)methanone (3r) and (2,9-dimethyl- phenanthridin-6-yl)(4-methoxyphenyl)methanone (3r')

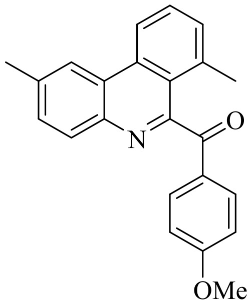

3r

+

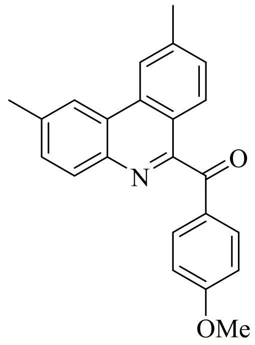

3r'

Yield: **3r** = 36%, **3r'** = 20%. Yellow solid. ^{1}H NMR (400 MHz, $CDCl_3$): δ 8.61 (d, *J* = 8.4 Hz, 0.64H), 8.45 (s, 0.36H), 8.42 (s, 1H), 8.09-7.94 (m, 3H), 7.75 (t, *J* = 7.2 Hz, 0.64H), 7.58 (d, *J* = 6.0 Hz, 1H), 7.46 (d, *J* = 7.2 Hz, 1H), 6.94 (t, *J* = 7.2 Hz, 2H), 3.87 (s, 3H), 2.65 (s, 4H), 2.54 (s, 1.8H); ^{13}C NMR (100 MHz, $CDCl_3$): δ 195.2, 164.8, 164.7, 157.7, 141.3, 138.2, 136.7, 134.7, 133.6, 133.3, 131.1, 131.0, 130.9, 130.5, 130.3, 129.7, 127.8, 124.9, 122.4, 122.3, 122.1, 120.9, 114.5, 114.4, 55.9, 32.0, 24.1, 23.0, 22.4, 22.3.

(4-methoxyphenyl)(10-methylphenanthridin-6-yl)methanone (3s)

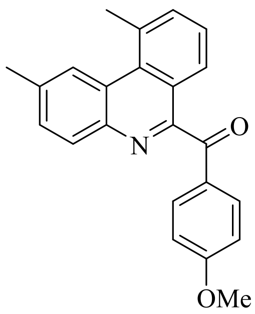

Yield: 44%. Mp 143-145 °C. Yellow solid. ^{1}H NMR (400 MHz, $CDCl_3$): δ 8.68 (s, 1H), 8.14 (d, *J* = 8.4 Hz, 1H), 7.95 (t, *J* = 7.2 Hz, 3H), 7.69 (d, *J* = 7.2 Hz, 1H), 7.61 (d, *J* = 8.4 Hz, 1H), 7.51 (t, *J* = 7.6 Hz, 1H), 6.92 (d, *J* = 7.6 Hz, 2H), 3.86 (s, 3H), 3.18 (s, 3H), 2.67 (s, 3H); ^{13}C NMR (100 MHz, $CDCl_3$): δ 193.9, 164.3, 158.1, 142.6, 136.9, 135.5, 135.0, 133.0, 130.7, 129.8, 129.7, 126.9, 126.4, 126.0, 125.8, 125.5, 113.9, 55.4, 26.7, 22.3; HRMS (ESI): Exact mass calcd for $C_{22}H_{17}NO_2$ $[M+H]^+$, 342.1489; Found: 342.1487.

(4-methoxyphenyl)(phenanthridin-6-yl)methanone (3t)

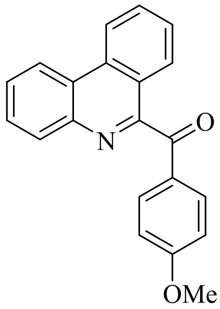

Yield: 66%. Mp 162-164 °C. Yellow solid. ^{1}H NMR (400 MHz, $CDCl_3$): δ 8.72 (d, *J* = 8.0 Hz, 1H), 8.65 (d, *J* = 7.6 Hz, 1H), 8.22 (t, *J* = 7.6 Hz, 1H), 8.12(d, *J* = 8.0 Hz, 1H), 8.02 (d, *J* = 8.0 Hz, 2H), 7.89 (t, *J* = 7.6 Hz, 1H), 7.81-7.74 (m, 2H), 7.66 (t, *J* = 7.2 Hz, 1H), 6.94 (d, *J* = 8.4 Hz, 2H), 3.87 (s, 3H); ^{13}C NMR (125 MHz, $CDCl_3$): δ 193.4, 164.3, 158.0, 142.7, 133.3, 133.2, 131.2, 130.6, 129.2, 129.0, 128.0, 127.7, 127.4, 124.4, 123.8, 122.2, 122.1, 113.9, 55.5; HRMS (ESI): Exact mass calcd for $C_{21}H_{15}NO_2$ $[M+H]^+$, 314.1176; Found: 314.1179.

(4-methoxyphenyl)(3-methylphenanthridin-6-yl)methanone (3u)

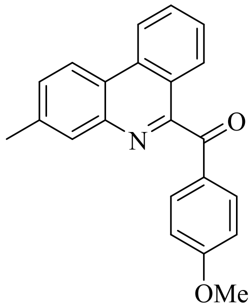

Yield: 62%. Mp 143-145 °C. Yellow solid. ^{1}H NMR (400 MHz, $CDCl_3$): δ 8.66 (d, *J*

= 8.4 Hz, 1H), 8.53 (d, *J* = 8.4 Hz, 1H), 8.10 (d, *J* = 8.4 Hz, 1H), 8.01 (d, *J* = 7.6 Hz, 3H), 7.86 (t, *J* = 7.6 Hz, 1H), 7.63-7.57 (m, 2H), 6.94 (d, *J* = 8.0 Hz, 2H), 3.87 (s, 3H), 2.60 (s, 3H); ^{13}C NMR (125 MHz, $CDCl_3$): δ 193.5, 164.3, 157.9, 142.8, 139.3, 133.3, 133.2, 131.1, 130.1, 129.7, 129.3, 127.4, 127.2, 123.5, 122.1, 122.0, 121.9, 113.8, 55.5, 21.5; HRMS (ESI): Exact mass calcd for $C_{22}H_{17}NO_2$ $[M+H]^+$, 328.1332; Found: 328.1334.

(3-chlorophenanthridin-6-yl)(4-methoxyphenyl)methanone (3v)

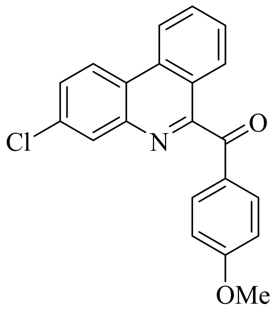

Yield: 75%. Mp 162-164 °C. Yellow solid. ^{1}H NMR (400 MHz, $CDCl_3$): δ 8.65 (d, *J* = 8.4 Hz, 1H), 8.56 (d, *J* = 8.8 Hz, 1H), 8.22 (s, 1H), 8.12 (d, *J* = 8.4 Hz, 1H), 8.00 (d, *J* = 7.6 Hz, 2H), 7.90 (t, *J* = 7.2 Hz, 1H), 7.72-7.65 (m, 2H), 6.95 (d, *J* = 7.6 Hz, 2H), 3.88 (s, 3H); ^{13}C NMR (100 MHz, $CDCl_3$): δ 193.0, 164.8, 159.4, 143.9, 135.1, 133.3, 131.7, 130.1, 129.7, 128.7, 128.1, 128.0, 124.2, 123.7, 123.2, 122.4, 114.2, 55.7; HRMS (ESI): Exact mass calcd for $C_{21}H_{14}ClNO_2$ $[M+H]^+$, 348.0784; Found: 348.0786.

(4-methoxyphenyl)(2-(trifluoromethyl)phenanthridin-6-yl)methanone (3w)

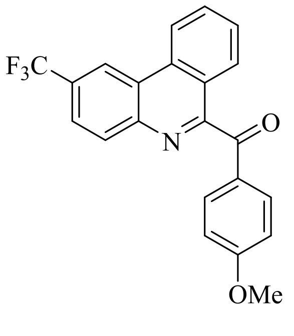

Yield: 81%. Mp 184-186 °C. White solid. ^{1}H NMR (400 MHz, $CDCl_3$): δ 8.92 (s, 1H), 8.75 (d, *J* = 8.4 Hz, 1H), 8.33 (d, *J* = 8.4 Hz, 1H), 8.15 (d, *J* = 8.0 Hz, 1H), 8.00-7.95 (m, 4H), 7.73 (t, *J* = 7.2 Hz, 1H), 6.96 (d, *J* = 8.0 Hz, 2H), 3.88 (s, 3H); ^{13}C NMR (125 MHz, $CDCl_3$): δ 192.9, 164.6, 160.2, 144.2, 133.1, 133.0, 131.9, 131.5, 128.8, 127.8, 125.0, 124.0, 122.3, 120.0, 119.9, 114.0, 55.6; HRMS (ESI): Exact mass calcd for $C_{22}H_{14}F_3NO_2$ $[M+H]^+$, 382.1049; Found: 382.1050.

methyl 1-(4-methoxybenzoyl)-4-phenylisoquinoline-3-carboxylate (5a)

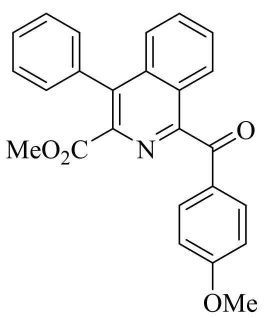

Yield: 48%. Mp 165-167 ℃. White solid. ^{1}H NMR (300 MHz, $CDCl_3$): δ 8.24-8.20 (m, 1H), 8.10-8.04 (m, 2H), 7.76-7.66 (m, 3H), 7.55 (t, J = 5.7 Hz, 3H), 7.46-7.41 (m, 2H), 7.02-6.97 (m, 2H), 3.90 (s, 3H), 3.69 (s, 3H); ^{13}C NMR (75 MHz, $CDCl_3$): δ 192.5, 167.2, 164.4, 156.5, 140.6, 136.4, 135.6, 134.9, 133.4, 131.2, 129.7, 129.3, 129.2, 128.3, 126.8, 126.5, 126.4, 113.9, 55.6, 52.5; HRMS (ESI): Exact mass calcd for $C_{25}H_{19}NO_4$ $[M+H]^+$, 398.1392; Found: 398.1394.

methyl 1-(4-methoxybenzoyl)-7-methyl-4-(p-tolyl)isoquinoline-3-carboxylate (5b)

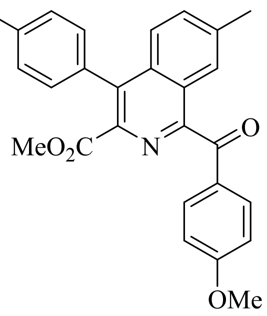

Yield: 46%. Mp 132-134 ℃. Pale yellow Solid. ^{1}H NMR (300 MHz, $CDCl_3$): δ 8.07-8.03 (m, 2H), 7.97 (s, 1H), 7.66 (d, J = 8.7 Hz, 1H), 7.51 (d, J = 8.7 Hz, 1H), 7.36-7.28 (m, 4H), 6.98 (t, J = 8.7 Hz, 2H), 3.88 (s, 3H), 3.71 (s, 3H), 2.49 (s, 6H); ^{13}C NMR (75 MHz, $CDCl_3$): δ 192.7, 167.3, 164.3, 155.5, 139.8, 139.6, 137.9, 135.1, 134.8, 133.4, 132.7, 129.5, 129.3, 129.0, 126.8, 126.7, 125.1, 113.9, 55.6, 52.4, 21.9, 21.4; HRMS (ESI): Exact mass calcd for $C_{27}H_{23}NO_4$ $[M+H]^+$, 426.1705; Found: 426.1715.

Methyl 7-fluoro-4-(4-fluorophenyl)-1-(4-methoxybenzoyl)isoquinoline-3-carboxylate (5c)

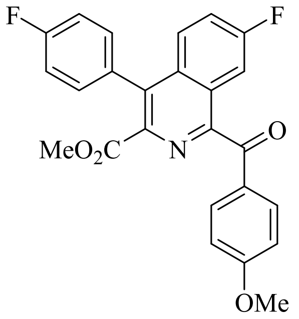

Yield: 42%. Mp 148-150 ℃. White solid. ^{1}H NMR (300 MHz, $CDCl_3$): δ 8.09-8.05

(m, 2H), 7.92 (d, J = 9.6 Hz, 1H), 7.72 (t, J = 5.4 Hz, 1H), 7.52-7.45 (m, 1H), 7.41-7.36 (m, 2H), 7.27-7.22 (m, 2H), 7.00 (d, J = 5.1 Hz, 2H), 3.90 (s, 3H), 3.71 (s, 3H); ^{13}C NMR (75 MHz, $CDCl_3$): δ 191.7, 166.8, 164.5, 163.8, 161.2, 160.5, 155.5, 140.2, 140.1, 134.2, 133.7, 133.5, 131.4, 131.3, 131.1, 129.7, 129.6, 128.9, 127.9, 127.7, 122.1, 121.7, 115.8, 115.5, 113.9, 110.5, 110.2, 55.6, 52.6; HRMS (ESI): Exact mass calcd for $C_{25}H_{17}F_2NO_4$ $[M+H]^+$, 434.1204; Found: 434.1205.

methyl 7-chloro-4-(4-chlorophenyl)-1-(4-methoxybenzoyl)isoquinoline-3-carboxylate(5d)

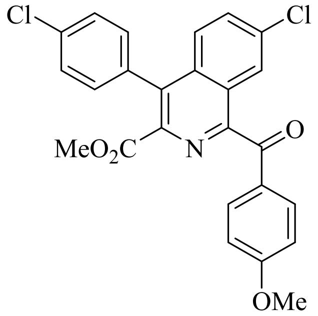

Yield: 61%. Mp 137-138 °C. Pale yellow solid. ^{1}H NMR (300 MHz, $CDCl_3$): δ 8.27 (s, 1H), 8.04 (t, J = 3.0 Hz, 2H), 7.64 (t, J = 3.3 Hz, 2H), 7.56-7.52 (m, 2H), 7.36-7.32 (m, 2H), 7.02-6.98 (m, 2H), 3.92 (s, 3H), 3.73 (s, 3H); ^{13}C NMR (75 MHz, $CDCl_3$): δ 191.6, 166.6, 164.5, 155.6, 140.5, 135.8, 134.8, 134.7, 133.9, 133.5, 132.4, 130.9, 128.9, 128.8, 128.3, 127.2, 125.5, 114.0, 55.6, 52.7; HRMS (ESI): Exact mass calcd for $C_{25}H_{17}Cl_2NO_4$ $[M+H]^+$, 466.0613, Found: 466.0618; $[M+2+H]^+$, 468.0305, Found: 468.0309; $[M+4+H]^+$, 470.0125, Found: 470.0122.

methyl 1-(4-methoxybenzoyl)-4-methylisoquinoline-3-carboxylate (5e)

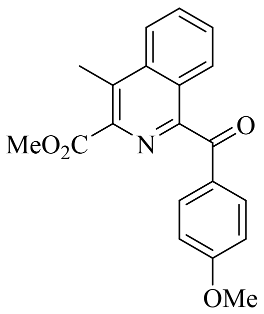

Yield: 41%. Mp 130-132 °C. Pale yellow solid. ^{1}H NMR (300 MHz, $CDCl_3$): δ 8.25-8.14 (m, 2H), 8.00-7.95 (m, 2H), 7.86-7.80 (m, 1H), 7.70-7.64 (m, 1H), 6.97-6.92 (m, 2H), 3.98 (s, 3H), 3.88 (s, 3H), 2.93 (s, 3H); ^{13}C NMR (75 MHz, $CDCl_3$): δ 192.6, 167.7, 164.2, 155.0, 140.3, 136.6, 133.4, 131.1, 130.9, 129.4, 129.0, 126.9, 126.3, 124.5, 113.8, 55.5, 52.7, 14.6; HRMS (ESI): Exact mass calcd for $C_{20}H_{17}NO_4$ $[M+H]^+$, 336.1236; Found: 336.1233.

2-((2-methylphenanthridin-6-yl)methyl)-2,3-dihydro-1H-inden-1-one (7)

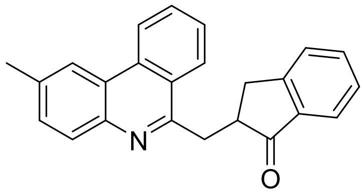

Yield: 48%. mp 172-174 °C. yellow solid. ^{1}H NMR (400 MHz, $CDCl_3$): δ 8.71 (d, *J* = 8.0 Hz, 1H), 8.42 (s, 1H), 8.29 (d, *J* = 8.4 Hz, 1H), 8.03 (d, *J* = 8.4 Hz, 1H), 7.88 (d, *J* = 7.2 Hz, 1H), 7.69 (t, *J* = 7.2 Hz, 1H), 7.58 (d, *J* = 8.4 Hz, 1H), 7.46 (t, *J* = 8.8 Hz, 2H), 7.39 (d, *J* = 7.6 Hz, 1H), 7.20 (t, *J* = 7.2 Hz, 1H), 3.34 (t, *J* =6.8 Hz, 2H), 3.11 (t, *J* = 7.2 Hz, 2H), 2.66 (s, 3H), 2.10-2.02 (m, 1H); ^{13}C NMR (125 MHz, $CDCl_3$): δ 197.6, 156.9, 143.3, 141.0, 138.5, 136.5, 133.0, 132.4, 131.3, 130.9, 130.8, 130.5, 127.8, 127.2, 125.9, 124.4, 123.9, 122.2, 121.7, 51.1, 31.5, 30.6, 22.1; HRMS (ESI): Exact mass calcd for $C_{24}H_{19}NO$ $[M+H]^+$, 338.1501; Found: 338.1502.

5. Free-Radical inhibition experiment

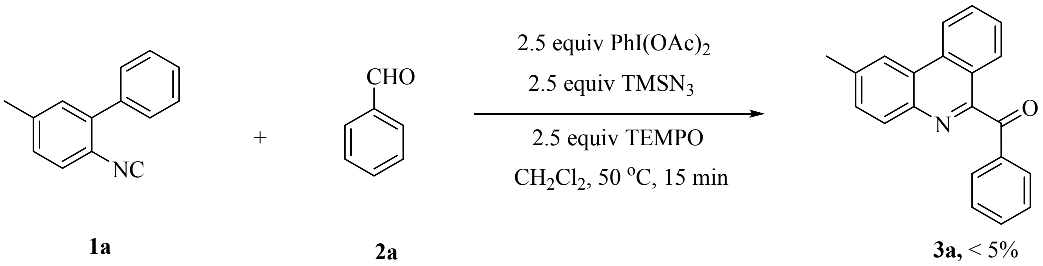

An oven-dried sealed tube was equipped with a magnetic stir bar and was charged with a mixture of 2-isocyanobiphenyl **1a** (0.2 mmol), benzaldehyde **2a** (1.2 mmol), TEMPO (0.5 mmol) and $TMSN_3$ (0.5 mmol) in CH_2Cl_2 (1.0 mL). Then (diacetoxyiodo)benzene (PIDA) (0.5 mmol) was dissolved in CH_2Cl_2 (1.0 mL) and added dropwise to the reaction mixture for 10 minutes at 50 °C. After stirring for 5 minutes, the reaction was monitored by TLC analyst and almost no the targeted product **3a** was observed along with a great material **1a** remained.

6. References

(1) (a) M. Tobisu, K. Koh, T. Furukawa, N. Chatani, *Angew. Chem., Int. Ed.,* 2012, **51**, 11363; (b) B. Zhang, C. Mück-Lichtenfeld, C. G. Daniliuc, A. Studer, *Angew. Chem., Int. Ed.,* 2013, **52**, 10792; (c) D. Leifert, C. G. Daniliuc, A. Studer, *Org. Lett.,* 2013, **15**, 6286; (d) X. Sun, S. Yu, *Org. Lett.,* 2014, **16**, 2938; (e) B. Zhang, C. G. Daniliuc, A. Studer, *Org. Lett.,* 2014, **16**, 250; (f) J. Liu, C. Fan, H. Yin, C. Qin, G. Zhang, X. Zhang, H. Yi, A. Lei, *Chem. Commun.,* 2014, **50**, 2145; (g) X. Li, M. Fang, P. Hu, G. Hong, Y. Tang, X. Xu, *Adv. Synth. Catal.,* 2014, **356**, 2103.

(2) (a) Y. Cheng, X. Yuan, H. Jiang, R. Wang, J. Ma, Y. Zhang, S. Yu, *Adv. Synth. Catal.,* 2014, **356**, 2859; (b) J. Li, Y. He, S. Luo, J. Lei, J. Wang, Z. Xie，Q. Zhu, *J. Org. Chem.,* 2015, **80**, 2223.

(3) A. R. Jagdale, J. H. Park, S. W. Youn, *J. Org. Chem.*, 2011, **76**, 7204.

(4) (a) D. Leifert, C. G. Daniliuc, A. Studer, *Org. Lett.,* 2013, **15**, 6286; (b) J. Liu, C. Fan, H. Yin, C. Qin, G. Zhang, X. Zhang, H. Yi, A. Lei, *Chem. Commun.,* 2014, **50**, 2145.

7. Copies of ^{1}H NMR and ^{13}C NMR spectra

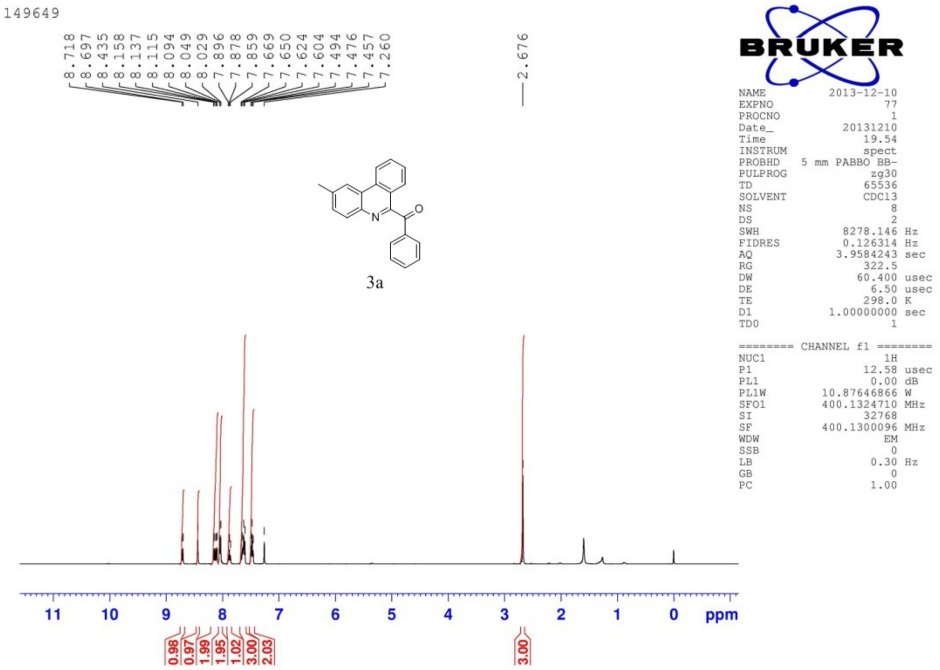

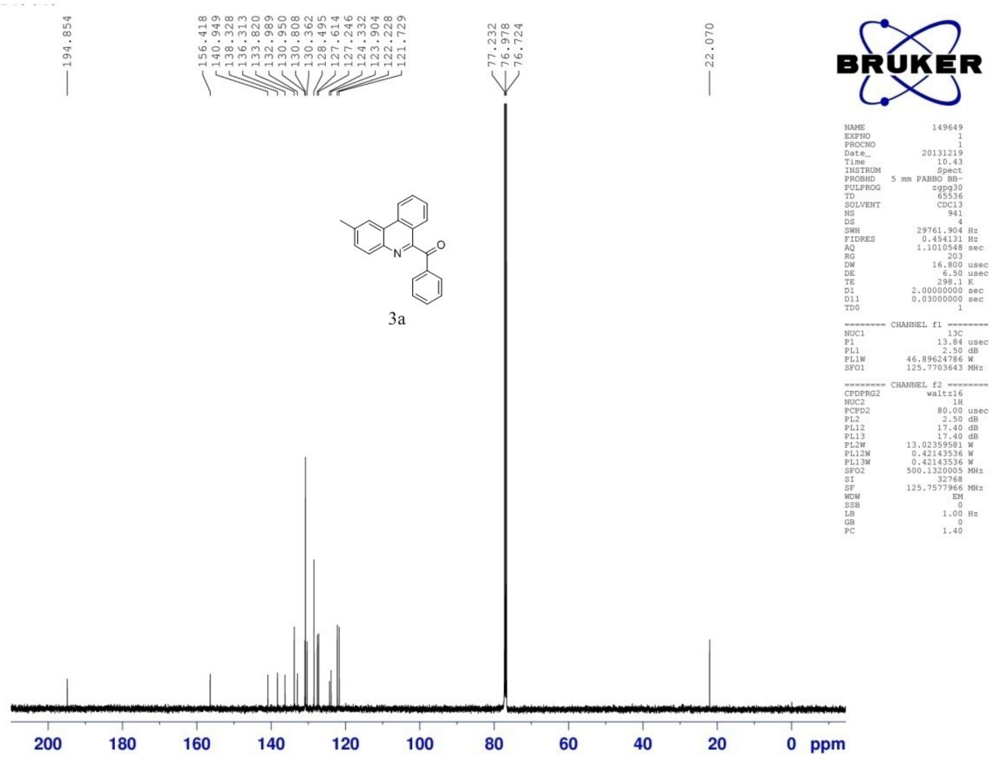

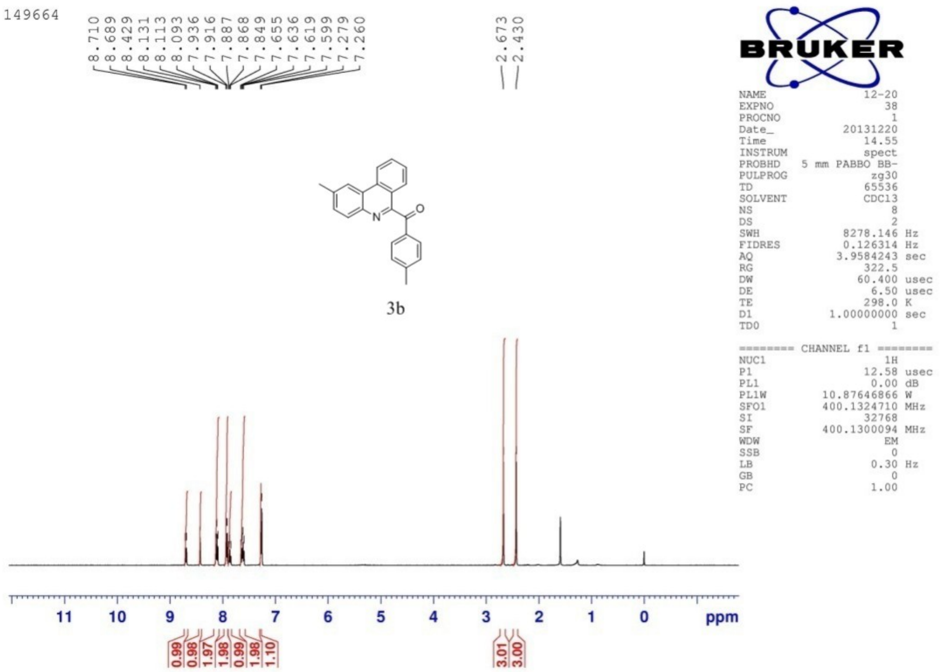
149664
8.710 8.689 8.429 8.131 8.113 8.093 7.936 7.916 7.887 7.868 7.849 7.655 7.636 7.619 7.599 7.279 7.260
2.673 2.430
N
O
3b
BRUKER
NAME 12-20
EXPNO 38
PROCNO 1
Date_ 20131220
Time 14.55
INSTRUM spect
PROBHD 5 mm PABBO BB-
PULPROG zg30
TD 65536
SOLVENT CDCl3
NS 8
DS 2
SWH 8278.146 Hz
FIDRES 0.126314 Hz
AQ 3.9584243 sec
RG 322.5
DW 60.400 usec
DE 6.50 usec
TE 298.0 K
D1 1.00000000 sec
TD0 1
======== CHANNEL f1 ========
NUC1 1H
P1 12.58 usec
PL1 0.00 dB
PL1W 10.87646866 W
SFO1 400.1324710 MHz
SI 32768
SF 400.1300094 MHz
WDW EM
SSB 0
LB 0.30 Hz
GB 0
PC 1.00
11 10 9 8 7 6 5 4 3 2 1 0 ppm
0.99 0.98 1.97 1.98 0.99 1.98 1.10
3.01 3.00

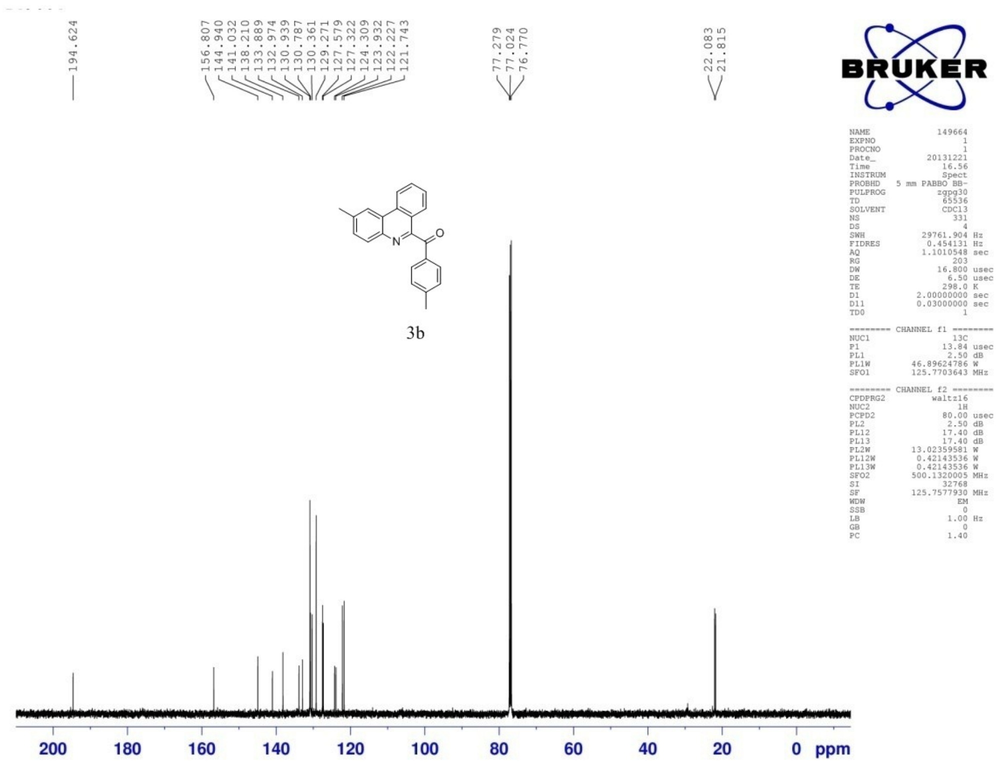
194.624
156.807 144.940 141.032 138.210 133.889 132.974 130.939 130.787 130.361 129.271 127.579 127.322 124.309 123.932 122.227 121.743
77.279 77.024 76.770
22.083 21.815
N
O
3b
BRUKER
NAME 149664
EXPNO 1
PROCNO 1
Date_ 20131221
Time 16.56
INSTRUM Spect
PROBHD 5 mm PABBO BB-
PULPROG zgpg30
TD 65536
SOLVENT CDCl3
NS 331
DS 4
SWH 29761.904 Hz
FIDRES 0.454131 Hz
AQ 1.1010548 sec
RG 203
DW 16.800 usec
DE 6.50 usec
TE 298.0 K
D1 2.00000000 sec
D11 0.03000000 sec
TD0 1
======== CHANNEL f1 ========
NUC1 13C
P1 13.84 usec
PL1 2.50 dB
PL1W 46.89624786 W
SFO1 125.7703643 MHz
======== CHANNEL f2 ========
CPDPRG2 waltz16
NUC2 1H
PCPD2 80.00 usec
PL2 2.50 dB
PL12 17.40 dB
PL13 17.40 dB
PL2W 13.02359581 W
PL12W 0.42143536 W
PL13W 0.42143536 W
SFO2 500.1320005 MHz
SI 32768
SF 125.7577930 MHz
WDW EM
SSB 0
LB 1.00 Hz
GB 0
PC 1.40
200 180 160 140 120 100 80 60 40 20 0 ppm

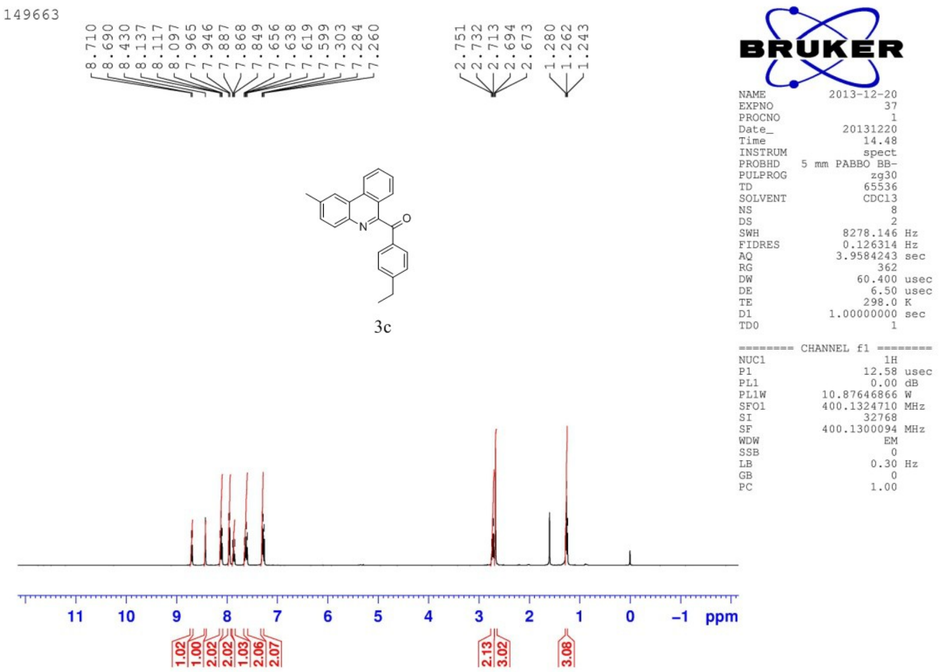
149663
8.710
8.690
8.430
8.137
8.117
8.097
7.965
7.946
7.887
7.868
7.849
7.656
7.638
7.619
7.599
7.303
7.284
7.260
2.751
2.732
2.713
2.694
2.673
1.280
1.262
1.243
N
O
3c
NAME 2013-12-20
EXPNO 37
PROCNO 1
Date_ 20131220
Time 14.48
INSTRUM spect
PROBHD 5 mm PABBO BB-
PULPROG zg30
TD 65536
SOLVENT CDCl3
NS 8
DS 2
SWH 8278.146 Hz
FIDRES 0.126314 Hz
AQ 3.9584243 sec
RG 362
DW 60.400 usec
DE 6.50 usec
TE 298.0 K
D1 1.00000000 sec
TD0 1
======== CHANNEL f1 ========
NUC1 1H
P1 12.58 usec
PL1 0.00 dB
PL1W 10.87646866 W
SFO1 400.1324710 MHz
SI 32768
SF 400.1300094 MHz
WDW EM
SSB 0
LB 0.30 Hz
GB 0
PC 1.00
11 10 9 8 7 6 5 4 3 2 1 0 −1 ppm
1.02
1.00
2.02
2.02
1.03
2.06
2.07
2.13
3.02
3.08

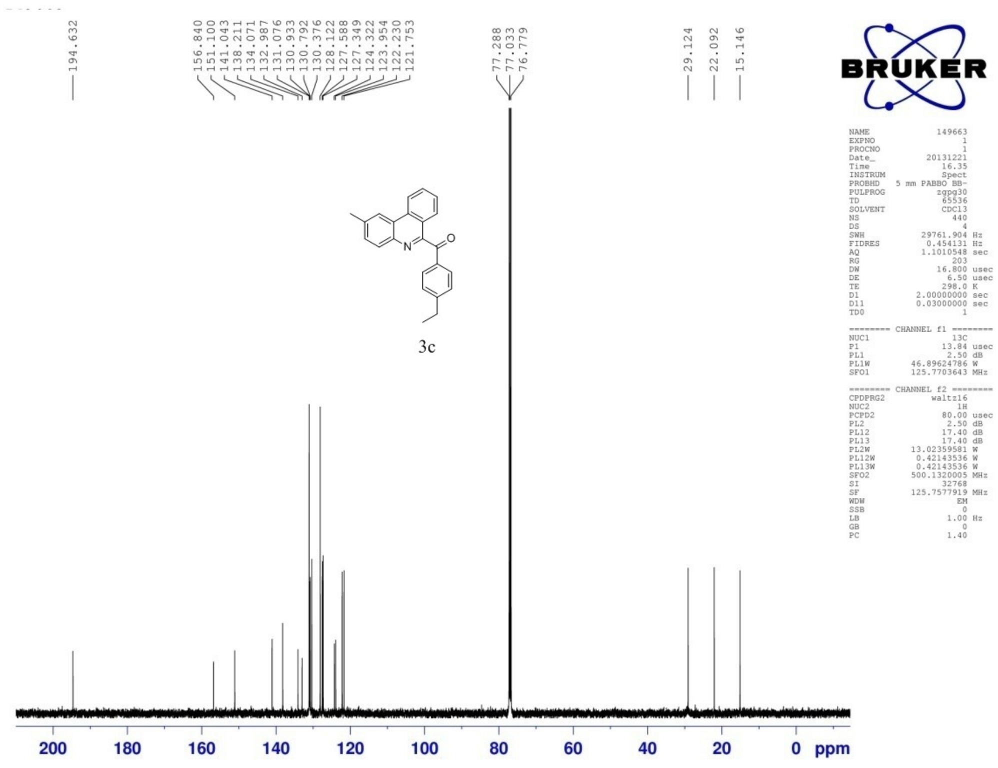
194.632
156.840
151.100
141.043
138.211
134.071
132.987
131.076
130.933
130.792
130.376
128.122
127.588
127.349
124.322
123.954
122.230
121.753
77.288
77.033
76.779
29.124
22.092
15.146
N
O
3c
NAME 149663
EXPNO 1
PROCNO 1
Date_ 20131221
Time 16.35
INSTRUM Spect
PROBHD 5 mm PABBO BB-
PULPROG zgpg30
TD 65536
SOLVENT CDCl3
NS 440
DS 4
SWH 29761.904 Hz
FIDRES 0.454131 Hz
AQ 1.1010548 sec
RG 203
DW 16.800 usec
DE 6.50 usec
TE 298.0 K
D1 2.00000000 sec
D11 0.03000000 sec
TD0 1
======== CHANNEL f1 ========
NUC1 13C
P1 13.84 usec
PL1 2.50 dB
PL1W 46.89624786 W
SFO1 125.7703643 MHz
======== CHANNEL f2 ========
CPDPRG2 waltz16
NUC2 1H
PCPD2 80.00 usec
PL2 2.50 dB
PL12 17.40 dB
PL13 17.40 dB
PL2W 13.02359581 W
PL12W 0.42143536 W
PL13W 0.42143536 W
SFO2 500.1320005 MHz
SI 32768
SF 125.7577919 MHz
WDW EM
SSB 0
LB 1.00 Hz
GB 0
PC 1.40
200 180 160 140 120 100 80 60 40 20 0 ppm

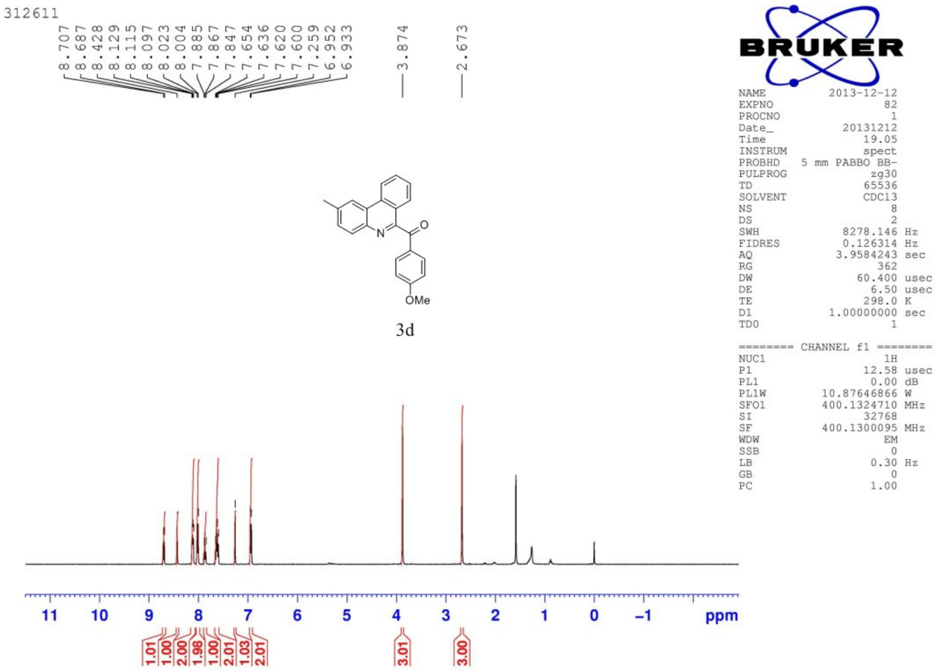
312611
8.707
8.687
8.428
8.129
8.115
8.097
8.023
8.004
7.885
7.867
7.847
7.654
7.636
7.620
7.600
7.259
6.952
6.933
3.874
2.673
OMe
3d
NAME 2013-12-12
EXPNO 82
PROCNO 1
Date_ 20131212
Time 19.05
INSTRUM spect
PROBHD 5 mm PABBO BB-
PULPROG zg30
TD 65536
SOLVENT CDCl3
NS 8
DS 2
SWH 8278.146 Hz
FIDRES 0.126314 Hz
AQ 3.9584243 sec
RG 362
DW 60.400 usec
DE 6.50 usec
TE 298.0 K
D1 1.00000000 sec
TD0 1
======== CHANNEL f1 ========
NUC1 1H
P1 12.58 usec
PL1 0.00 dB
PL1W 10.87646866 W
SFO1 400.1324710 MHz
SI 32768
SF 400.1300095 MHz
WDW EM
SSB 0
LB 0.30 Hz
GB 0
PC 1.00
11 10 9 8 7 6 5 4 3 2 1 0 –1 ppm
1.01 1.00 2.00 1.98 1.00 2.01 1.03 2.01 3.01 3.00

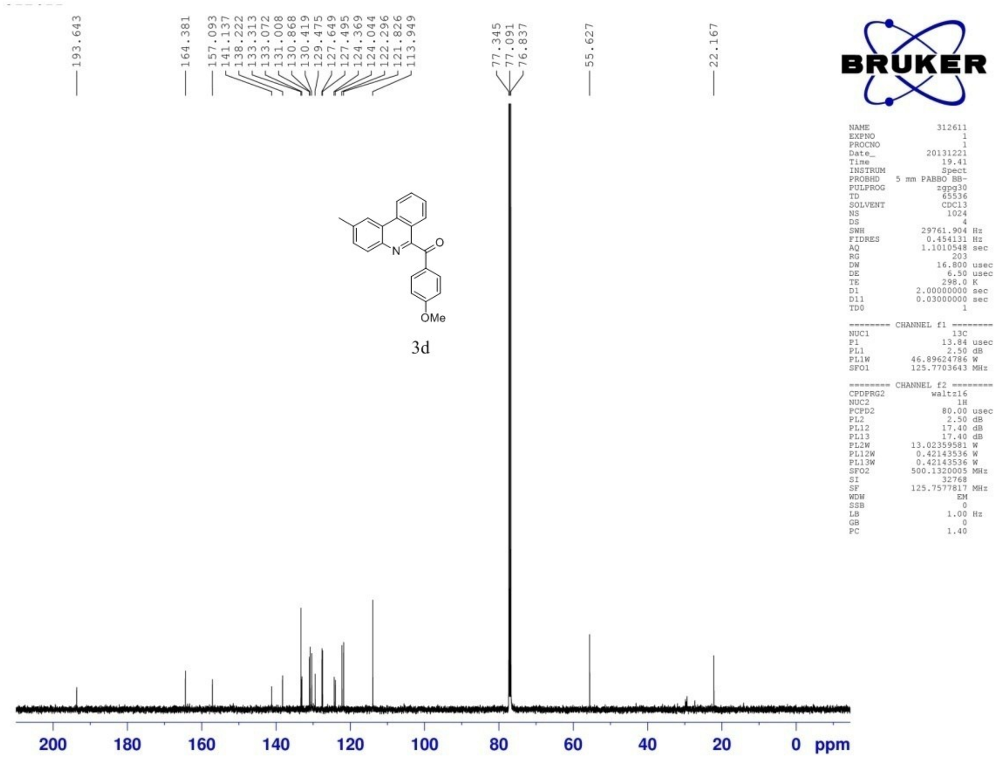
193.643
164.381
157.093
141.137
138.222
133.313
133.072
131.008
130.868
130.419
129.475
127.649
127.495
124.369
124.044
122.296
121.826
113.949
77.345
77.091
76.837
55.627
22.167
OMe
3d
NAME 312611
EXPNO 1
PROCNO 1
Date_ 20131221
Time 19.41
INSTRUM Spect
PROBHD 5 mm PABBO BB-
PULPROG zgpg30
TD 65536
SOLVENT CDCl3
NS 1024
DS 4
SWH 29761.904 Hz
FIDRES 0.454131 Hz
AQ 1.1010548 sec
RG 203
DW 16.800 usec
DE 6.50 usec
TE 298.0 K
D1 2.00000000 sec
D11 0.03000000 sec
TD0 1
======== CHANNEL f1 ========
NUC1 13C
P1 13.84 usec
PL1 2.50 dB
PL1W 46.89624786 W
SFO1 125.7703643 MHz
======== CHANNEL f2 ========
CPDPRG2 waltz16
NUC2 1H
PCPD2 80.00 usec
PL2 2.50 dB
PL12 17.40 dB
PL13 17.40 dB
PL2W 13.02359581 W
PL12W 0.42143536 W
PL13W 0.42143536 W
SFO2 500.1320005 MHz
SI 32768
SF 125.7577817 MHz
WDW EM
SSB 0
LB 1.00 Hz
GB 0
PC 1.40
200 180 160 140 120 100 80 60 40 20 0 ppm

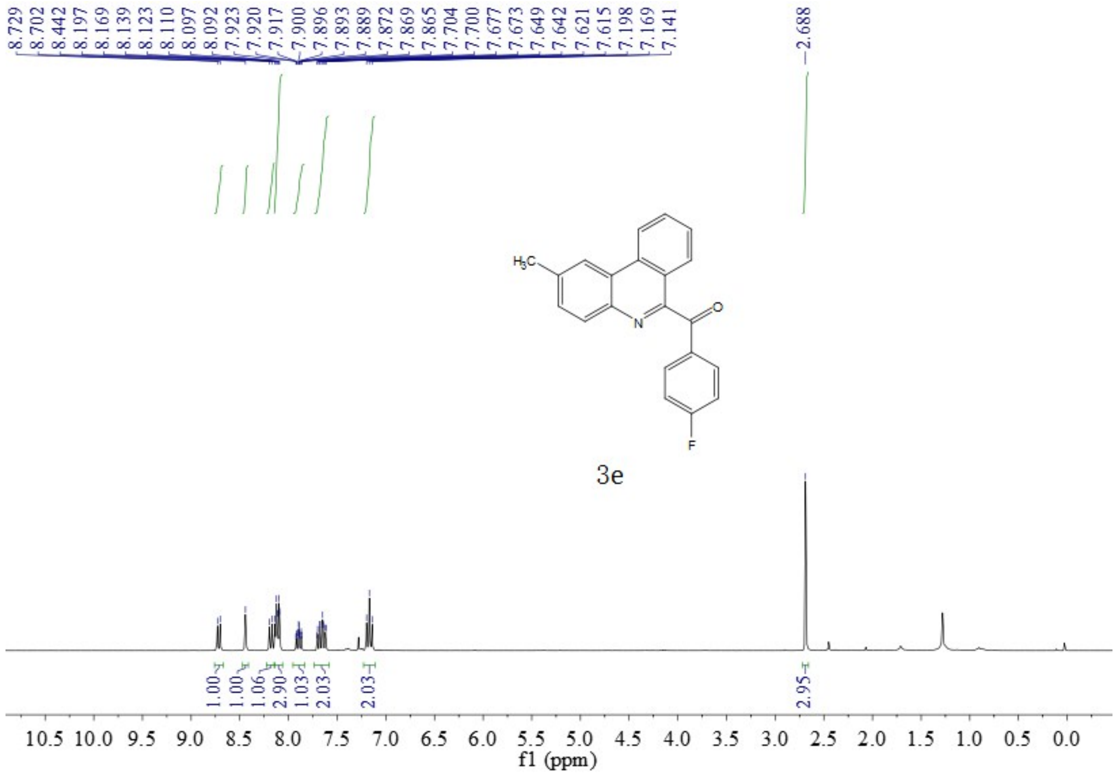
8.729
8.702
8.442
8.197
8.169
8.139
8.123
8.110
8.097
8.092
7.923
7.920
7.917
7.900
7.896
7.893
7.889
7.872
7.869
7.865
7.704
7.700
7.677
7.673
7.649
7.642
7.621
7.615
7.198
7.169
7.141
2.688
H3C
N
O
F
3e
1.00
1.00
1.06
2.90
1.03
2.03
2.03
2.95
10.5 10.0 9.5 9.0 8.5 8.0 7.5 7.0 6.5 6.0 5.5 5.0 4.5 4.0 3.5 3.0 2.5 2.0 1.5 1.0 0.5 0.0
f1 (ppm)

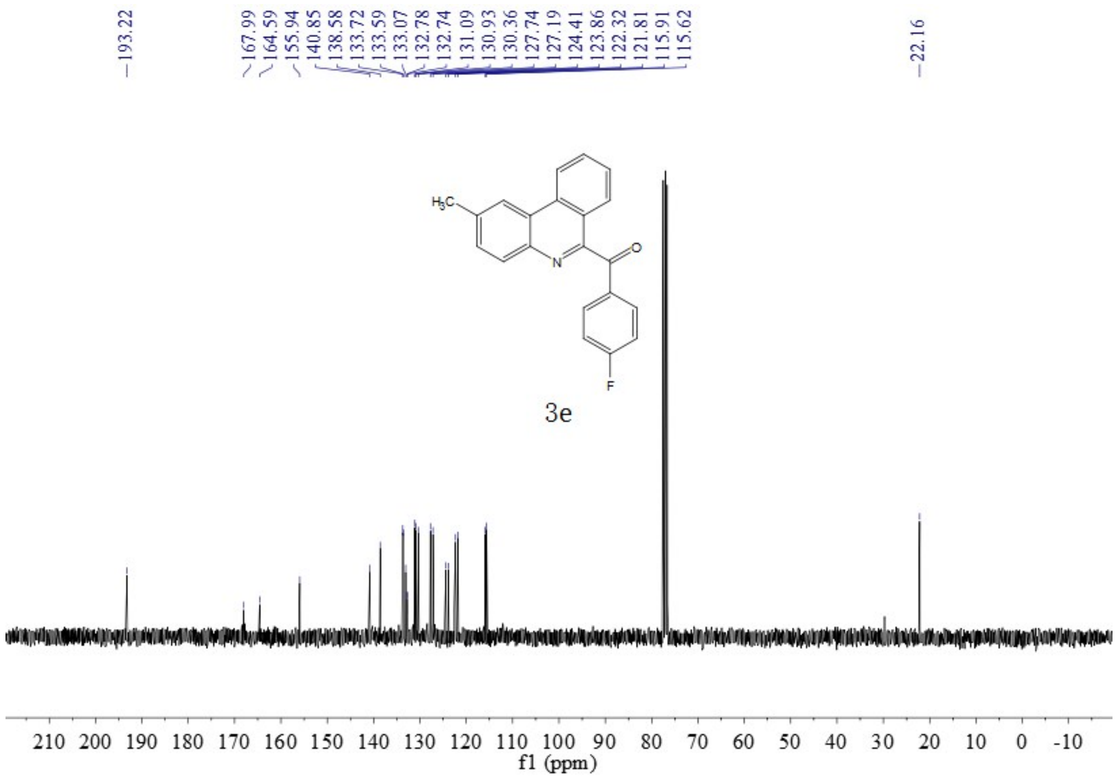
193.22
167.99
164.59
155.94
140.85
138.58
133.72
133.59
133.07
132.78
132.74
131.09
130.93
130.36
127.74
127.19
124.41
123.86
122.32
121.81
115.91
115.62
22.16
H3C
N
O
F
3e
210 200 190 180 170 160 150 140 130 120 110 100 90 80 70 60 50 40 30 20 10 0 -10
f1 (ppm)

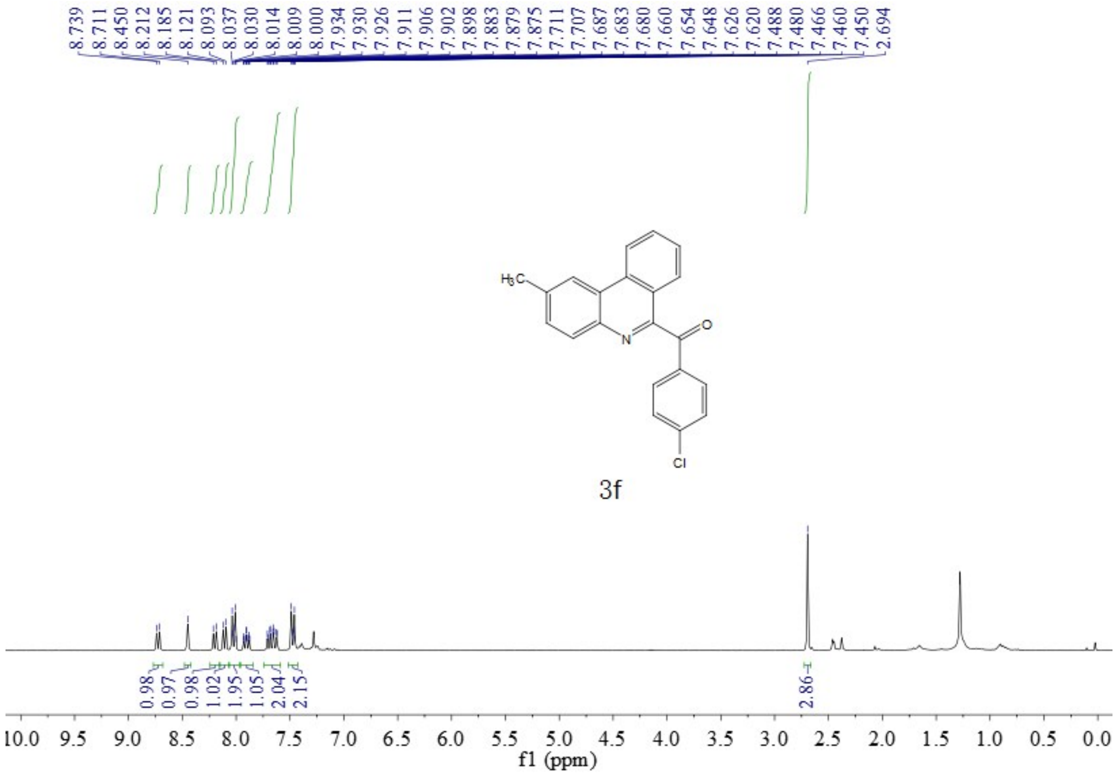
8.739
8.711
8.450
8.212
8.185
8.121
8.093
8.037
8.030
8.014
8.009
8.000
7.934
7.930
7.926
7.911
7.906
7.902
7.898
7.883
7.879
7.875
7.711
7.707
7.687
7.683
7.680
7.660
7.654
7.648
7.626
7.620
7.488
7.480
7.466
7.460
7.450
2.694
H_3C
N
O
Cl
3f
0.98
0.97
0.98
1.02
1.95
1.05
2.04
2.15
2.86
10.0 9.5 9.0 8.5 8.0 7.5 7.0 6.5 6.0 5.5 5.0 4.5 4.0 3.5 3.0 2.5 2.0 1.5 1.0 0.5 0.0
f1 (ppm)

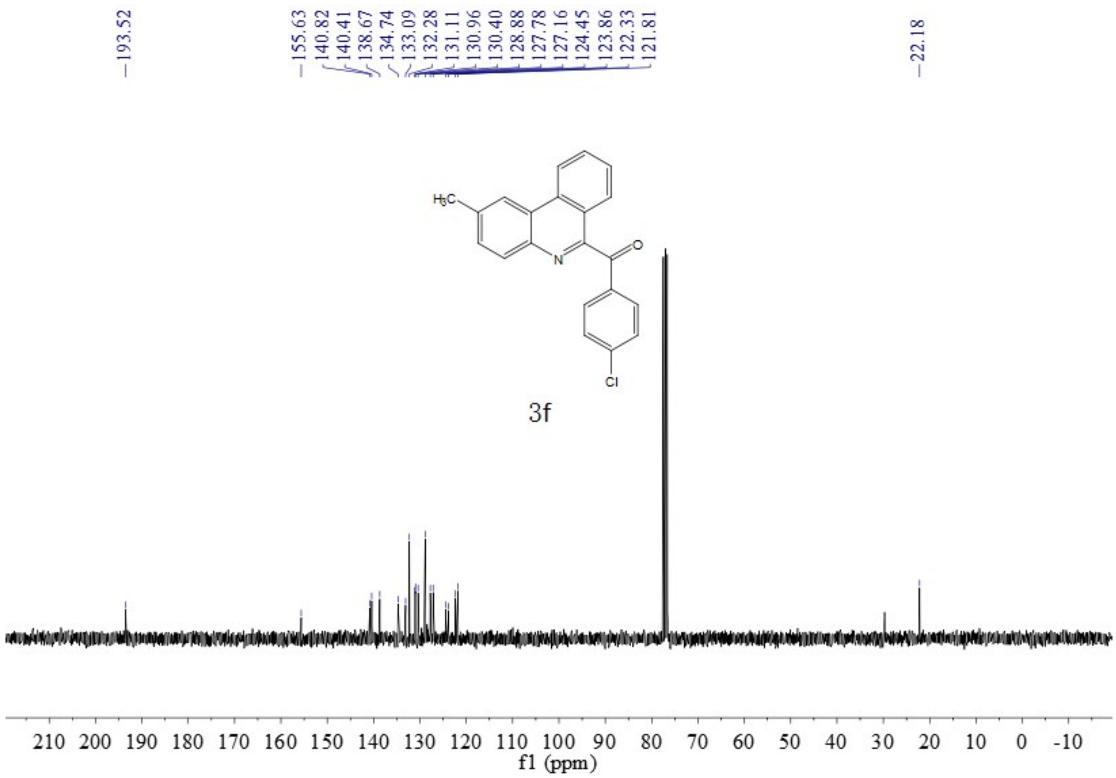
193.52
155.63
140.82
140.41
138.67
134.74
133.09
132.28
131.11
130.96
130.40
128.88
127.78
127.16
124.45
123.86
122.33
121.81
22.18
H_3C
N
O
Cl
3f
210 200 190 180 170 160 150 140 130 120 110 100 90 80 70 60 50 40 30 20 10 0 -10
f1 (ppm)

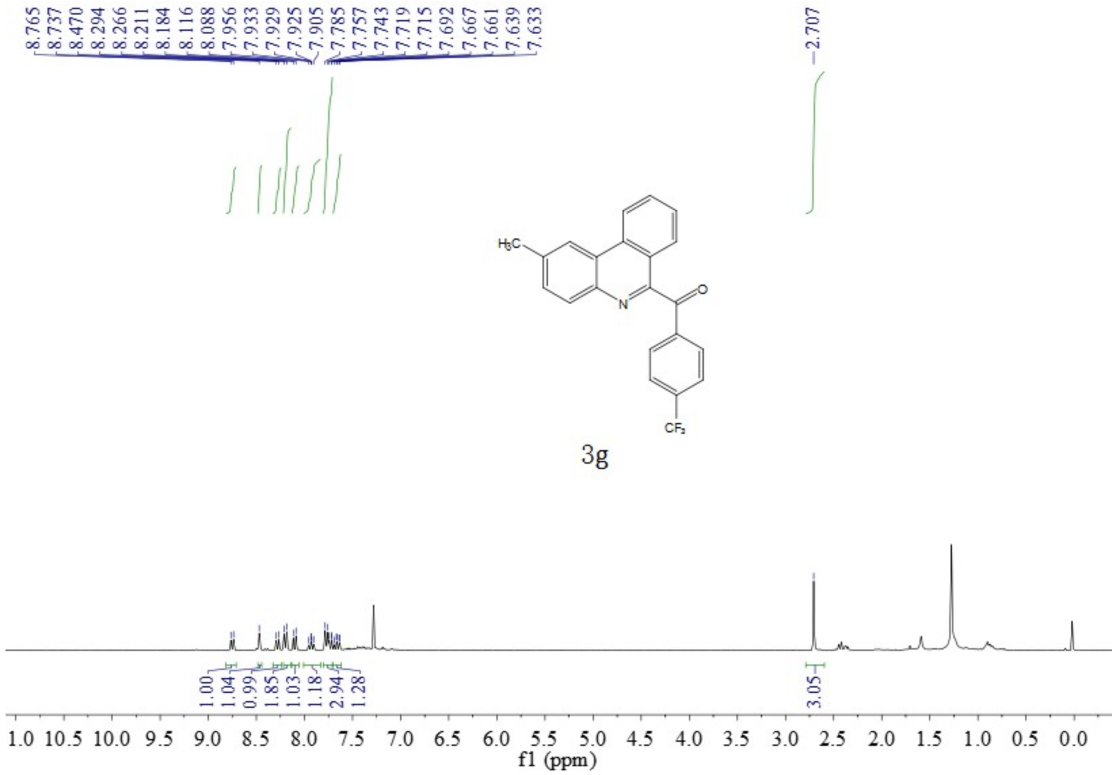
8.765
8.737
8.470
8.294
8.266
8.211
8.184
8.116
8.088
7.956
7.933
7.929
7.925
7.905
7.785
7.757
7.743
7.719
7.715
7.692
7.667
7.661
7.639
7.633
2.707
H3C
N
O
CF3
3g
1.00
1.04
0.99
1.85
1.03
1.18
2.94
1.28
3.05
1.0 10.5 10.0 9.5 9.0 8.5 8.0 7.5 7.0 6.5 6.0 5.5 5.0 4.5 4.0 3.5 3.0 2.5 2.0 1.5 1.0 0.5 0.0
f1 (ppm)

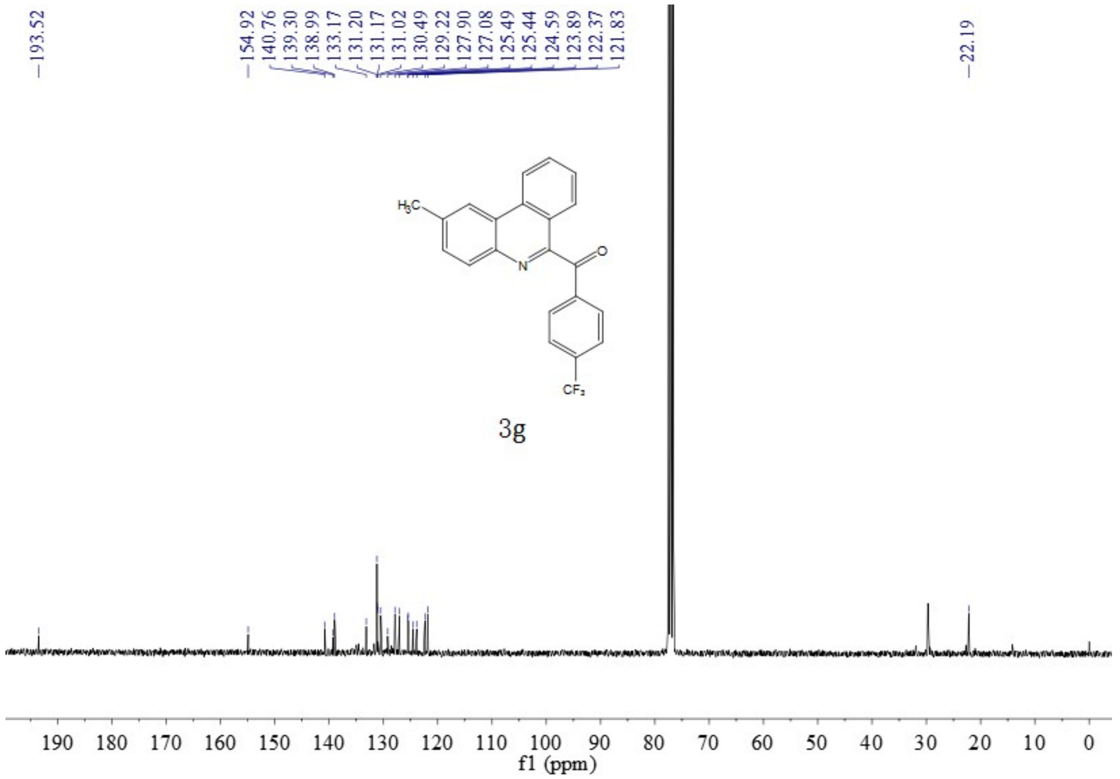
193.52
154.92
140.76
139.30
138.99
133.17
131.20
131.17
131.02
130.49
129.22
127.90
127.08
125.49
125.44
124.59
123.89
122.37
121.83
22.19
H3C
N
O
CF3
3g
190 180 170 160 150 140 130 120 110 100 90 80 70 60 50 40 30 20 10 0
f1 (ppm)

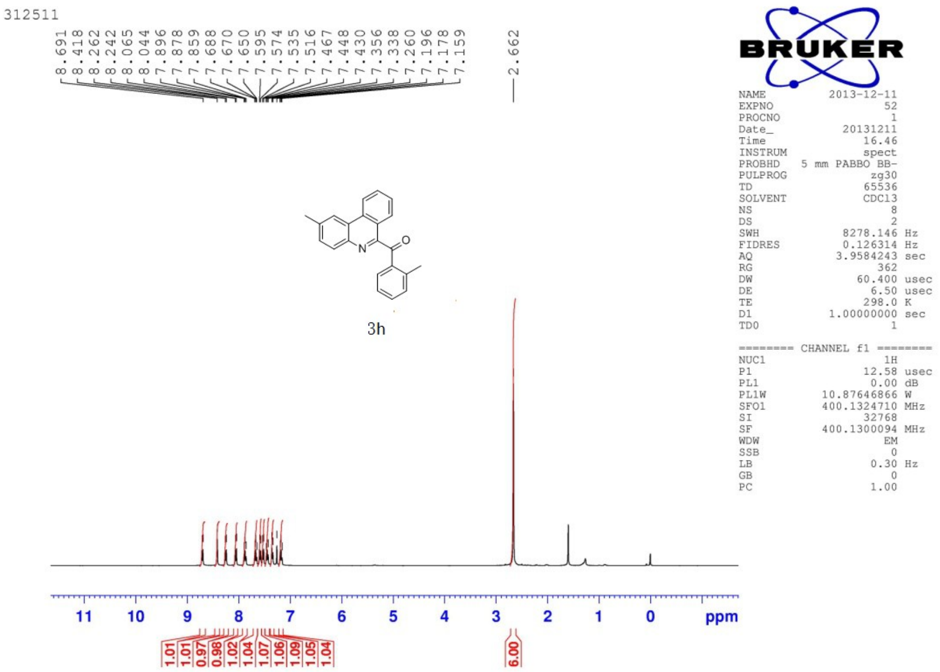
312511
8.691 8.418 8.262 8.242 8.065 8.044 7.896 7.878 7.859 7.688 7.670 7.650 7.595 7.574 7.535 7.516 7.467 7.448 7.430 7.356 7.338 7.260 7.196 7.178 7.159
2.662
BRUKER
NAME 2013-12-11
EXPNO 52
PROCNO 1
Date_ 20131211
Time 16.46
INSTRUM spect
PROBHD 5 mm PABBO BB-
PULPROG zg30
TD 65536
SOLVENT CDCl3
NS 8
DS 2
SWH 8278.146 Hz
FIDRES 0.126314 Hz
AQ 3.9584243 sec
RG 362
DW 60.400 usec
DE 6.50 usec
TE 298.0 K
D1 1.00000000 sec
TD0 1
======== CHANNEL f1 ========
NUC1 1H
P1 12.58 usec
PL1 0.00 dB
PL1W 10.87646866 W
SFO1 400.1324710 MHz
SI 32768
SF 400.1300094 MHz
WDW EM
SSB 0
LB 0.30 Hz
GB 0
PC 1.00
N
O
3h
11 10 9 8 7 6 5 4 3 2 1 0 ppm
1.01 1.01 0.97 0.98 1.02 1.04 1.07 1.06 1.09 1.05 1.04
6.00

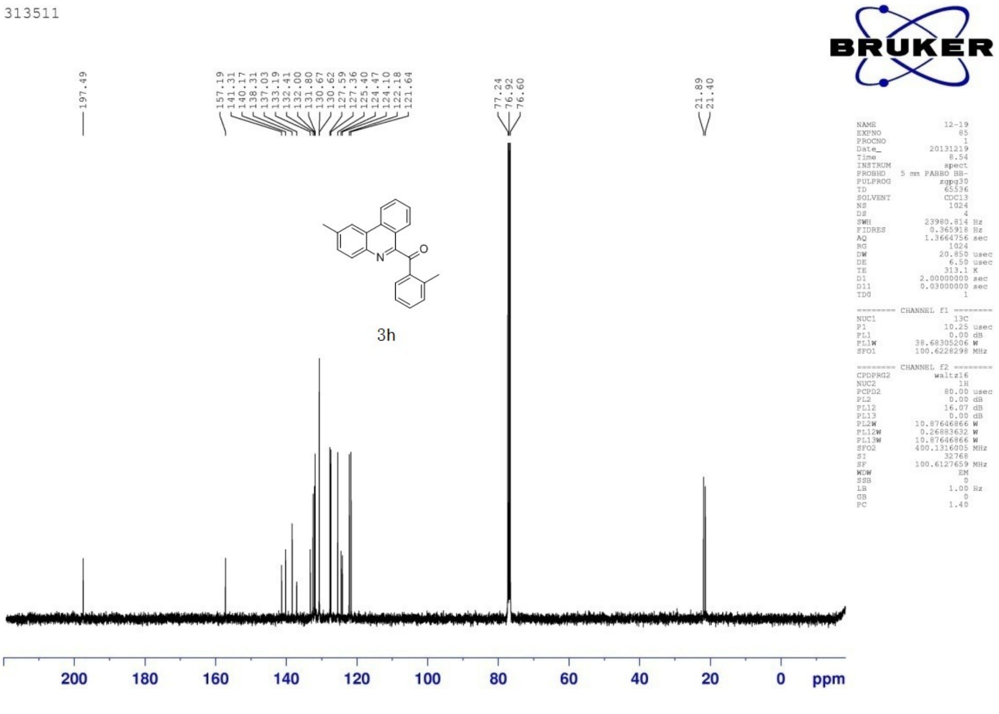
313511
197.49
157.19 141.31 140.17 138.31 137.03 133.19 132.41 132.00 131.80 130.67 130.62 127.59 127.36 125.40 124.47 124.10 122.18 121.64
77.24 76.92 76.60
21.89 21.40
BRUKER
N
O
3h
200 180 160 140 120 100 80 60 40 20 0 ppm

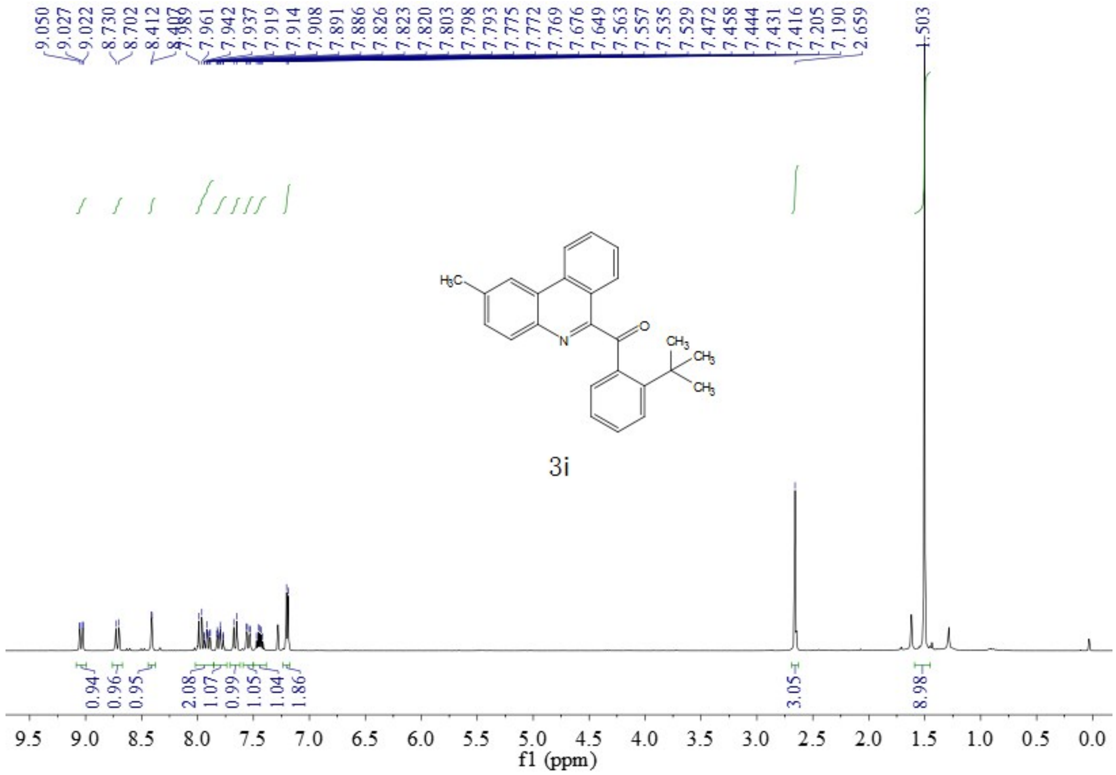
9.050
9.027
9.022
8.730
8.702
8.412
7.961
7.942
7.937
7.919
7.914
7.908
7.891
7.886
7.826
7.823
7.820
7.803
7.798
7.793
7.775
7.772
7.769
7.676
7.649
7.563
7.557
7.535
7.529
7.472
7.458
7.444
7.431
7.416
7.205
7.190
2.659
1.503
H3C
N
O
CH3
CH3
CH3
3i
0.94
0.96
0.95
2.08
1.07
0.99
1.05
1.04
1.86
3.05
8.98
9.5 9.0 8.5 8.0 7.5 7.0 6.5 6.0 5.5 5.0 4.5 4.0 3.5 3.0 2.5 2.0 1.5 1.0 0.5 0.0
f1 (ppm)

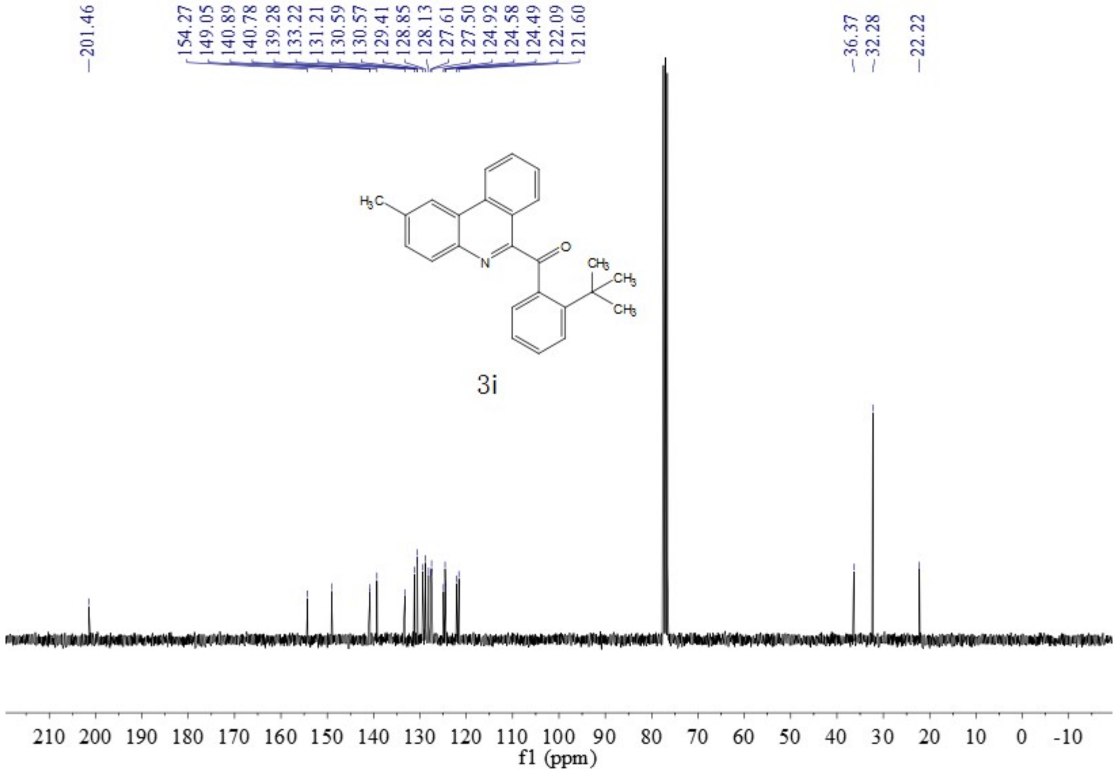
201.46
154.27
149.05
140.89
140.78
139.28
133.22
131.21
130.59
130.57
129.41
128.85
128.13
127.61
127.50
124.92
124.58
124.49
122.09
121.60
36.37
32.28
22.22
H3C
N
O
CH3
CH3
CH3
3i
210 200 190 180 170 160 150 140 130 120 110 100 90 80 70 60 50 40 30 20 10 0 -10
f1 (ppm)

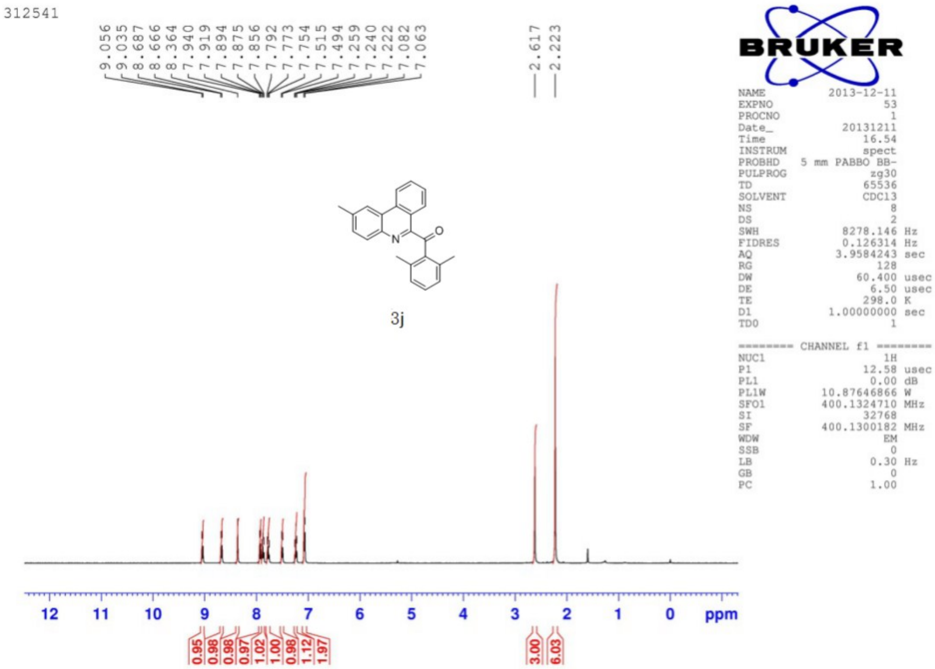
312541
9.056
9.035
8.687
8.666
8.364
7.940
7.919
7.894
7.875
7.856
7.792
7.773
7.754
7.515
7.494
7.259
7.240
7.222
7.082
7.063
2.617
2.223
N
O
3j
BRUKER
NAME 2013-12-11
EXPNO 53
PROCNO 1
Date_ 20131211
Time 16.54
INSTRUM spect
PROBHD 5 mm PABBO BB-
PULPROG zg30
TD 65536
SOLVENT CDCl3
NS 8
DS 2
SWH 8278.146 Hz
FIDRES 0.126314 Hz
AQ 3.9584243 sec
RG 128
DW 60.400 usec
DE 6.50 usec
TE 298.0 K
D1 1.00000000 sec
TD0 1
======== CHANNEL f1 ========
NUC1 1H
P1 12.58 usec
PL1 0.00 dB
PL1W 10.87646866 W
SFO1 400.1324710 MHz
SI 32768
SF 400.1300182 MHz
WDW EM
SSB 0
LB 0.30 Hz
GB 0
PC 1.00
12 11 10 9 8 7 6 5 4 3 2 1 0 ppm
0.95
0.98
0.98
0.97
1.02
1.00
0.98
1.12
1.97
3.00
6.03

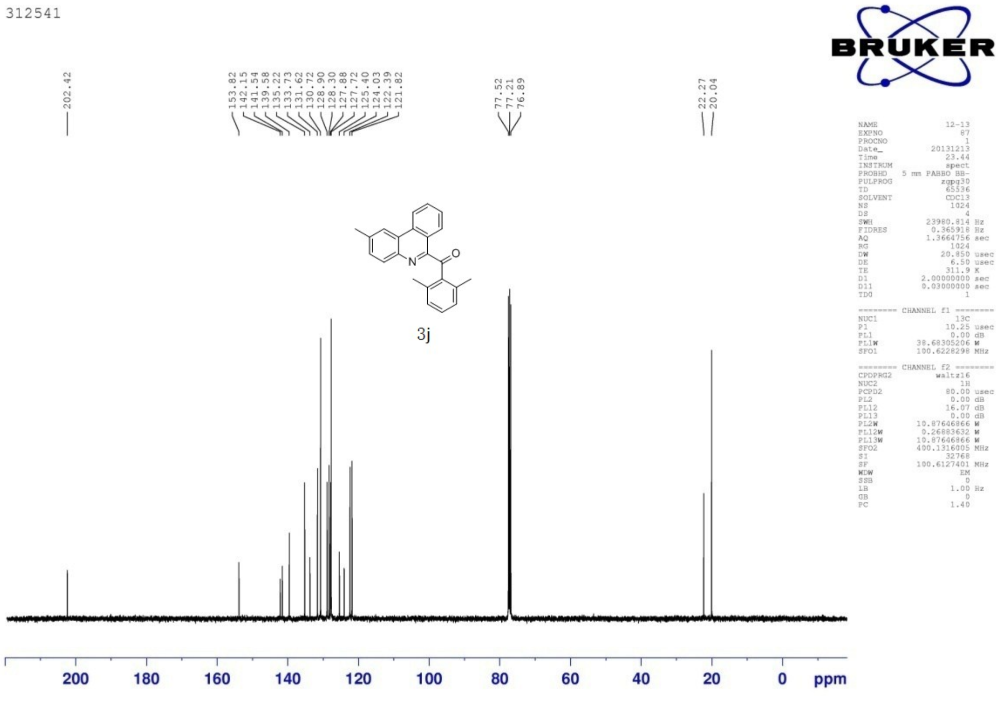
312541
202.42
153.82
142.15
141.54
139.58
135.22
133.73
131.62
130.72
128.90
128.30
127.88
127.72
125.40
124.03
122.39
121.82
77.52
77.21
76.89
22.27
20.04
N
O
3j
BRUKER
NAME 12-13
EXPNO 87
PROCNO 1
Date_ 20131213
Time 23.44
INSTRUM spect
PROBHD 5 mm PABBO BB-
PULPROG zgpg30
TD 65536
SOLVENT CDCl3
NS 1024
DS 4
SWH 23980.814 Hz
FIDRES 0.365918 Hz
AQ 1.3664756 sec
RG 1024
DW 20.850 usec
DE 6.50 usec
TE 311.9 K
D1 2.00000000 sec
D11 0.03000000 sec
TD0 1
======== CHANNEL f1 ========
NUC1 13C
P1 10.25 usec
PL1 0.00 dB
PL1W 38.68305206 W
SFO1 100.6228298 MHz
======== CHANNEL f2 ========
CPDPRG2 waltz16
NUC2 1H
PCPD2 80.00 usec
PL2 0.00 dB
PL12 16.07 dB
PL13 0.00 dB
PL2W 10.87646866 W
PL12W 0.26883632 W
PL13W 10.87646866 W
SFO2 400.1316005 MHz
SI 32768
SF 100.6127401 MHz
WDW EM
SSB 0
LB 1.00 Hz
GB 0
PC 1.40
200 180 160 140 120 100 80 60 40 20 0 ppm

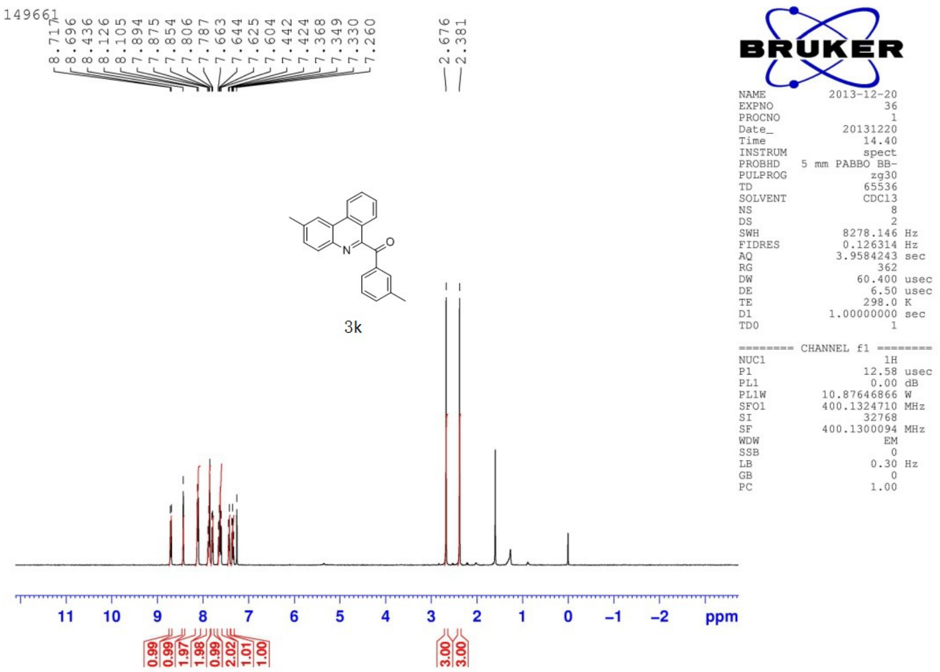
149661
8.717
8.696
8.436
8.126
8.105
7.894
7.875
7.854
7.806
7.787
7.663
7.644
7.625
7.604
7.442
7.424
7.368
7.349
7.330
7.260
2.676
2.381
3k
BRUKER
NAME 2013-12-20
EXPNO 36
PROCNO 1
Date_ 20131220
Time 14.40
INSTRUM spect
PROBHD 5 mm PABBO BB-
PULPROG zg30
TD 65536
SOLVENT CDCl3
NS 8
DS 2
SWH 8278.146 Hz
FIDRES 0.126314 Hz
AQ 3.9584243 sec
RG 362
DW 60.400 usec
DE 6.50 usec
TE 298.0 K
D1 1.00000000 sec
TD0 1
======== CHANNEL f1 ========
NUC1 1H
P1 12.58 usec
PL1 0.00 dB
PL1W 10.87646866 W
SFO1 400.1324710 MHz
SI 32768
SF 400.1300094 MHz
WDW EM
SSB 0
LB 0.30 Hz
GB 0
PC 1.00
0.99
0.99
1.97
1.98
0.99
2.02
1.01
1.00
3.00
3.00
ppm

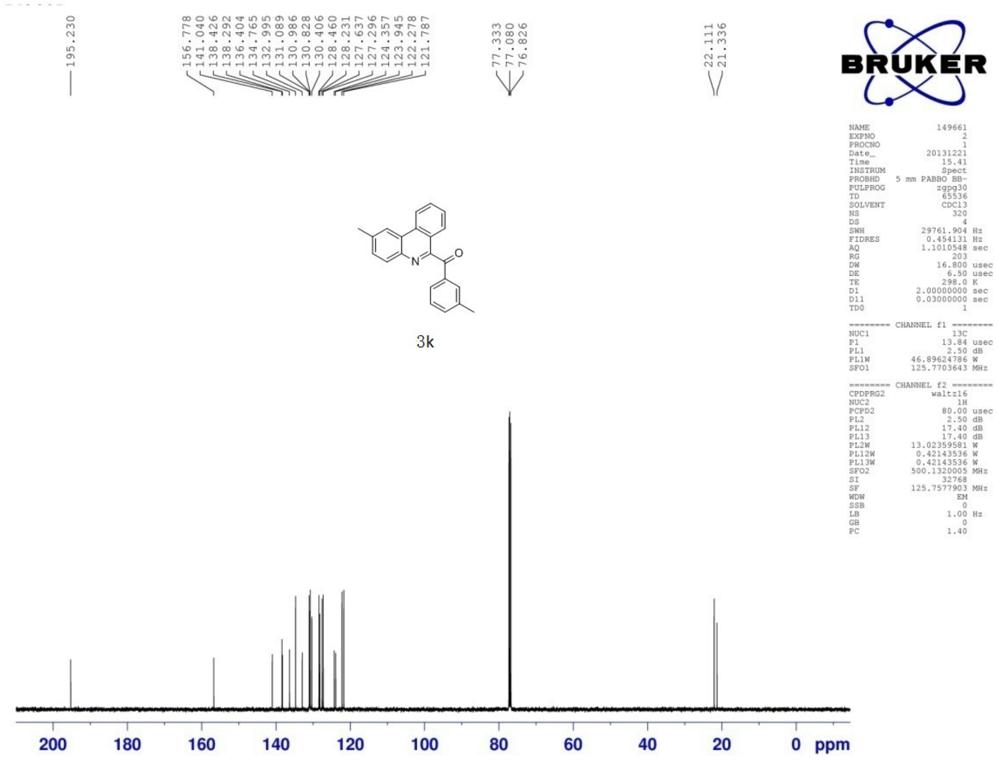
195.230
156.778
141.040
138.426
138.292
136.404
134.765
132.995
131.089
130.986
130.828
130.406
128.460
128.231
127.637
127.296
124.357
123.945
122.278
121.787
77.333
77.080
76.826
22.111
21.336
3k
BRUKER
ppm

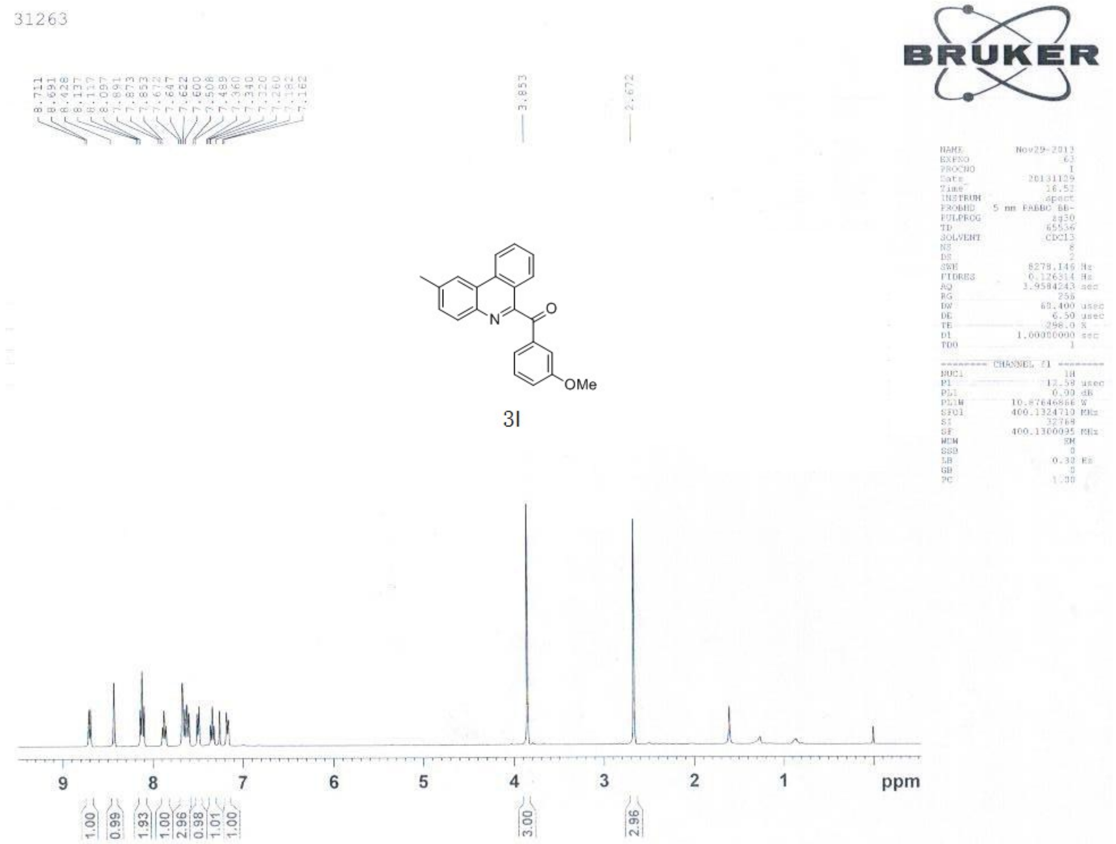
31263
OMe
3l
ppm

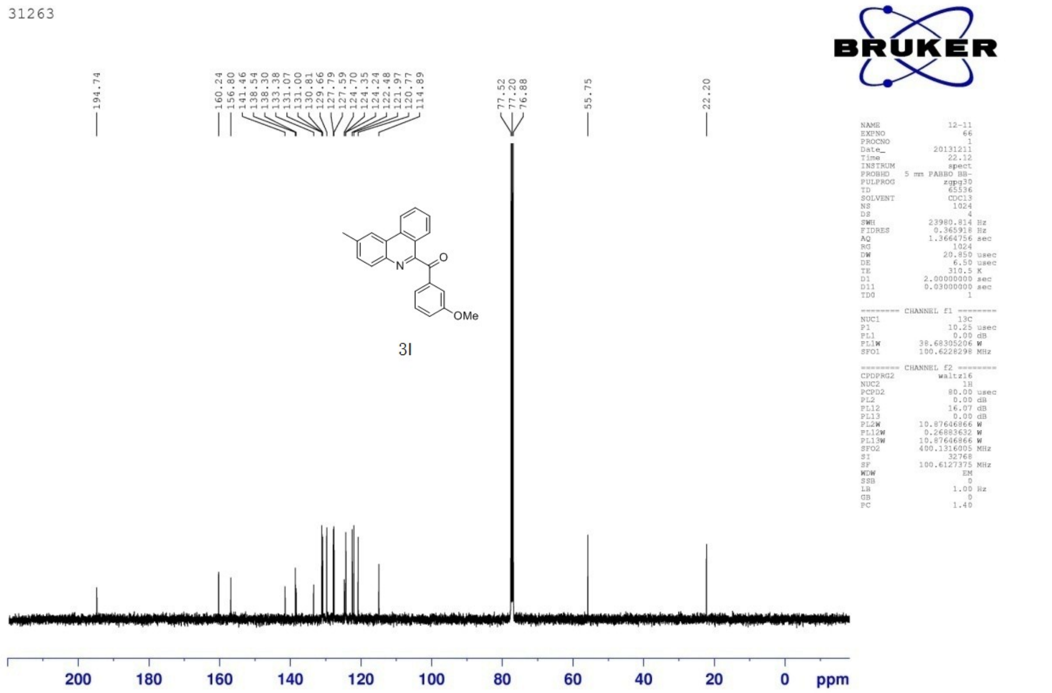
31263
OMe
3l
ppm

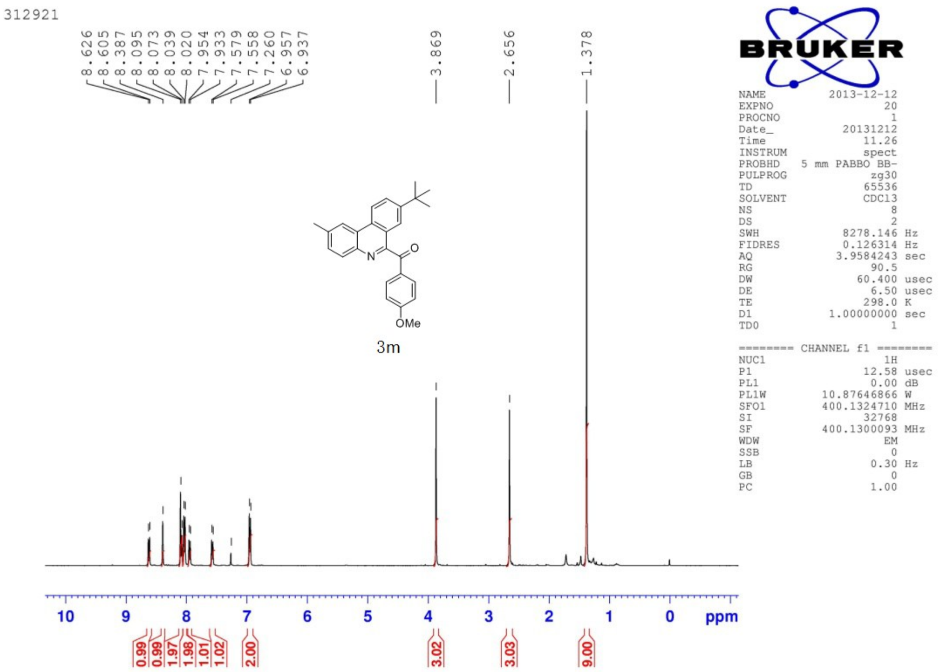
312921
8.626
8.605
8.387
8.095
8.073
8.039
8.020
7.954
7.933
7.579
7.558
7.260
6.957
6.937
3.869
2.656
1.378
OMe
N
O
3m
BRUKER
NAME 2013-12-12
EXPNO 20
PROCNO 1
Date_ 20131212
Time 11.26
INSTRUM spect
PROBHD 5 mm PABBO BB-
PULPROG zg30
TD 65536
SOLVENT CDCl3
NS 8
DS 2
SWH 8278.146 Hz
FIDRES 0.126314 Hz
AQ 3.9584243 sec
RG 90.5
DW 60.400 usec
DE 6.50 usec
TE 298.0 K
D1 1.00000000 sec
TD0 1
======== CHANNEL f1 ========
NUC1 1H
P1 12.58 usec
PL1 0.00 dB
PL1W 10.87646866 W
SFO1 400.1324710 MHz
SI 32768
SF 400.1300093 MHz
WDW EM
SSB 0
LB 0.30 Hz
GB 0
PC 1.00
10 9 8 7 6 5 4 3 2 1 0 ppm
0.99
0.99
1.97
1.98
1.01
1.02
2.00
3.02
3.03
9.00

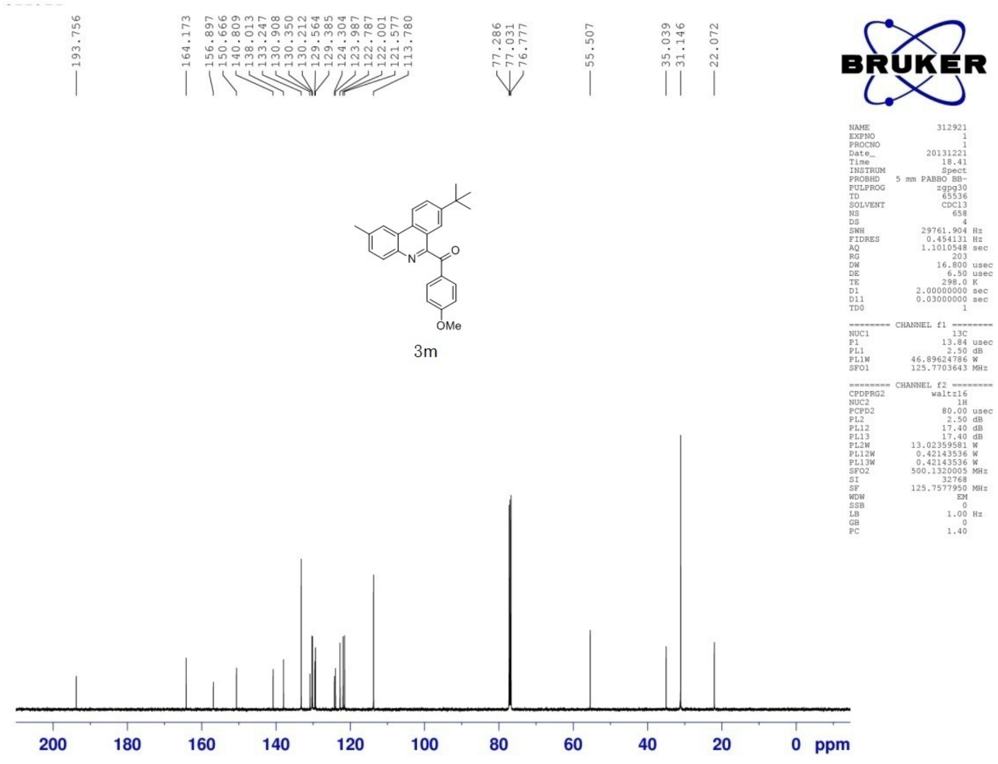
193.756
164.173
156.897
150.666
140.809
138.013
133.247
130.908
130.350
130.212
129.564
129.385
124.304
123.987
122.787
122.001
121.577
113.780
77.286
77.031
76.777
55.507
35.039
31.146
22.072
BRUKER
N
O
OMe
3m
200 180 160 140 120 100 80 60 40 20 0 ppm

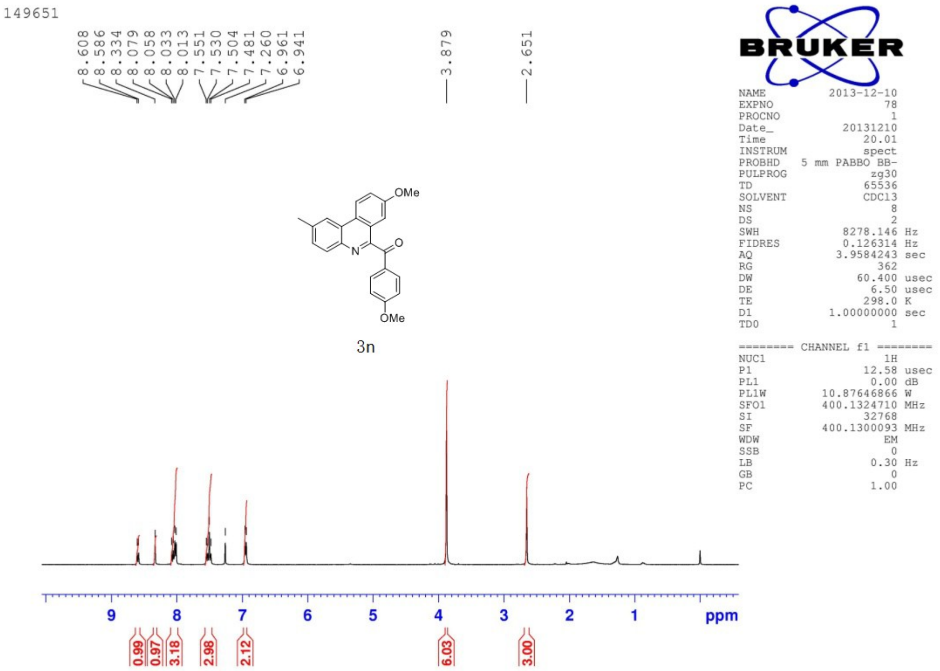
149651
8.608 8.586 8.334 8.079 8.058 8.033 8.013 7.551 7.530 7.504 7.481 7.260 6.961 6.941
3.879
2.651
OMe
N
O
OMe
3n
BRUKER
NAME 2013-12-10
EXPNO 78
PROCNO 1
Date_ 20131210
Time 20.01
INSTRUM spect
PROBHD 5 mm PABBO BB-
PULPROG zg30
TD 65536
SOLVENT CDCl3
NS 8
DS 2
SWH 8278.146 Hz
FIDRES 0.126314 Hz
AQ 3.9584243 sec
RG 362
DW 60.400 usec
DE 6.50 usec
TE 298.0 K
D1 1.00000000 sec
TD0 1
======== CHANNEL f1 ========
NUC1 1H
P1 12.58 usec
PL1 0.00 dB
PL1W 10.87646866 W
SFO1 400.1324710 MHz
SI 32768
SF 400.1300093 MHz
WDW EM
SSB 0
LB 0.30 Hz
GB 0
PC 1.00
9 8 7 6 5 4 3 2 1 ppm
0.99 0.97 3.18 2.98 2.12 6.03 3.00

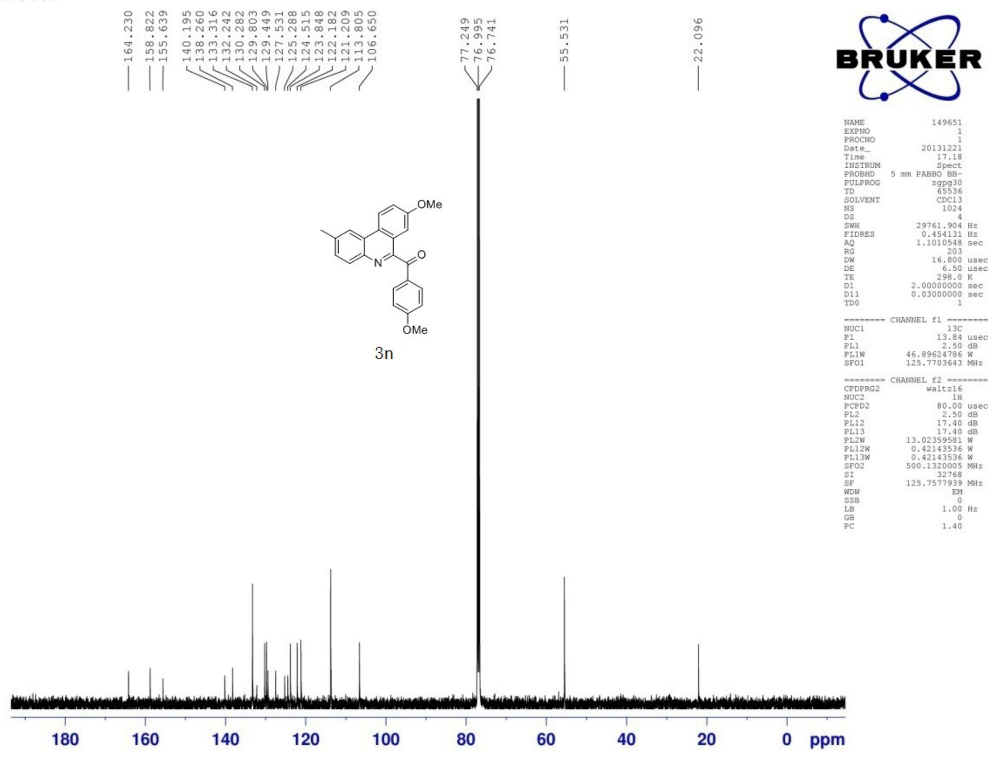
164.230 158.822 155.639 140.195 138.260 133.316 132.242 130.282 129.803 129.449 127.531 125.288 124.515 123.848 122.182 121.209 113.805 106.650
77.249 76.995 76.741
55.531
22.096
OMe
N
O
OMe
3n
BRUKER
NAME 149651
EXPNO 1
PROCNO 1
Date_ 20131221
Time 17.18
INSTRUM spect
PROBHD 5 mm PABBO BB-
PULPROG zgpg30
TD 65536
SOLVENT CDCl3
NS 1024
DS 4
SWH 29761.904 Hz
FIDRES 0.454131 Hz
AQ 1.1010548 sec
RG 203
DW 16.800 usec
DE 6.50 usec
TE 298.0 K
D1 2.00000000 sec
D11 0.03000000 sec
TD0 1
======== CHANNEL f1 ========
NUC1 13C
P1 13.84 usec
PL1 2.50 dB
PL1W 46.89624786 W
SFO1 125.7703643 MHz
======== CHANNEL f2 ========
CPDPRG2 waltz16
NUC2 1H
PCPD2 80.00 usec
PL2 2.50 dB
PL12 17.40 dB
PL13 17.40 dB
PL2W 13.02359581 W
PL12W 0.42143536 W
PL13W 0.42143536 W
SFO2 500.1320005 MHz
SI 32768
SF 125.7577939 MHz
WDW EM
SSB 0
LB 1.00 Hz
GB 0
PC 1.40
180 160 140 120 100 80 60 40 20 0 ppm

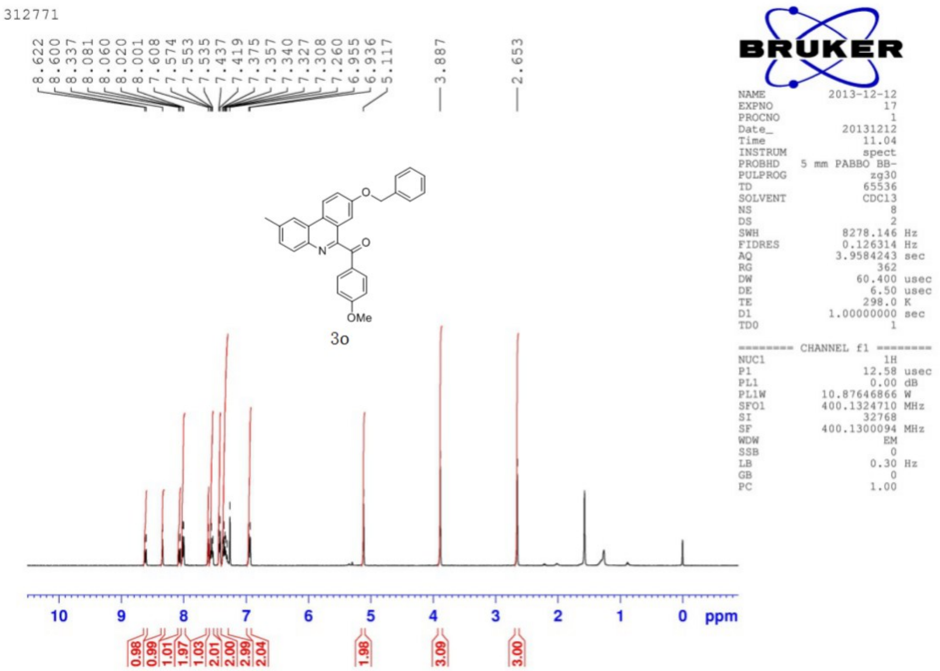

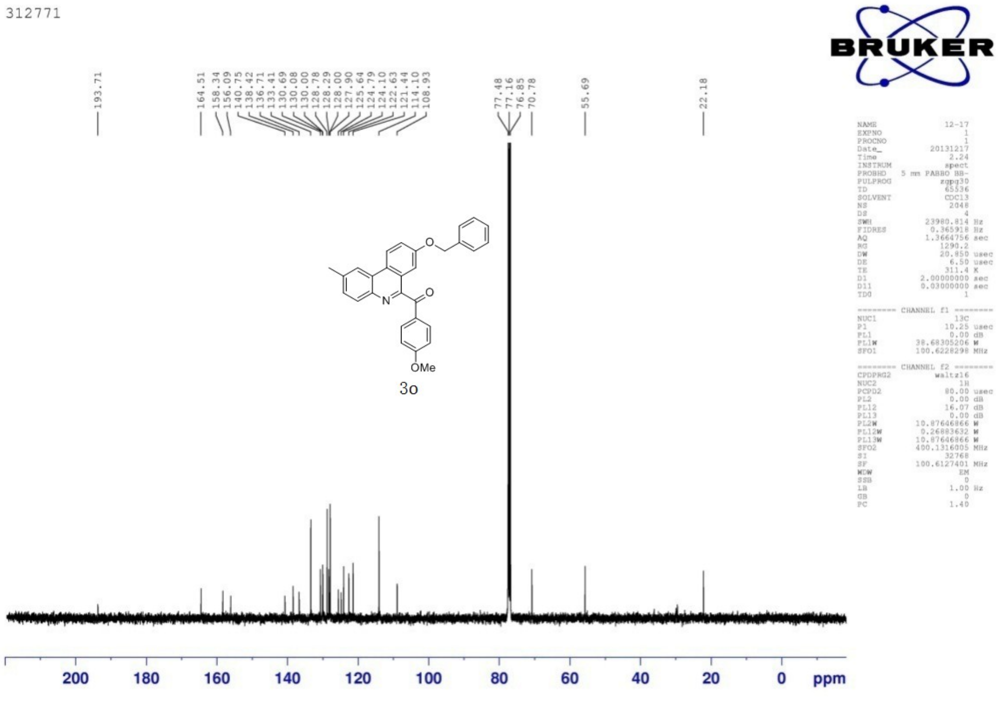

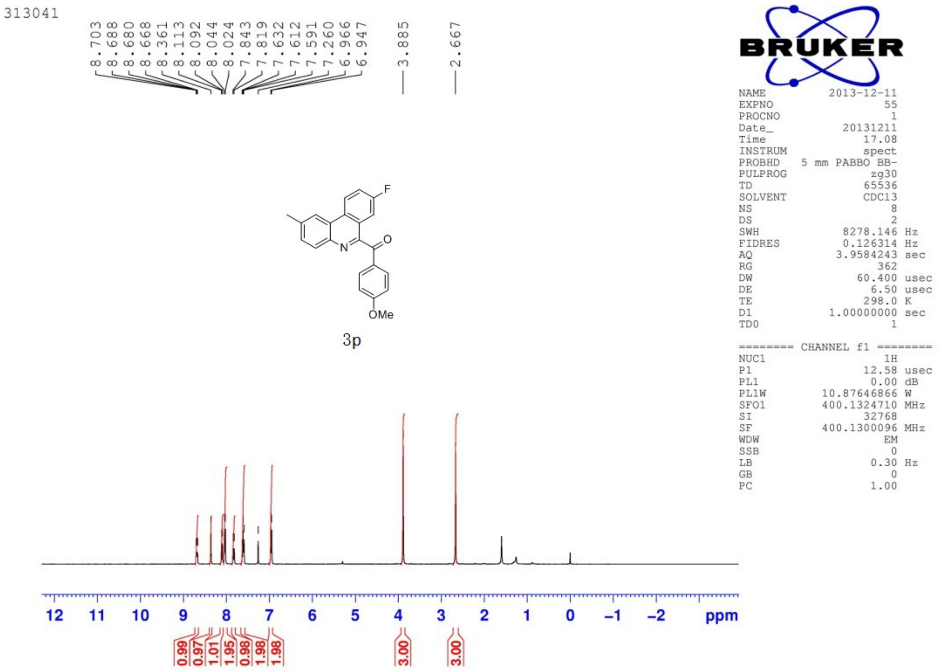
313041
8.703
8.688
8.680
8.668
8.361
8.113
8.092
8.044
8.024
7.843
7.819
7.632
7.612
7.591
7.260
6.966
6.947
3.885
2.667
F
O
N
OMe
3p
BRUKER
NAME 2013-12-11
EXPNO 55
PROCNO 1
Date_ 20131211
Time 17.08
INSTRUM spect
PROBHD 5 mm PABBO BB-
PULPROG zg30
TD 65536
SOLVENT CDCl3
NS 8
DS 2
SWH 8278.146 Hz
FIDRES 0.126314 Hz
AQ 3.9584243 sec
RG 362
DW 60.400 usec
DE 6.50 usec
TE 298.0 K
D1 1.00000000 sec
TD0 1
======== CHANNEL f1 ========
NUC1 1H
P1 12.58 usec
PL1 0.00 dB
PL1W 10.87646866 W
SFO1 400.1324710 MHz
SI 32768
SF 400.1300096 MHz
WDW EM
SSB 0
LB 0.30 Hz
GB 0
PC 1.00
12 11 10 9 8 7 6 5 4 3 2 1 0 -1 -2 ppm
0.99
0.97
1.01
1.95
0.98
1.98
1.98
3.00
3.00

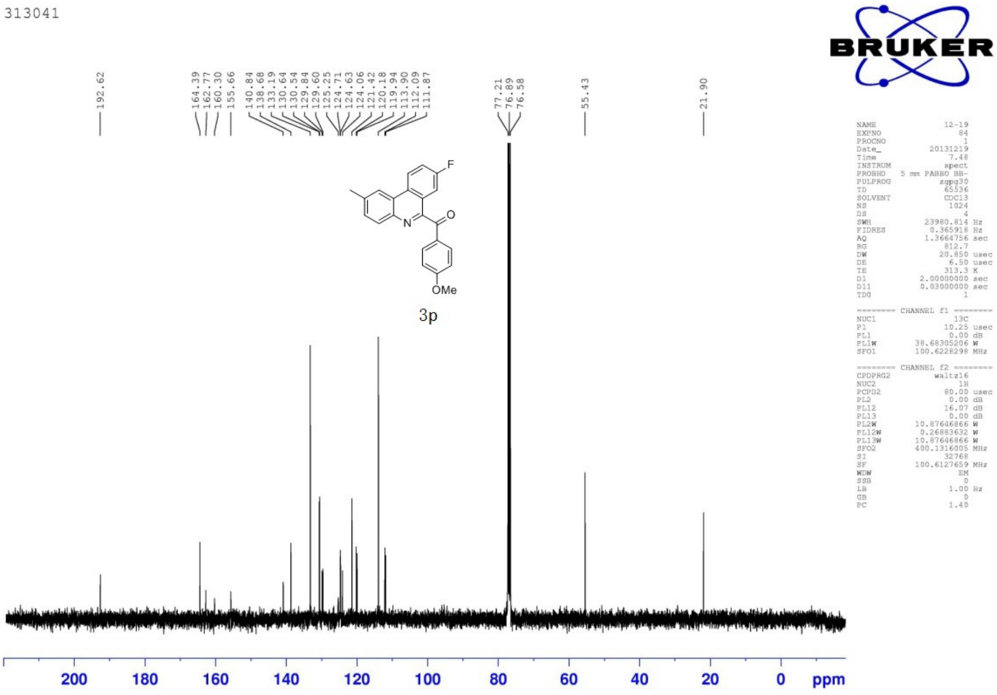
313041
192.62
164.39
162.77
160.30
155.66
140.84
138.68
133.19
130.64
130.54
129.84
129.60
125.25
124.71
124.63
124.06
121.42
120.18
119.94
113.90
112.09
111.87
77.21
76.89
76.58
55.43
21.90
F
O
N
OMe
3p
BRUKER
200 180 160 140 120 100 80 60 40 20 0 ppm

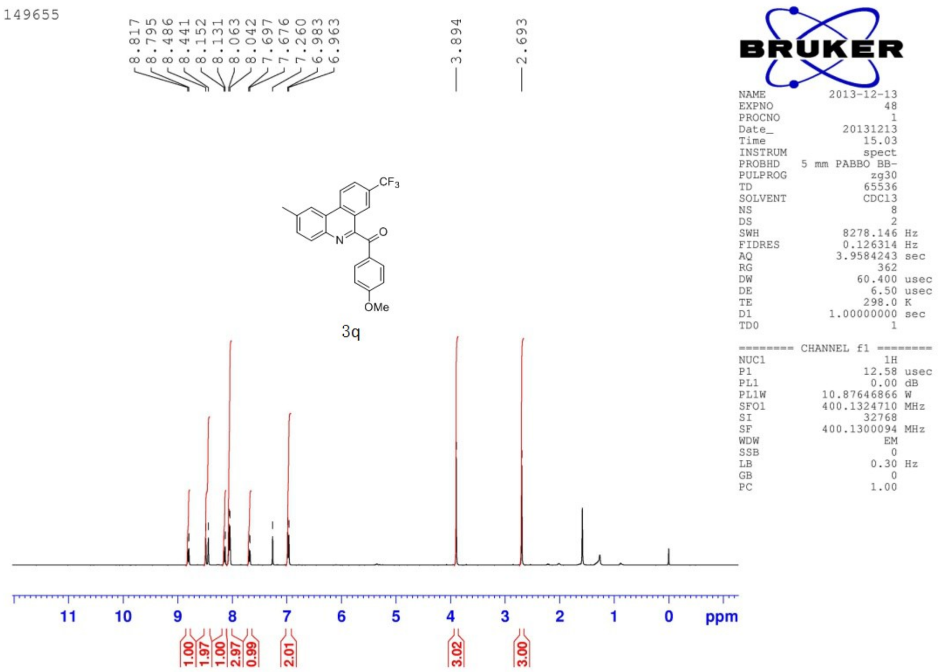
149655
8.817
8.795
8.486
8.441
8.152
8.131
8.063
8.042
7.697
7.676
7.260
6.983
6.963
3.894
2.693
CF3
O
N
OMe
3q
BRUKER
NAME 2013-12-13
EXPNO 48
PROCNO 1
Date_ 20131213
Time 15.03
INSTRUM spect
PROBHD 5 mm PABBO BB-
PULPROG zg30
TD 65536
SOLVENT CDCl3
NS 8
DS 2
SWH 8278.146 Hz
FIDRES 0.126314 Hz
AQ 3.9584243 sec
RG 362
DW 60.400 usec
DE 6.50 usec
TE 298.0 K
D1 1.00000000 sec
TD0 1
======== CHANNEL f1 ========
NUC1 1H
P1 12.58 usec
PL1 0.00 dB
PL1W 10.87646866 W
SFO1 400.1324710 MHz
SI 32768
SF 400.1300094 MHz
WDW EM
SSB 0
LB 0.30 Hz
GB 0
PC 1.00
11 10 9 8 7 6 5 4 3 2 1 0 ppm
1.00 1.97 1.00 2.97 0.99 2.01 3.02 3.00

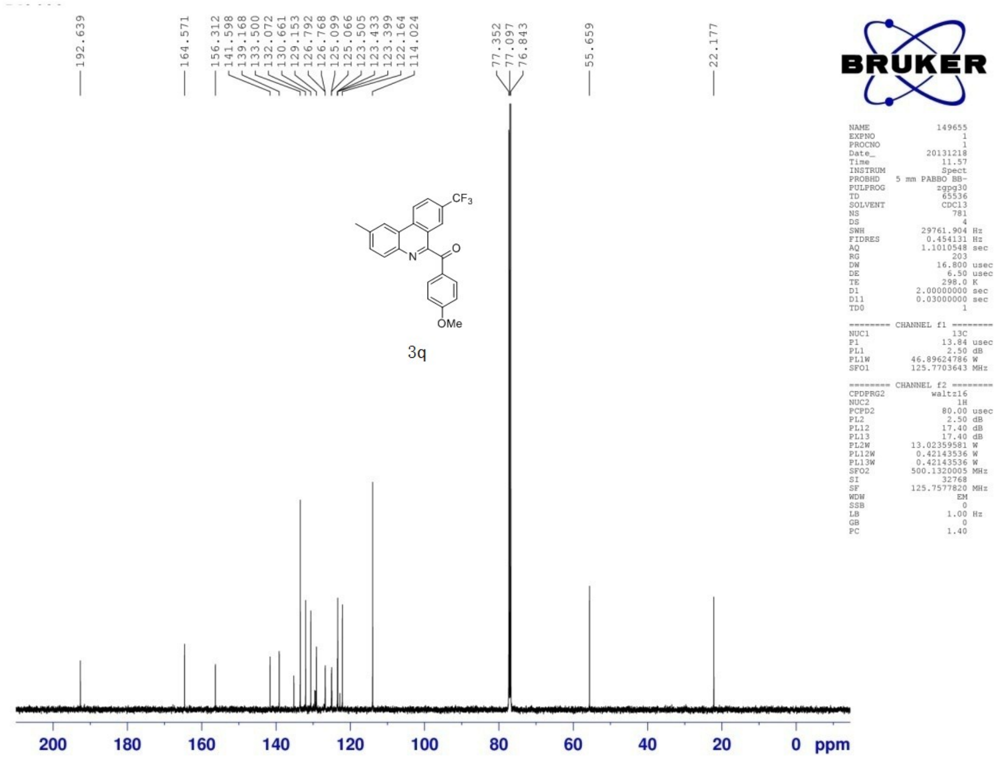
192.639
164.571
156.312
141.598
139.168
133.500
132.072
130.661
129.153
126.792
125.768
125.099
124.066
123.505
123.433
123.399
122.164
114.024
77.352
77.097
76.843
55.659
22.177
CF3
O
N
OMe
3q
BRUKER
NAME 149655
EXPNO 1
PROCNO 1
Date_ 20131218
Time 11.57
INSTRUM spect
PROBHD 5 mm PABBO BB-
PULPROG zgpg30
TD 65536
SOLVENT CDCl3
NS 781
DS 4
SWH 29761.904 Hz
FIDRES 0.454131 Hz
AQ 1.1010548 sec
RG 203
DW 16.800 usec
DE 6.50 usec
TE 298.0 K
D1 2.00000000 sec
D11 0.03000000 sec
TD0 1
======== CHANNEL f1 ========
NUC1 13C
P1 13.84 usec
PL1 2.50 dB
PL1W 46.89624786 W
SFO1 125.7703643 MHz
======== CHANNEL f2 ========
CPDPRG2 waltz16
NUC2 1H
PCPD2 80.00 usec
PL2 2.50 dB
PL12 17.40 dB
PL13 17.40 dB
PL2W 13.02359581 W
PL12W 0.42143536 W
PL13W 0.42143536 W
SFO2 500.1320005 MHz
SI 32768
SF 125.7577820 MHz
WDW EM
SSB 0
LB 1.00 Hz
GB 0
PC 1.40
200 180 160 140 120 100 80 60 40 20 0 ppm

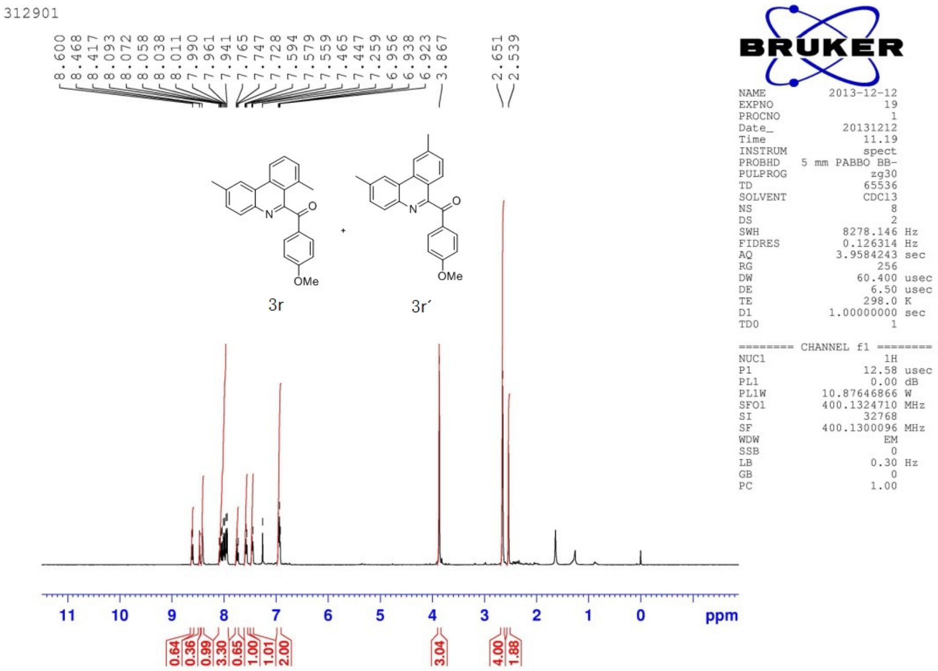
312901
8.600
8.468
8.417
8.093
8.072
8.058
8.038
8.011
7.990
7.961
7.941
7.765
7.747
7.728
7.594
7.579
7.559
7.465
7.447
7.259
6.956
6.938
6.923
3.867
2.651
2.539
BRUKER
NAME 2013-12-12
EXPNO 19
PROCNO 1
Date_ 20131212
Time 11.19
INSTRUM spect
PROBHD 5 mm PABBO BB-
PULPROG zg30
TD 65536
SOLVENT CDCl3
NS 8
DS 2
SWH 8278.146 Hz
FIDRES 0.126314 Hz
AQ 3.9584243 sec
RG 256
DW 60.400 usec
DE 6.50 usec
TE 298.0 K
D1 1.00000000 sec
TD0 1
======== CHANNEL f1 ========
NUC1 1H
P1 12.58 usec
PL1 0.00 dB
PL1W 10.87646866 W
SFO1 400.1324710 MHz
SI 32768
SF 400.1300096 MHz
WDW EM
SSB 0
LB 0.30 Hz
GB 0
PC 1.00
N
O
OMe
+
N
O
OMe
3r
3r´
11
10
9
8
7
6
5
4
3
2
1
0
ppm
0.64
0.36
0.99
3.30
0.65
1.00
1.01
2.00
3.04
4.00
1.88

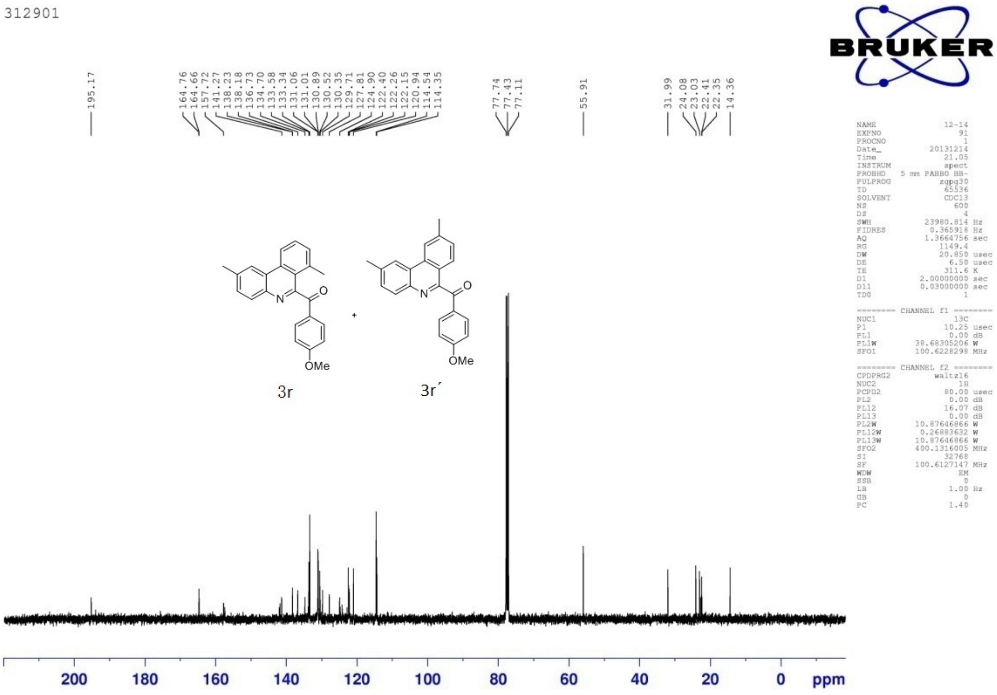
312901
195.17
164.76
164.66
157.72
141.27
138.23
138.18
136.73
134.70
133.58
133.34
131.06
131.01
130.89
130.52
130.25
129.71
127.81
124.90
122.40
122.26
122.15
120.94
114.54
114.35
77.74
77.43
77.11
55.91
31.99
24.08
23.03
22.41
22.35
14.36
BRUKER
NAME 12-14
EXPNO 91
PROCNO 1
Date_ 20131214
Time 21.05
INSTRUM spect
PROBHD 5 mm PABBO BB-
PULPROG zgpg30
TD 65536
SOLVENT CDCl3
NS 600
DS 4
SWH 23980.814 Hz
FIDRES 0.365918 Hz
AQ 1.3664756 sec
RG 1149.4
DW 20.850 usec
DE 6.50 usec
TE 311.6 K
D1 2.00000000 sec
D11 0.03000000 sec
TD0 1
======== CHANNEL f1 ========
NUC1 13C
P1 10.25 usec
PL1 0.00 dB
PL1W 38.68305206 W
SFO1 100.6228298 MHz
======== CHANNEL f2 ========
CPDPRG2 waltz16
NUC2 1H
PCPD2 80.00 usec
PL2 0.00 dB
PL12 16.07 dB
PL13 0.00 dB
PL2W 10.87646866 W
PL12W 0.26883632 W
PL13W 10.87646866 W
SFO2 400.1316005 MHz
SI 32768
SF 100.6127147 MHz
WDW EM
SSB 0
LB 1.00 Hz
GB 0
PC 1.40
N
O
OMe
+
N
O
OMe
3r
3r´
200
180
160
140
120
100
80
60
40
20
0
ppm

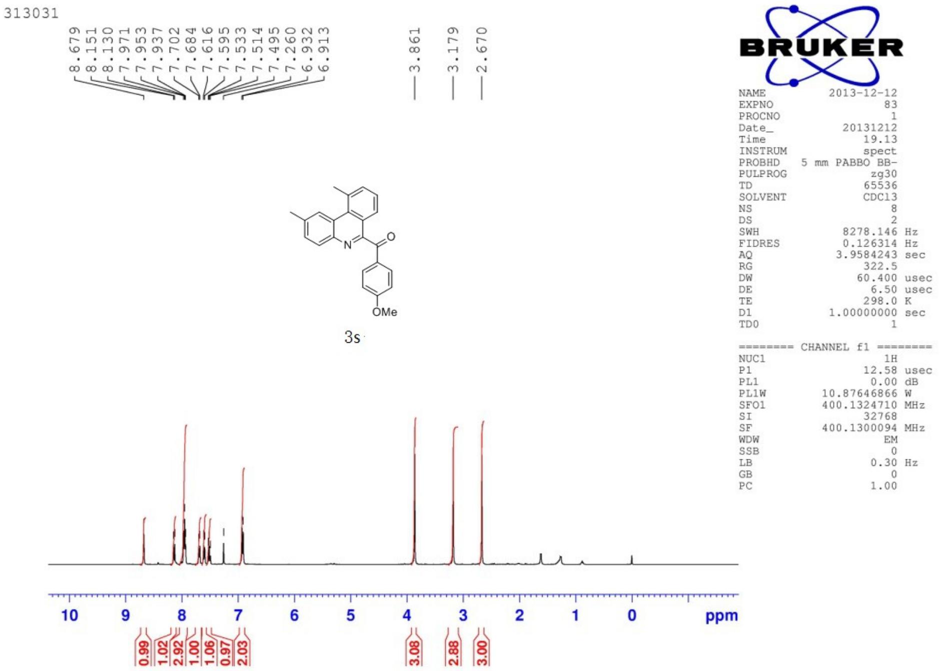
313031
8.679
8.151
8.130
7.971
7.953
7.937
7.702
7.684
7.616
7.595
7.533
7.514
7.495
7.260
6.932
6.913
3.861
3.179
2.670
BRUKER
NAME 2013-12-12
EXPNO 83
PROCNO 1
Date_ 20131212
Time 19.13
INSTRUM spect
PROBHD 5 mm PABBO BB-
PULPROG zg30
TD 65536
SOLVENT CDCl3
NS 8
DS 2
SWH 8278.146 Hz
FIDRES 0.126314 Hz
AQ 3.9584243 sec
RG 322.5
DW 60.400 usec
DE 6.50 usec
TE 298.0 K
D1 1.00000000 sec
TD0 1
======== CHANNEL f1 ========
NUC1 1H
P1 12.58 usec
PL1 0.00 dB
PL1W 10.87646866 W
SFO1 400.1324710 MHz
SI 32768
SF 400.1300094 MHz
WDW EM
SSB 0
LB 0.30 Hz
GB 0
PC 1.00
N
O
OMe
3s
10
9
8
7
6
5
4
3
2
1
0
ppm
0.99
1.02
2.92
1.00
1.06
0.97
2.03
3.08
2.88
3.00

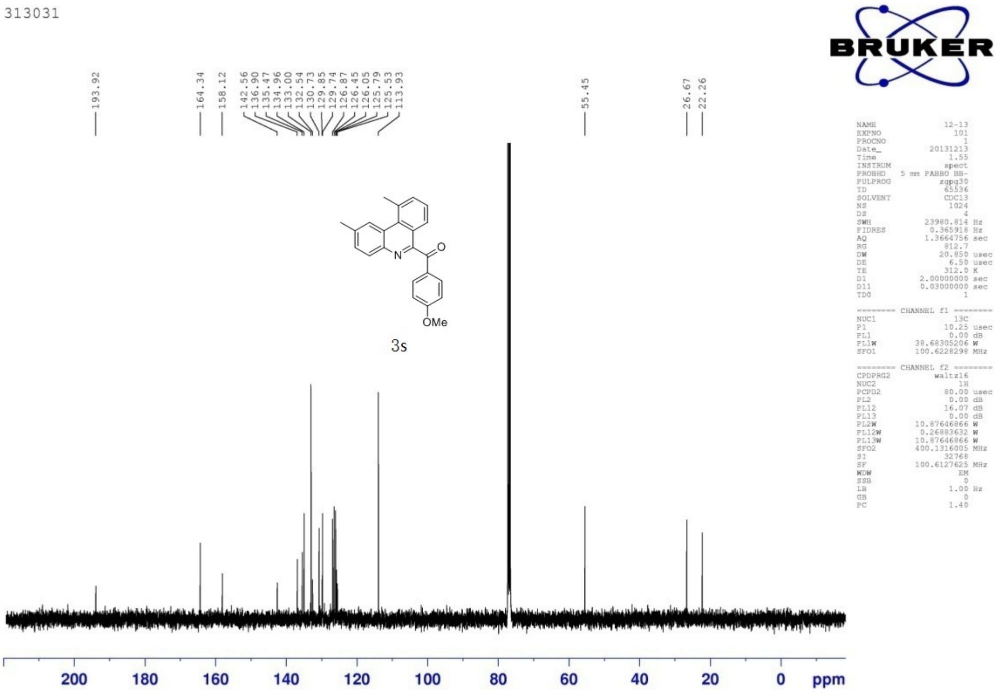
313031
193.92
164.34
158.12
142.56
136.90
135.47
134.96
133.00
132.54
130.73
129.85
129.74
126.87
126.45
126.05
125.79
125.53
113.93
55.45
26.67
22.26
BRUKER
N
O
OMe
3s
200
180
160
140
120
100
80
60
40
20
0
ppm

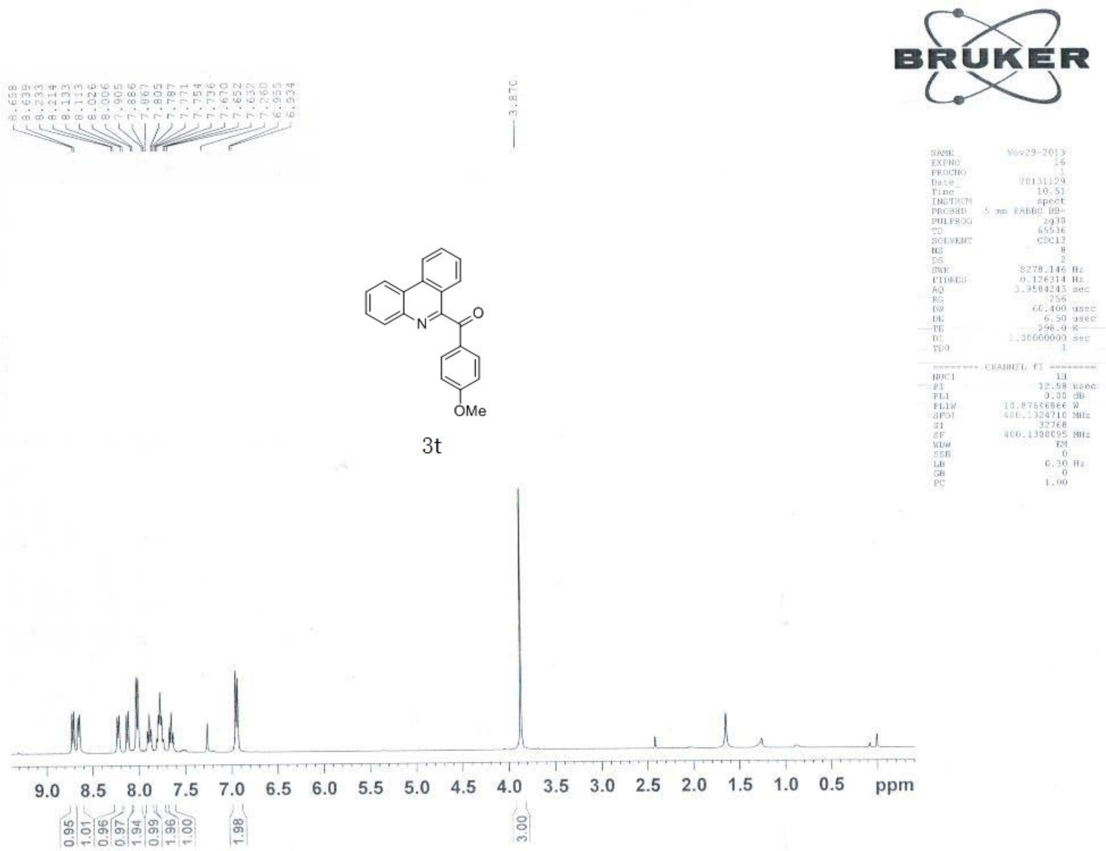
8.658 8.639 8.233 8.214 8.133 8.113 8.026 8.006 7.905 7.886 7.867 7.805 7.787 7.771 7.754 7.736 7.670 7.652 7.632 7.260 6.955 6.934
3.870
BRUKER
OMe
3t
9.0 8.5 8.0 7.5 7.0 6.5 6.0 5.5 5.0 4.5 4.0 3.5 3.0 2.5 2.0 1.5 1.0 0.5 ppm
0.95 1.01 0.96 0.97 1.94 0.99 1.96 1.00 1.98 3.00

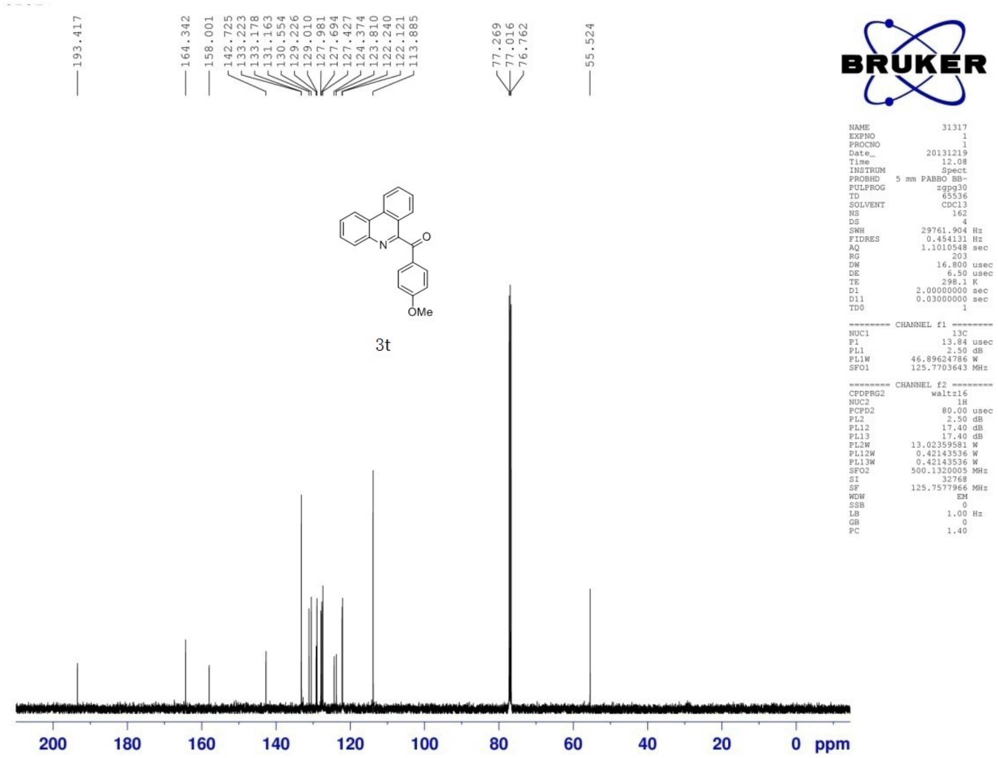
193.417
164.342 158.001 142.725 133.223 131.178 131.163 130.554 129.226 129.010 127.981 127.694 127.427 124.374 123.810 122.240 122.121 113.885
77.269 77.016 76.762
55.524
BRUKER
OMe
3t
200 180 160 140 120 100 80 60 40 20 0 ppm

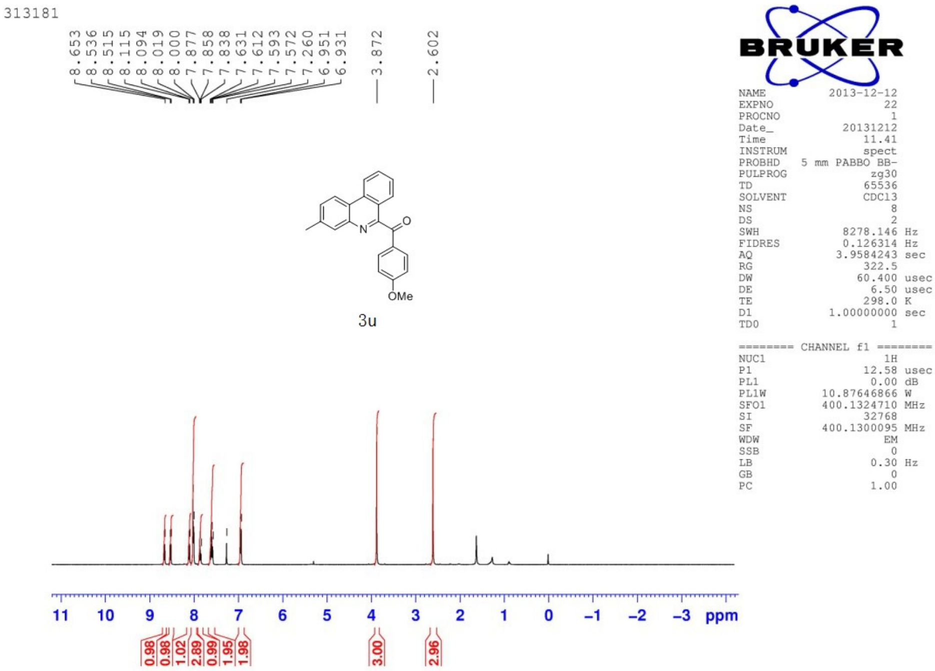
313181
8.653 8.536 8.515 8.115 8.094 8.019 8.000 7.877 7.858 7.838 7.631 7.612 7.593 7.572 7.260 6.951 6.931
3.872
2.602
N
O
OMe
3u
NAME 2013-12-12
EXPNO 22
PROCNO 1
Date_ 20131212
Time 11.41
INSTRUM spect
PROBHD 5 mm PABBO BB-
PULPROG zg30
TD 65536
SOLVENT CDCl3
NS 8
DS 2
SWH 8278.146 Hz
FIDRES 0.126314 Hz
AQ 3.9584243 sec
RG 322.5
DW 60.400 usec
DE 6.50 usec
TE 298.0 K
D1 1.00000000 sec
TD0 1
======== CHANNEL f1 ========
NUC1 1H
P1 12.58 usec
PL1 0.00 dB
PL1W 10.87646866 W
SFO1 400.1324710 MHz
SI 32768
SF 400.1300095 MHz
WDW EM
SSB 0
LB 0.30 Hz
GB 0
PC 1.00
11 10 9 8 7 6 5 4 3 2 1 0 −1 −2 −3 ppm
0.98 0.98 1.02 2.89 0.99 1.95 1.98 3.00 2.96

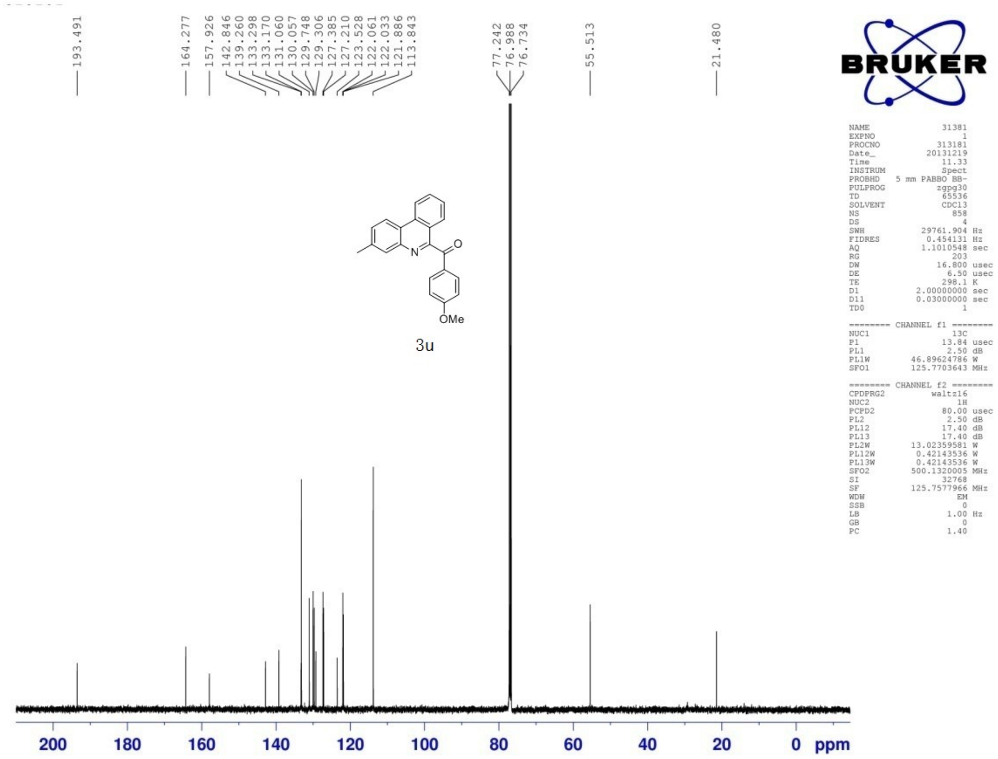
193.491
164.277 157.926 142.846 139.260 133.298 133.170 131.060 130.057 129.748 129.306 127.385 127.210 123.528 122.061 122.033 121.886 113.843
77.242 76.988 76.734
55.513
21.480
N
O
OMe
3u
NAME 31381
EXPNO 1
PROCNO 313181
Date_ 20131219
Time 11.33
INSTRUM Spect
PROBHD 5 mm PABBO BB-
PULPROG zgpg30
TD 65536
SOLVENT CDCl3
NS 858
DS 4
SWH 29761.904 Hz
FIDRES 0.454131 Hz
AQ 1.1010548 sec
RG 203
DW 16.800 usec
DE 6.50 usec
TE 298.1 K
D1 2.00000000 sec
D11 0.03000000 sec
TD0 1
======== CHANNEL f1 ========
NUC1 13C
P1 13.84 usec
PL1 2.50 dB
PL1W 46.89624786 W
SFO1 125.7703643 MHz
======== CHANNEL f2 ========
CPDPRG2 waltz16
NUC2 1H
PCPD2 80.00 usec
PL2 2.50 dB
PL12 17.40 dB
PL13 17.40 dB
PL2W 13.02359581 W
PL12W 0.42143536 W
PL13W 0.42143536 W
SFO2 500.1320005 MHz
SI 32768
SF 125.7577966 MHz
WDW EM
SSB 0
LB 1.00 Hz
GB 0
PC 1.40
200 180 160 140 120 100 80 60 40 20 0 ppm

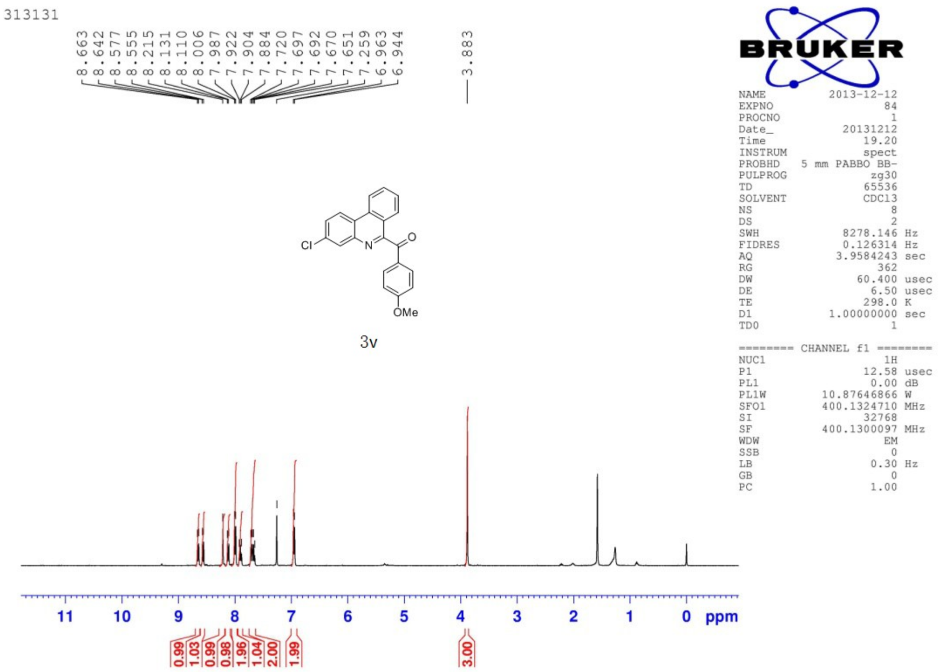
313131
8.663
8.642
8.577
8.555
8.215
8.131
8.110
8.006
7.987
7.922
7.904
7.884
7.720
7.697
7.692
7.670
7.651
7.259
6.963
6.944
3.883
BRUKER
NAME 2013-12-12
EXPNO 84
PROCNO 1
Date_ 20131212
Time 19.20
INSTRUM spect
PROBHD 5 mm PABBO BB-
PULPROG zg30
TD 65536
SOLVENT CDCl3
NS 8
DS 2
SWH 8278.146 Hz
FIDRES 0.126314 Hz
AQ 3.9584243 sec
RG 362
DW 60.400 usec
DE 6.50 usec
TE 298.0 K
D1 1.00000000 sec
TD0 1
======== CHANNEL f1 ========
NUC1 1H
P1 12.58 usec
PL1 0.00 dB
PL1W 10.87646866 W
SFO1 400.1324710 MHz
SI 32768
SF 400.1300097 MHz
WDW EM
SSB 0
LB 0.30 Hz
GB 0
PC 1.00
Cl
N
O
OMe
3v
11 10 9 8 7 6 5 4 3 2 1 0 ppm
0.99
1.03
0.99
0.98
1.96
1.04
2.00
1.99
3.00

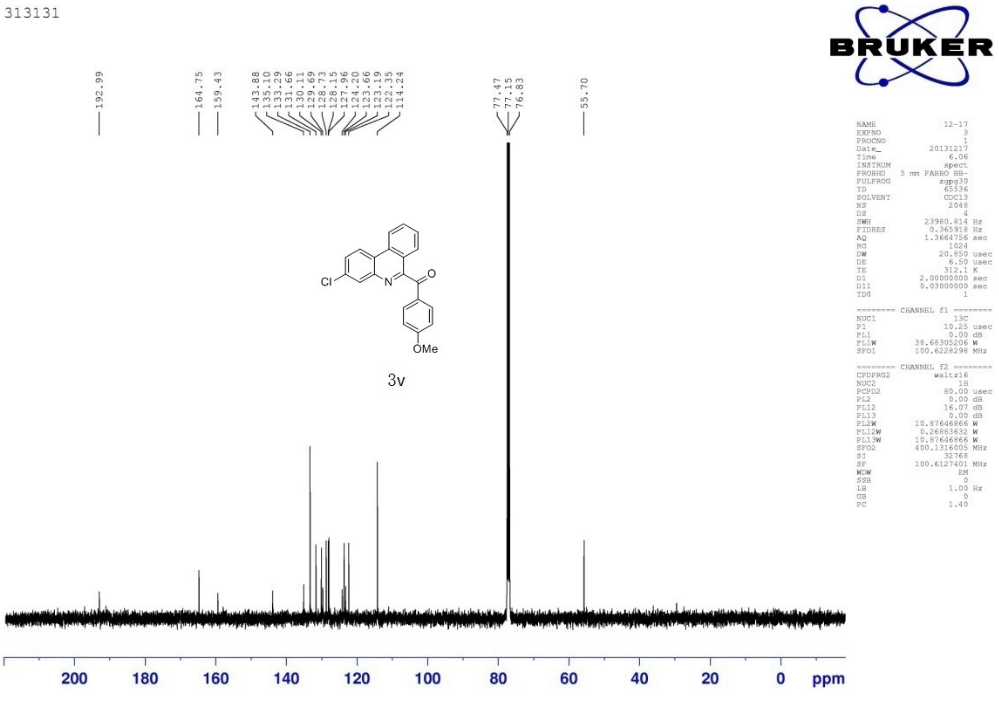
313131
192.99
164.75
159.43
143.88
135.10
133.29
131.66
130.11
129.69
128.73
128.15
127.96
124.20
123.66
123.19
122.35
114.24
77.47
77.15
76.83
55.70
BRUKER
Cl
N
O
OMe
3v
200 180 160 140 120 100 80 60 40 20 0 ppm

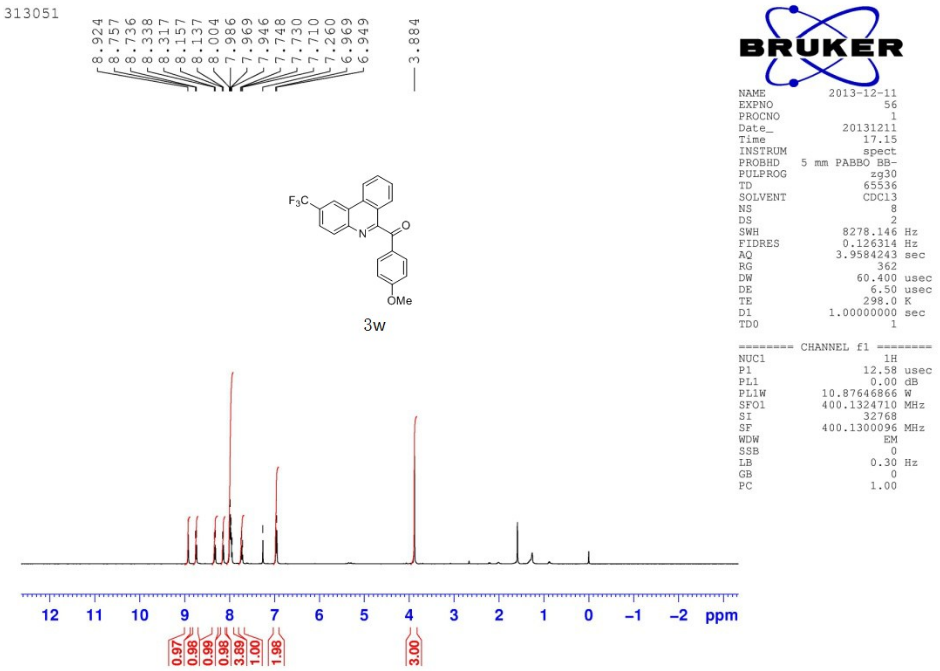
313051
8.924
8.757
8.736
8.338
8.317
8.157
8.137
8.004
7.986
7.969
7.946
7.748
7.730
7.710
7.260
6.969
6.949
3.884
F_3C
N
O
OMe
3w
BRUKER
NAME 2013-12-11
EXPNO 56
PROCNO 1
Date_ 20131211
Time 17.15
INSTRUM spect
PROBHD 5 mm PABBO BB-
PULPROG zg30
TD 65536
SOLVENT CDCl3
NS 8
DS 2
SWH 8278.146 Hz
FIDRES 0.126314 Hz
AQ 3.9584243 sec
RG 362
DW 60.400 usec
DE 6.50 usec
TE 298.0 K
D1 1.00000000 sec
TD0 1
======== CHANNEL f1 ========
NUC1 1H
P1 12.58 usec
PL1 0.00 dB
PL1W 10.87646866 W
SFO1 400.1324710 MHz
SI 32768
SF 400.1300096 MHz
WDW EM
SSB 0
LB 0.30 Hz
GB 0
PC 1.00
12 11 10 9 8 7 6 5 4 3 2 1 0 −1 −2 ppm
0.97 0.98 0.99 0.98 3.89 1.00 1.98 3.00

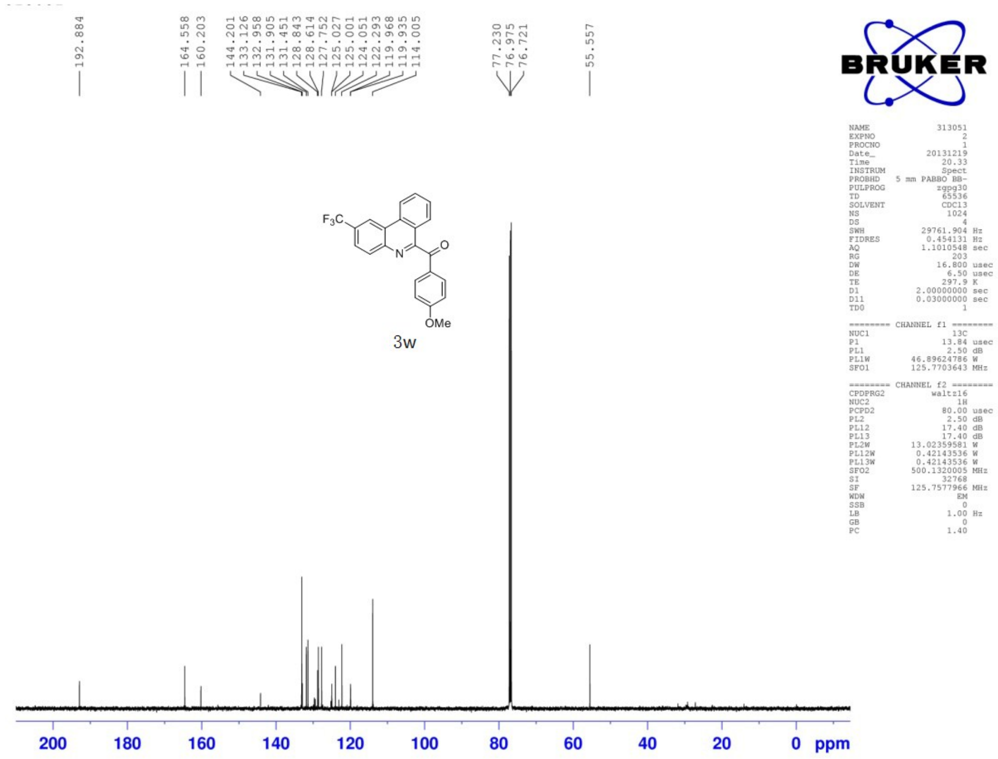
192.884
164.558
160.203
144.201
133.126
132.958
131.905
131.451
128.843
128.614
127.752
125.027
125.001
124.051
122.293
119.968
119.935
114.005
77.230
76.975
76.721
55.557
F_3C
N
O
OMe
3w
BRUKER
NAME 313051
EXPNO 2
PROCNO 1
Date_ 20131219
Time 20.33
INSTRUM Spect
PROBHD 5 mm PABBO BB-
PULPROG zgpg30
TD 65536
SOLVENT CDCl3
NS 1024
DS 4
SWH 29761.904 Hz
FIDRES 0.454131 Hz
AQ 1.1010548 sec
RG 203
DW 16.800 usec
DE 6.50 usec
TE 297.9 K
D1 2.00000000 sec
D11 0.03000000 sec
TD0 1
======== CHANNEL f1 ========
NUC1 13C
P1 13.84 usec
PL1 2.50 dB
PL1W 46.89624786 W
SFO1 125.7703643 MHz
======== CHANNEL f2 ========
CPDPRG2 waltz16
NUC2 1H
PCPD2 80.00 usec
PL2 2.50 dB
PL12 17.40 dB
PL13 17.40 dB
PL2W 13.02359581 W
PL12W 0.42143536 W
PL13W 0.42143536 W
SFO2 500.1320005 MHz
SI 32768
SF 125.7577966 MHz
WDW EM
SSB 0
LB 1.00 Hz
GB 0
PC 1.40
200 180 160 140 120 100 80 60 40 20 0 ppm

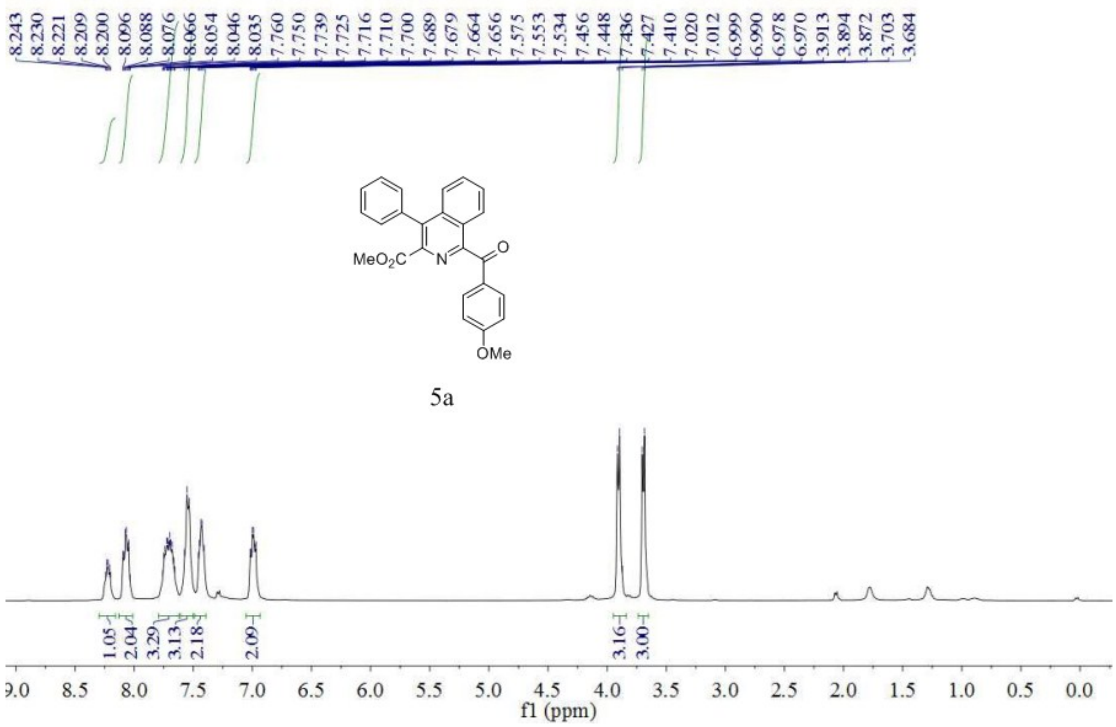
8.243 8.230 8.221 8.209 8.200 8.096 8.088 8.076 8.066 8.054 8.046 8.035 7.760 7.750 7.739 7.725 7.716 7.710 7.700 7.689 7.679 7.664 7.656 7.575 7.553 7.534 7.456 7.448 7.436 7.427 7.410 7.020 7.012 6.999 6.990 6.978 6.970 3.913 3.894 3.872 3.703 3.684
MeO2C
N
O
OMe
5a
1.05
2.04
3.29
3.13
2.18
2.09
3.16
3.00
f1 (ppm)

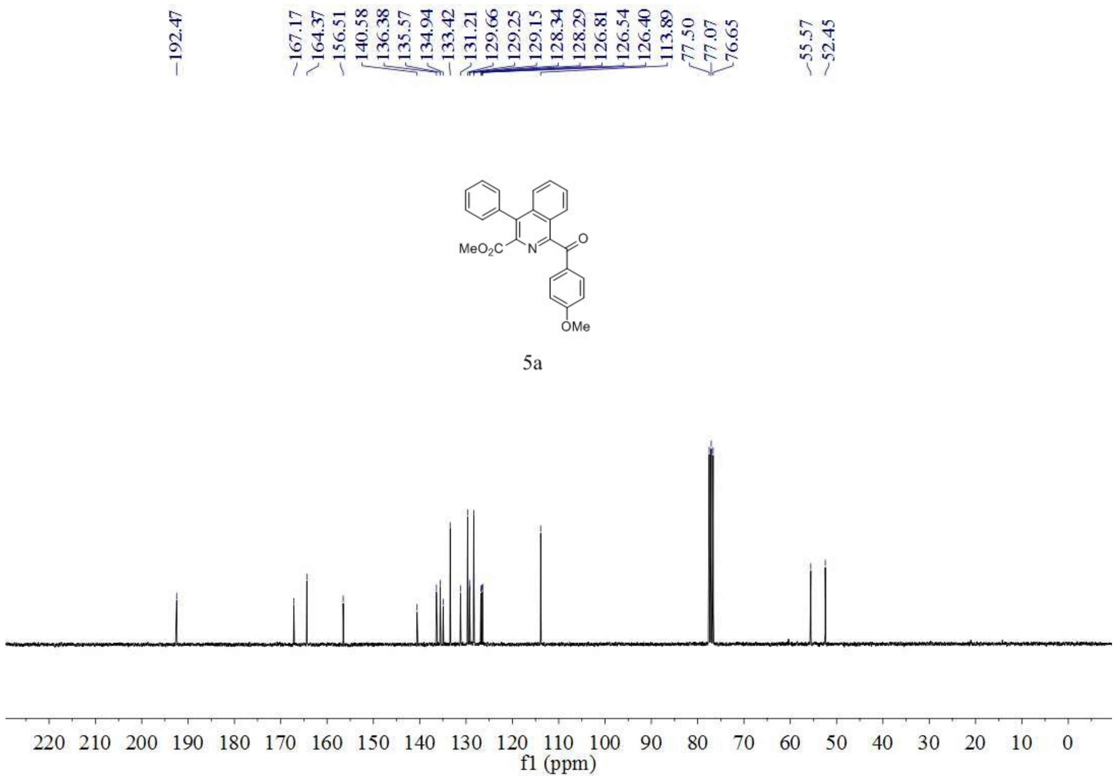
192.47
167.17 164.37 156.51 140.58 136.38 135.57 134.94 133.42 131.21 129.66 129.25 129.15 128.34 128.29 126.81 126.54 126.40 113.89 77.50 77.07 76.65
55.57 52.45
MeO2C
N
O
OMe
5a
f1 (ppm)

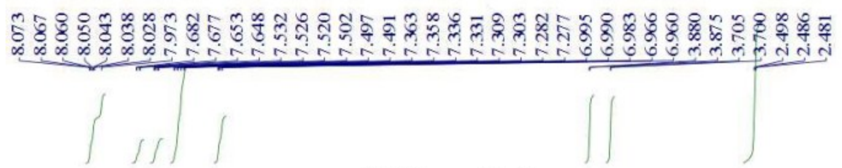
8.073
8.067
8.060
8.050
8.043
8.038
8.028
7.973
7.682
7.677
7.653
7.648
7.532
7.526
7.520
7.502
7.497
7.491
7.363
7.358
7.336
7.331
7.309
7.303
7.282
7.277
6.995
6.990
6.983
6.966
6.960
3.880
3.875
3.705
3.700
2.498
2.486
2.481

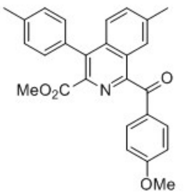
MeO2C
N
O
OMe

5b

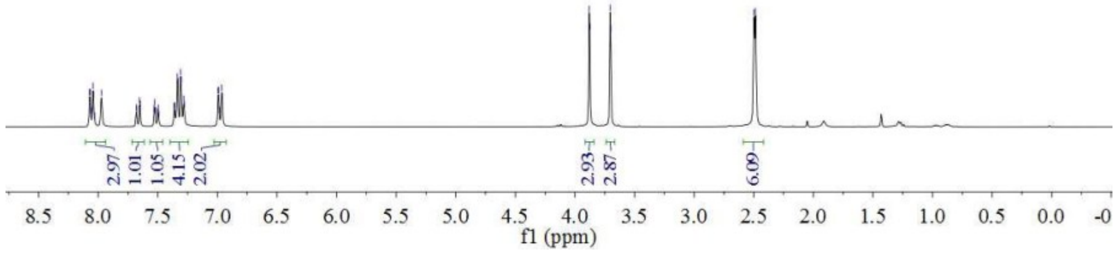
2.97
1.01
1.05
4.15
2.02
2.93
2.87
6.09
8.5
8.0
7.5
7.0
6.5
6.0
5.5
5.0
4.5
4.0
3.5
3.0
2.5
2.0
1.5
1.0
0.5
0.0
-0
f1 (ppm)

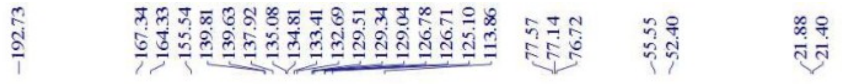
192.73
167.34
164.33
155.54
139.81
139.63
137.92
135.08
134.81
133.41
132.69
129.51
129.34
129.04
126.78
126.71
125.10
113.86
77.57
77.14
76.72
55.55
52.40
21.88
21.40

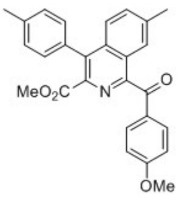
MeO2C
N
O
OMe

5b

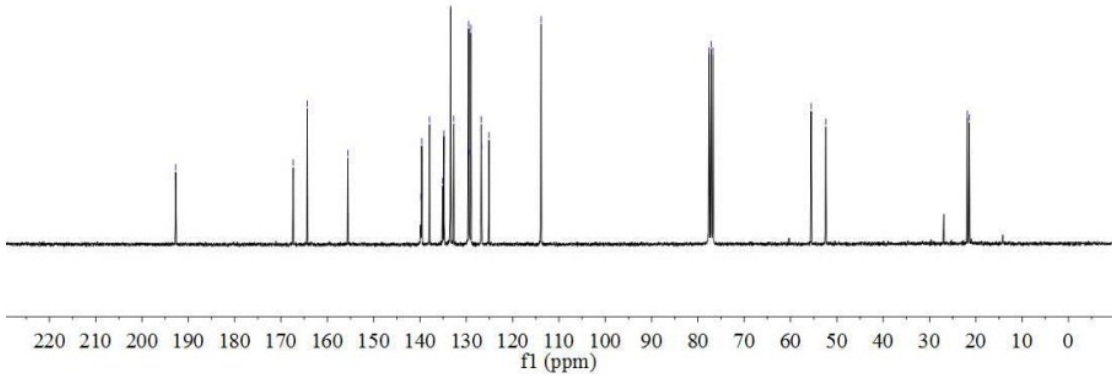
220
210
200
190
180
170
160
150
140
130
120
110
100
90
80
70
60
50
40
30
20
10
0
f1 (ppm)

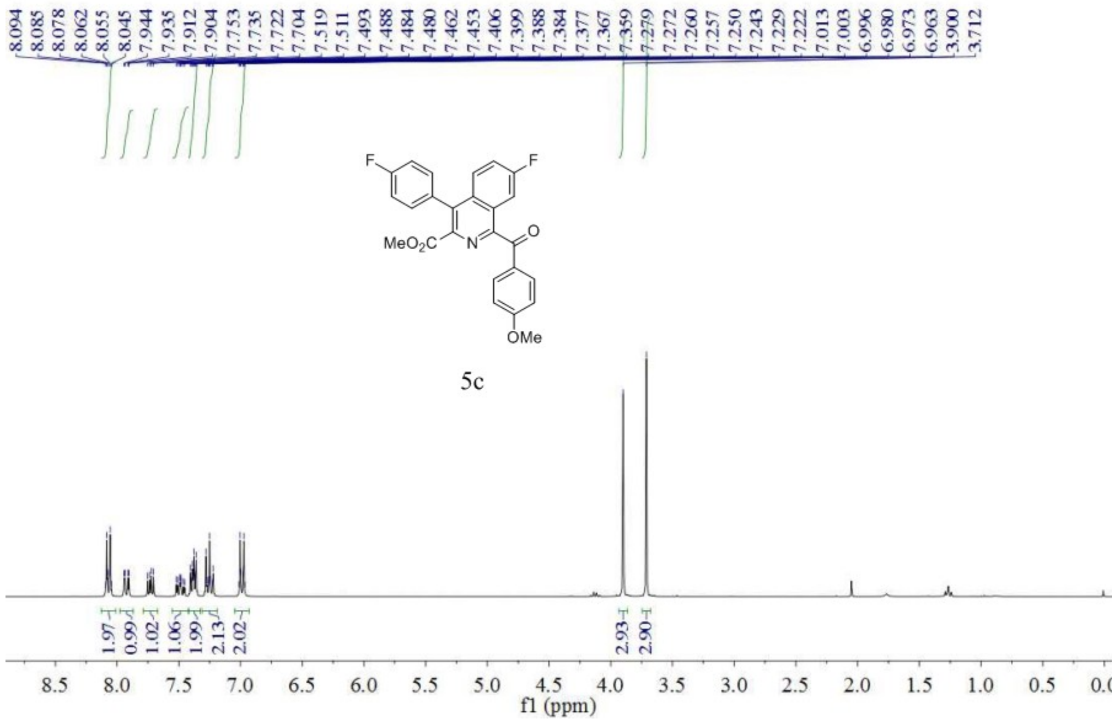
8.094
8.085
8.078
8.062
8.055
8.045
7.944
7.935
7.912
7.904
7.753
7.735
7.722
7.704
7.519
7.511
7.493
7.488
7.484
7.480
7.462
7.453
7.406
7.399
7.388
7.384
7.377
7.367
7.359
7.279
7.272
7.260
7.257
7.250
7.243
7.229
7.222
7.013
7.003
6.996
6.980
6.973
6.963
3.900
3.712
F
F
MeO2C
N
O
OMe
5c
1.97
0.99
1.02
1.06
1.99
2.13
2.02
2.93
2.90
8.5 8.0 7.5 7.0 6.5 6.0 5.5 5.0 4.5 4.0 3.5 3.0 2.5 2.0 1.5 1.0 0.5
f1 (ppm)

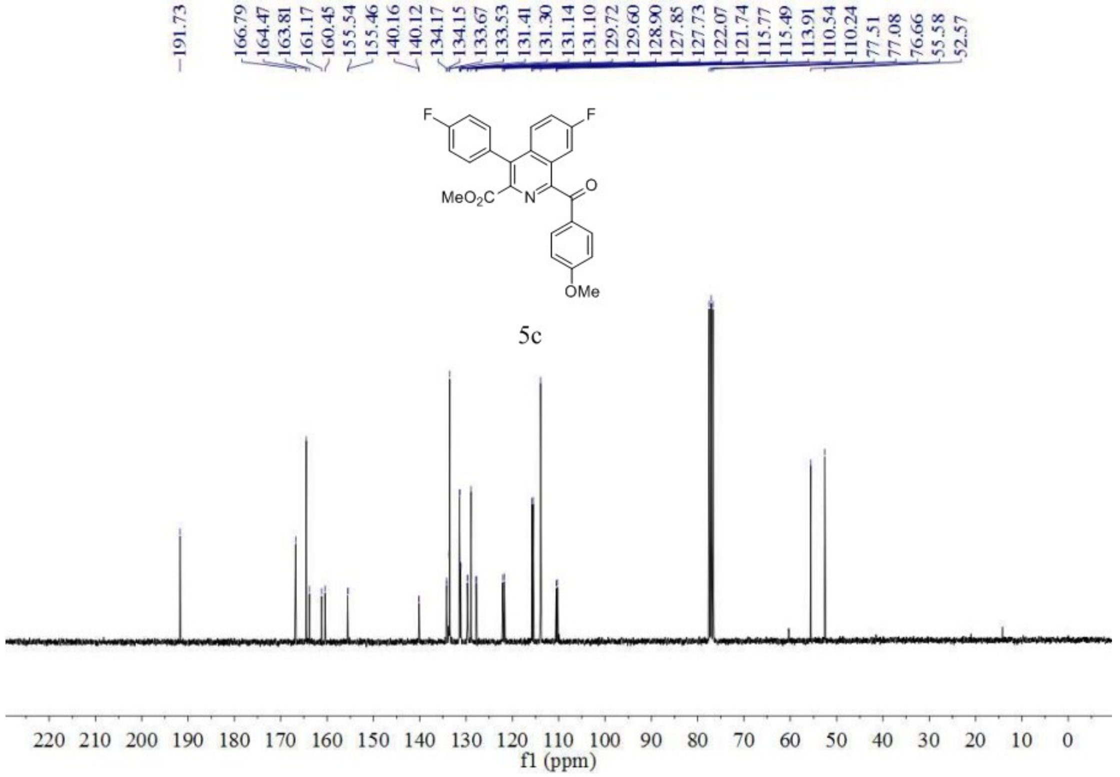
191.73
166.79
164.47
163.81
161.17
160.45
155.54
155.46
140.16
140.12
134.17
134.15
133.67
133.53
131.41
131.30
131.14
131.10
129.72
129.60
128.90
127.85
127.73
122.07
121.74
115.77
115.49
113.91
110.54
110.24
77.51
77.08
76.66
55.58
52.57
F
F
MeO2C
N
O
OMe
5c
220 210 200 190 180 170 160 150 140 130 120 110 100 90 80 70 60 50 40 30 20 10 0
f1 (ppm)

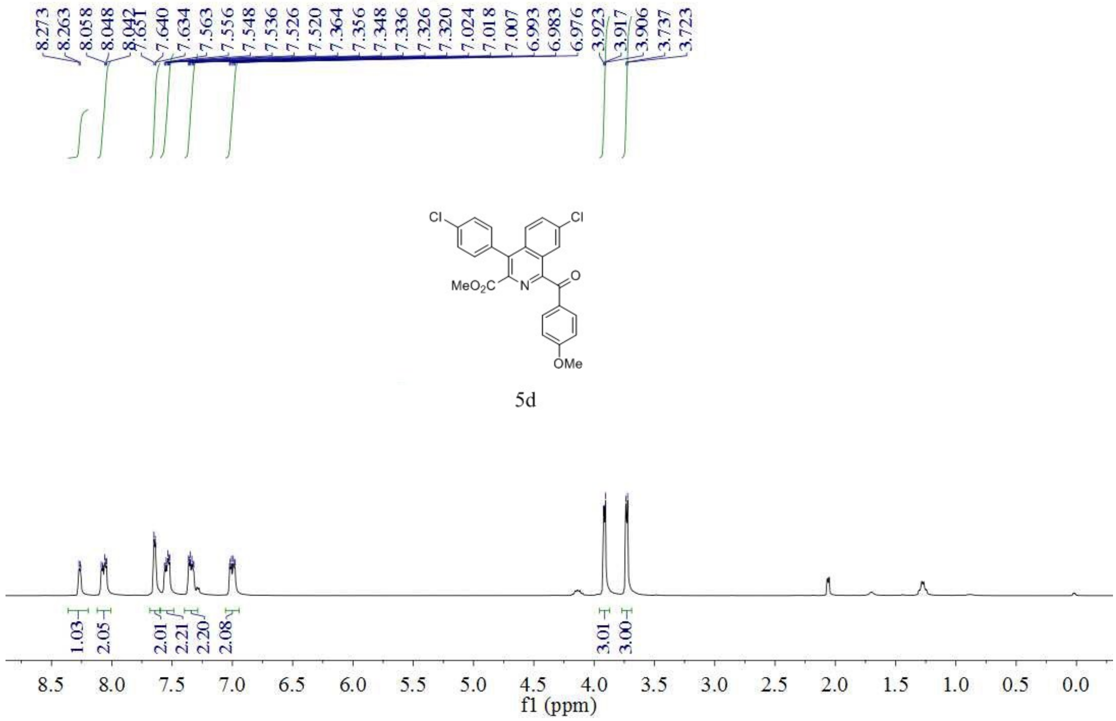
8.273
8.263
8.058
8.048
8.042
7.651
7.640
7.634
7.563
7.556
7.548
7.536
7.526
7.520
7.364
7.356
7.348
7.336
7.326
7.320
7.024
7.018
7.007
6.993
6.983
6.976
3.923
3.917
3.906
3.737
3.723
Cl
Cl
MeO$_2$C
N
O
OMe
1.03
2.05
2.01
2.21
2.20
2.08
3.01
3.00
8.5 8.0 7.5 7.0 6.5 6.0 5.5 5.0 4.5 4.0 3.5 3.0 2.5 2.0 1.5 1.0 0.5 0.0
f1 (ppm)

5d

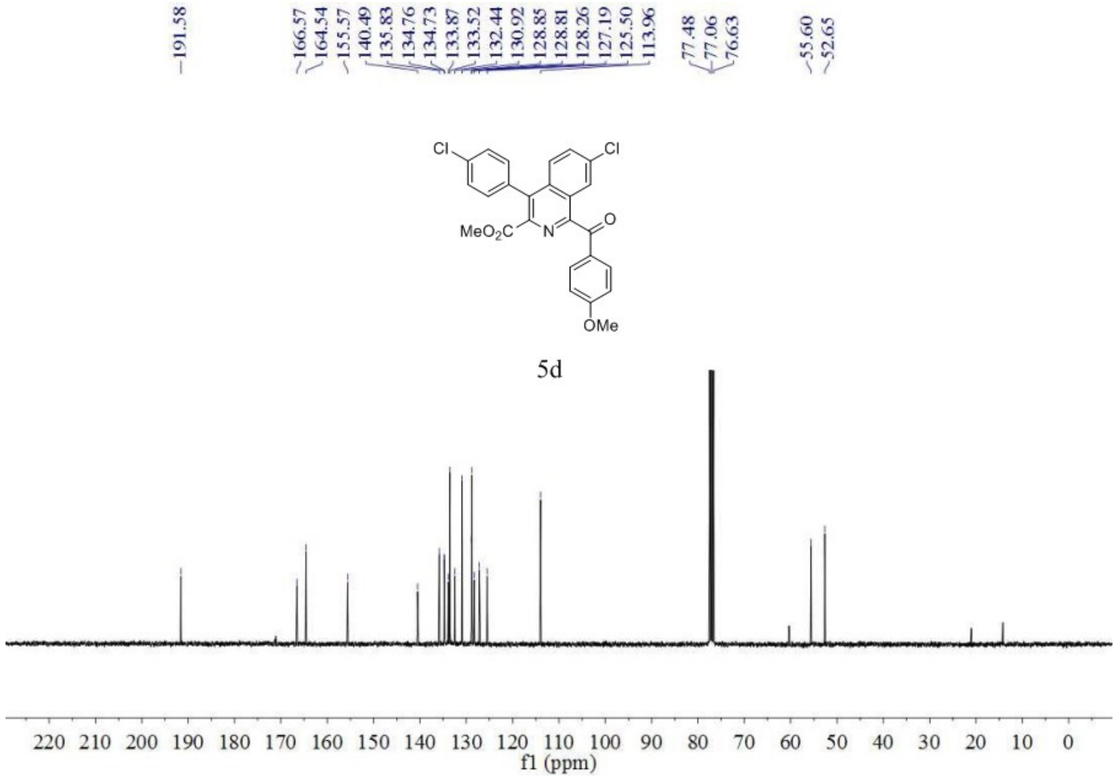
191.58
166.57
164.54
155.57
140.49
135.83
134.76
134.73
133.87
133.52
132.44
130.92
128.85
128.81
128.26
127.19
125.50
113.96
77.48
77.06
76.63
55.60
52.65
Cl
Cl
MeO$_2$C
N
O
OMe
220 210 200 190 180 170 160 150 140 130 120 110 100 90 80 70 60 50 40 30 20 10 0
f1 (ppm)

5d

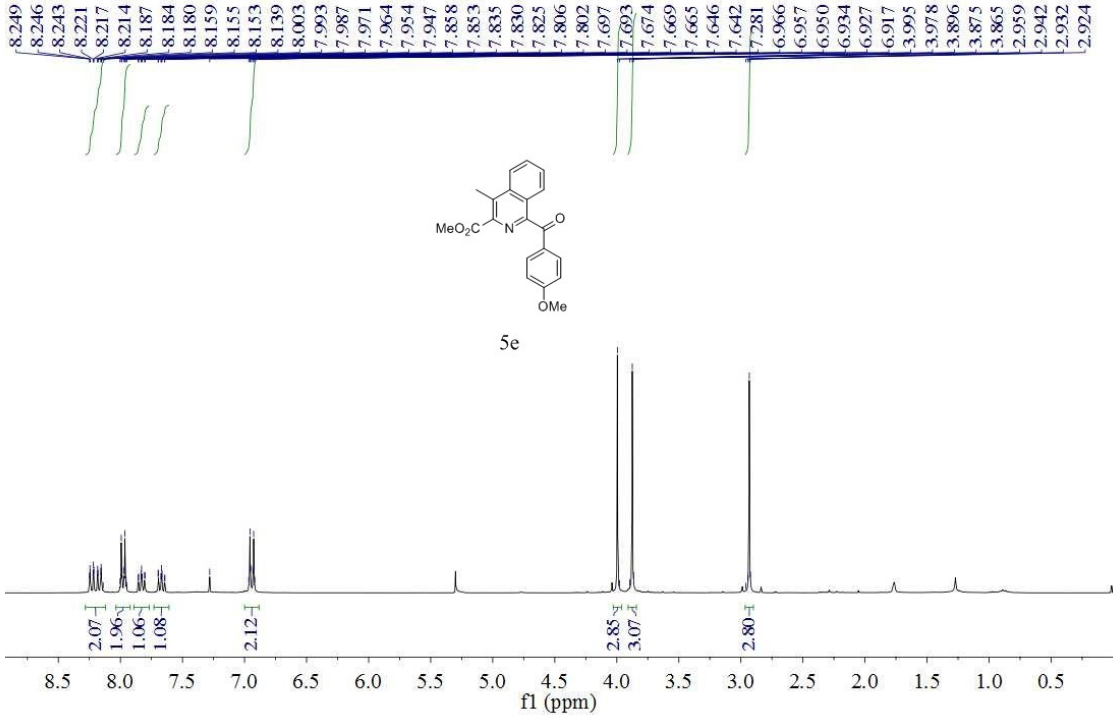
8.249 8.246 8.243 8.221 8.217 8.214 8.187 8.184 8.180 8.159 8.155 8.153 8.139 8.003 7.993 7.987 7.971 7.964 7.954 7.947 7.858 7.853 7.835 7.830 7.825 7.806 7.802 7.697 7.693 7.674 7.669 7.665 7.646 7.642 7.281 6.966 6.957 6.950 6.934 6.927 6.917 3.995 3.978 3.896 3.875 3.865 2.959 2.942 2.932 2.924
MeO2C
N
O
OMe
5e
2.07 1.96 1.06 1.08 2.12 2.85 3.07 2.80
8.5 8.0 7.5 7.0 6.5 6.0 5.5 5.0 4.5 4.0 3.5 3.0 2.5 2.0 1.5 1.0 0.5
f1 (ppm)

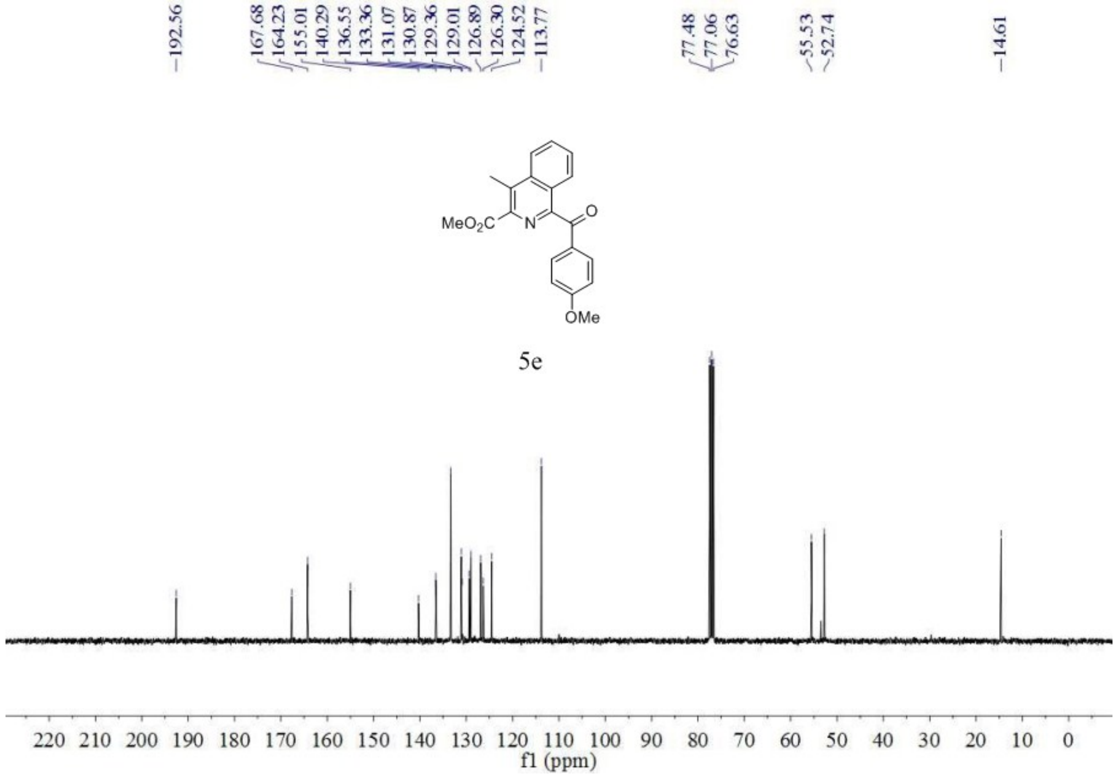
192.56
167.68 164.23 155.01 140.29 136.55 133.36 131.07 130.87 129.36 129.01 126.89 126.30 124.52 113.77
77.48 77.06 76.63
55.53 52.74
14.61
MeO2C
N
O
OMe
5e
220 210 200 190 180 170 160 150 140 130 120 110 100 90 80 70 60 50 40 30 20 10 0
f1 (ppm)

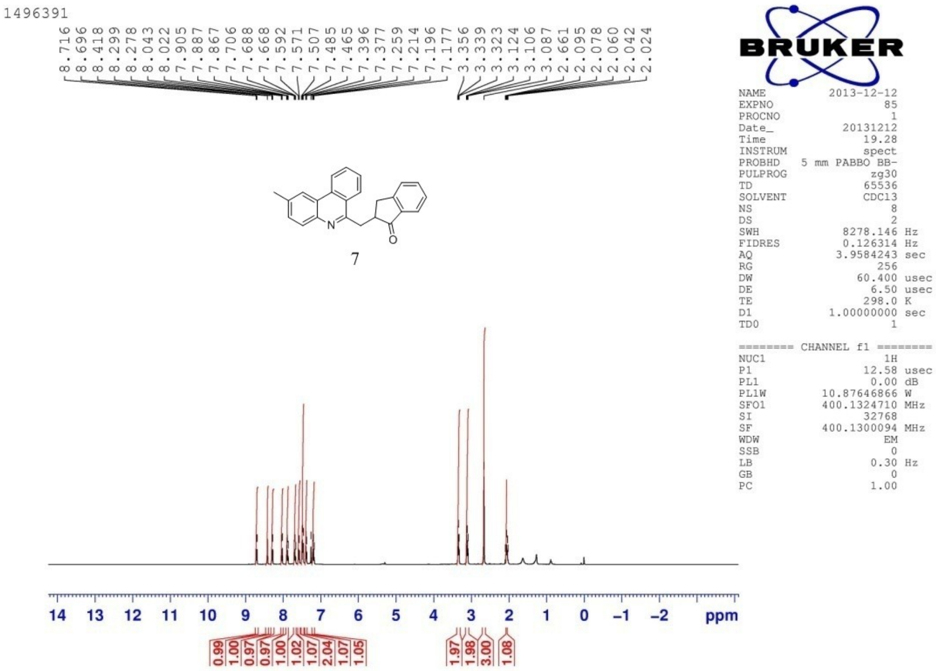
1496391
8.716 8.696 8.418 8.299 8.278 8.043 8.022 7.905 7.887 7.867 7.706 7.688 7.668 7.592 7.571 7.507 7.485 7.465 7.396 7.377 7.259 7.214 7.196 7.177 3.356 3.339 3.323 3.124 3.106 3.087 2.661 2.095 2.078 2.060 2.042 2.024
N
O
7
NAME 2013-12-12
EXPNO 85
PROCNO 1
Date_ 20131212
Time 19.28
INSTRUM spect
PROBHD 5 mm PABBO BB-
PULPROG zg30
TD 65536
SOLVENT CDCl3
NS 8
DS 2
SWH 8278.146 Hz
FIDRES 0.126314 Hz
AQ 3.9584243 sec
RG 256
DW 60.400 usec
DE 6.50 usec
TE 298.0 K
D1 1.00000000 sec
TD0 1
======== CHANNEL f1 ========
NUC1 1H
P1 12.58 usec
PL1 0.00 dB
PL1W 10.87646866 W
SFO1 400.1324710 MHz
SI 32768
SF 400.1300094 MHz
WDW EM
SSB 0
LB 0.30 Hz
GB 0
PC 1.00
14 13 12 11 10 9 8 7 6 5 4 3 2 1 0 −1 −2 ppm
0.99 1.00 0.97 0.97 1.00 1.02 1.07 2.04 1.07 1.05 1.97 1.98 3.00 1.08

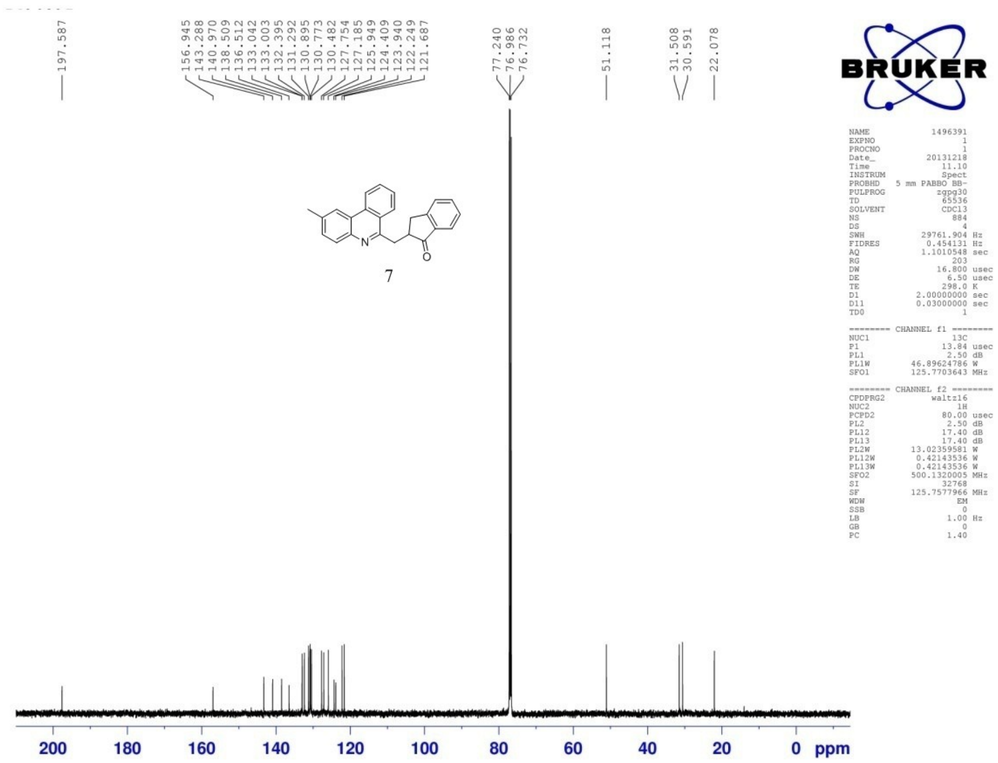
197.587
156.945 143.288 140.970 138.509 136.512 133.042 133.003 132.395 131.292 130.895 130.773 130.482 127.754 127.185 125.949 124.409 123.940 122.249 121.687
77.240 76.986 76.732
51.118
31.508 30.591
22.078
N
O
7
NAME 1496391
EXPNO 1
PROCNO 1
Date_ 20131218
Time 11.10
INSTRUM Spect
PROBHD 5 mm PABBO BB-
PULPROG zgpg30
TD 65536
SOLVENT CDCl3
NS 884
DS 4
SWH 29761.904 Hz
FIDRES 0.454131 Hz
AQ 1.1010548 sec
RG 203
DW 16.800 usec
DE 6.50 usec
TE 298.0 K
D1 2.00000000 sec
D11 0.03000000 sec
TD0 1
======== CHANNEL f1 ========
NUC1 13C
P1 13.84 usec
PL1 2.50 dB
PL1W 46.89624786 W
SFO1 125.7703643 MHz
======== CHANNEL f2 ========
CPDPRG2 waltz16
NUC2 1H
PCPD2 80.00 usec
PL2 2.50 dB
PL12 17.40 dB
PL13 17.40 dB
PL2W 13.02359581 W
PL12W 0.42143536 W
PL13W 0.42143536 W
SFO2 500.1320005 MHz
SI 32768
SF 125.7577966 MHz
WDW EM
SSB 0
LB 1.00 Hz
GB 0
PC 1.40
200 180 160 140 120 100 80 60 40 20 0 ppm
